# Coordinatively improving polymeric phosphorescence lifetime and quantum yield via triplet exciton modulation

**DOI:** 10.1038/s41467-026-74266-8

**Published:** 2026-06-11

**Authors:** Hui Hou, Zhian Cai, Jiayao Liu, Jun Zhang, Hao Wang, Xia Huang, Yuanjin Chen, Lunjun Qu, Yanli Zhao, Chaolong Yang

**Affiliations:** 1 School of Materials Science and Engineering, Chongqing, University of Technology, Chongqing, China; 2 School of Pharmacy and Bioengineering, Chongqing University of Technology, Chongqing, China; 3 School of Chemistry, Chemical Engineering and Biotechnology, Nanyang Technological University, 21 Nanyang Link, Singapore, Singapore

**Keywords:** Excited states, Supramolecular polymers, Polymers

## Abstract

The advancement of polymeric materials with exceptional room temperature phosphorescence (RTP) performance is hindered by the competing relationship between long phosphorescence lifetime (*τ*_P_) and high absolute phosphorescence quantum yield (*Φ*_P_). Herein, we present an Iodine-Stretch-Boric Acid (ISB) strategy that synergistically fine-tunes the generation and quenching of triplet excitons, thus enhancing both *τ*_P_ and *Φ*_P_. In this strategy, ISB harnesses the external heavy atom effects to facilitate spin-orbit coupling of phosphors, while simultaneously inducing an exceptionally dense cross-linked network within binaphthyl derivative-incorporated polyvinyl alcohol (*BINs@PVA*) films. This dual modulation engenders an optimization of the non-radiative rate constant (${k}_{{{{\rm{nr}}}}}^{P}$) and intersystem crossing rate constant (*k*_ISC_) of phosphorescence emission. Notably, the RTP performance of post-ISB treated *DFBN@PVA* films attain a notable advancement, with long *τ*_P_ of 1239.8 ms@528 nm (28.4-fold increase) and high absolute *Φ*_P_ of 33.8% (24.1-fold increase). Meanwhile, ISB strategy improves the durability of the *DFBN@PVA* films, preserving bright RTP emission after five days of water immersion and 12 hours in acidic, alkaline and hot water, exhibiting substantial potential for multicolor secure information, antibacterial, and water resistant RTP applications.

## Abstract (web summary)

Preparation of room temperature phosphorescent polymeric materials with high quantum yield and long lifetime is challenging but desirable. Here, the authors report a material combining iodine, boric acid and stretching to influence generation and quenching of triplet excitons for favorable properties.

## Introduction

Room temperature phosphorescence (RTP) materials have attracted much interest for potential applications in information storage, bioimaging and optical sensors. The development tendency of organic materials for RTP is toward long emission span and large Stokes shift^1–3^. Although organic RTP materials present pronounced advantages for practical applications owing to their ease of modification and cost-effectiveness, the primary limitations stem from inefficient intersystem crossing (ISC) between singlet and triplet excitons, impeded by weak spin-orbit coupling (SOC) coupled with the tendency of triplet excitons to undergo rapid non-radiative decay, thereby resulting in the quenching of phosphorescence emission^4–7^.

Notably, introducing heavy atom effects (HAE) into RTP systems, such as incorporating halogen bonds into phosphors or embedding them into a polymer matrix through molecular design^8–10^, often facilitates the ISC of triplet excitons, leading to efficient RTP emission. However, this method often comes with sacrificing phosphorescence lifetime (*τ*_P_), along with simultaneous acceleration of both ISC rate constant (*k*_ISC_) and phosphorescence rate constant (*k*_P_)^11–13^. For instance, Chi et al. achieved high phosphorescence quantum yields (*Φ*_P_) of 52.1% and a *τ*_P_ of 180 ms by modulating intramolecular halogen bonding through structural isomerism^10^. Liu et al. reported organic supramolecular pins composed of alkyl-bridged phenylpyridinium salts and cucurbit[8]uril (CB[8]), in which *Φ*_P_ achieved 99.38%, and *τ*_P_ was 110 ms^14^. Nevertheless, achieving highly efficient and long-lived phosphorescence emission through HAE manipulation remains a formidable challenge.

Extensive investigations into RTP phenomena have substantially advanced our understanding of the mechanisms underlying luminescence enhancement. These insights have enabled the design of several RTP materials exhibiting exceptional luminescent properties. While numerous studies have reported RTP materials with either high *Φ*_P_ (~100%) or ultralong *τ*_P_^14–19^, those that exhibit both characteristics concurrently remain exceedingly rare. This rarity is attributed to the trade-off between competing radiative and non-radiative decay processes. Therefore, achieving such dual-performance RTP emission is a highly coveted goal. Current strategies predominantly focus on optimizing energy transfer pathways and exploiting multi-confinement effects to achieve RTP emission with both high *Φ*_P_ and long *τ*_P_^20–22^.

**Fig. 1:**
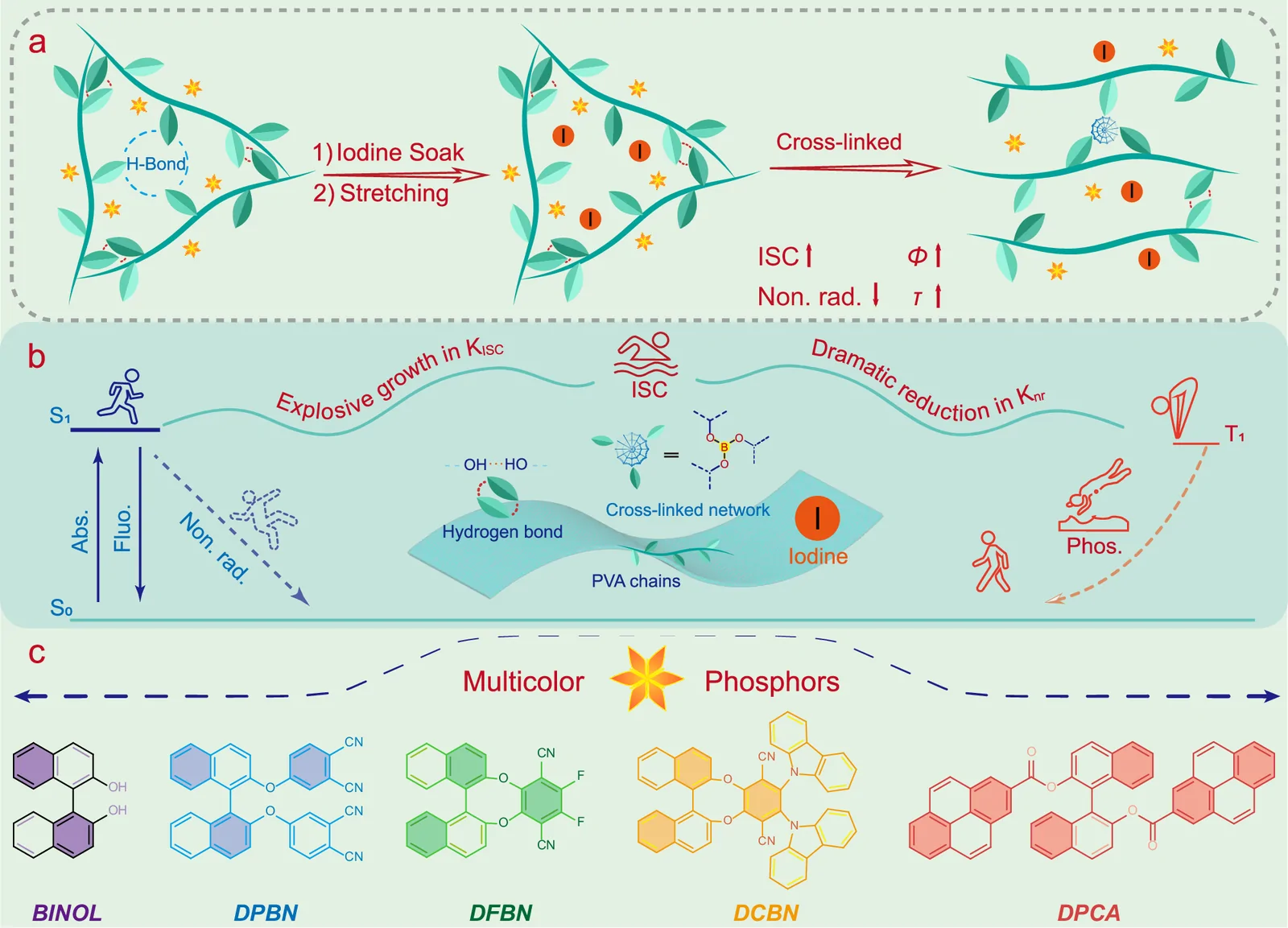
Schematic illustration of ISB enhancing RTP. **a** Strategy of ISB treatment for enhancing RTP performance. **b** Jablonski illustration of efficient and long-lived RTP emission via ISB and a schematic diagram of hydrogen bond, iodine, crosslink network and *PVA* chains. Abs. stands for absorption, Fluo. for fluorescence, Non. rad. for non-radiative, and Phos. for phosphorescence. **c** Chemical structure of *BINOL*, *DPBN*, *DFBN*, *DCBN* and *DPCA* molecules.

Thus, the key question is whether it is conceivable to devise a strategy that promotes highly efficient ISC through HAE without compromising phosphorescence lifetime. Drawing inspiration from the fabrication process of poly(vinyl alcohol) (*PVA*) polarizing films^23–25^, where *PVA* films are immersed in an iodine aqueous solution, followed by stretch-induced orientation and cross-linking mediated by *boric acid* (*BA*), we identify several key factors that significantly influence the RTP performance. Specifically, the cross-linked network and molecular orientation formed during this process provide a rigid environment that effectively restricts the intramolecular motion of triplet excited states, leading to a reduction of non-radiative decay and phosphorescence quenching rates. Meanwhile, iodine is incorporated into the *PVA* matrix and provides a HAE to stimulate ISC from the excited singlet to triplet states (Fig. 1). Building on these insights, we propose a strategy for designing RTP materials that simultaneously exhibit high *Φ*_P_ and long *τ*_P_, referred to as ISB.

In this work, we successfully synthesized four binaphthyl derivative-based molecules (*BINs*) and incorporated them into a *PVA* matrix (*BINs@PVA*) to prepare RTP films, achieving multicolor phosphorescence emission by the ISB strategy (Fig. 1a, c). Notably, we identify that ISB not only initiates the emergence of RTP but also affords both exceptional efficiency and substantially prolonged lifetime from scratch. Therefore, ISB can serve as a synergistical approach to concurrently enhance both *Φ*_P_ and *τ*_P_. While stretching also contributes to improving RTP performance, its role is more auxiliary, primarily facilitating the coordination of more iodine atoms with *PVA* during the stretching process. Ultimately, we achieve a breakthrough: transitioning from the complete absence of RTP to a long *τ*_P_ of 1239.8 ms and a high absolute *Φ*_P_ of 33.8% at room temperature. This outcome is accomplished through the collaborative manipulation of triplet exciton generation and quenching, along with the charge distribution and energy bandgap (*ΔE*_g_) of *BINs*, further underscoring the efficacy of the ISB strategy. The designed stimuli-responsive color-tunable *BINs@PVA* RTP materials demonstrate considerable promise for a range of potential applications, including anti-bacteria, multicolor display and underwater phosphorescence, thus paving the way for advancements in sophisticated optoelectronic devices.

## Results

### Synthesis and characterization

The four chromophores with a binaphthyl structure, namely *DPBN*, *DFBN*, *DCBN* and *DPCA*, were synthesized from a precursor (*BINOL*), as detailed in Fig. 1c, Supplementary Fig. 1. The structures of all *BINs* were thoroughly confirmed by the nuclear magnetic resonance (^1^H NMR and ^13^C NMR) spectra and high-resolution mass spectra (HRMS) (Supplementary Figs. 2–13). The four *BINs* in 2-methyltetrahydrofuran exhibit very weak and short phosphorescence at 77 K, with emission peaks at 497 nm for *DPBN*, 493 nm for *DFBN*, 489 nm for *DCBN* and 526 nm for *DPCA*, suggesting multicolor luminescence potential (Supplementary Fig. 14, Supplementary Movie 1). Meanwhile, the absorption spectra of *BINs* in *CH*_*2*_*Cl*_*2*_ show similar strong absorption peaks at the high-energy UV region (230-250 nm), which should be attributed to the π-π* transitions of the binaphthyl moiety (Supplementary Fig. 15). Subsequently, a series of multicolor RTP films was prepared via incorporating *BINs* into a *PVA* matrix, followed by ISB treatment. The detailed experimental procedures are shown in the “Methods” section. Prior to ISB treatment, not only *BINs@PVA* films but also *BINOL@PVA* films displayed very weak RTP emission after the UV lamp was turned off (Supplementary Fig. 16). In contrast, vibrant multicolor RTP emission was successfully obtained following ISB treatment (Supplementary Movie 2).

### Photophysical properties of *BINs@PVA* films

After identifying the optimal doping concentration and the most favorable ISB conditions (Supplementary Figs. 17, 18), we performed comprehensive photophysical characterizations on *BINs@PVA* films by selecting the *BINs@PVA* films that exhibited the optimized RTP performance for each system. Unless otherwise stated, the pre-ISB *BINs@PVA* films refer to those before ISB treatment, while the post-ISB *BINs@PVA* films correspond to those exhibiting significant performance after ISB treatment.

**Fig. 2:**
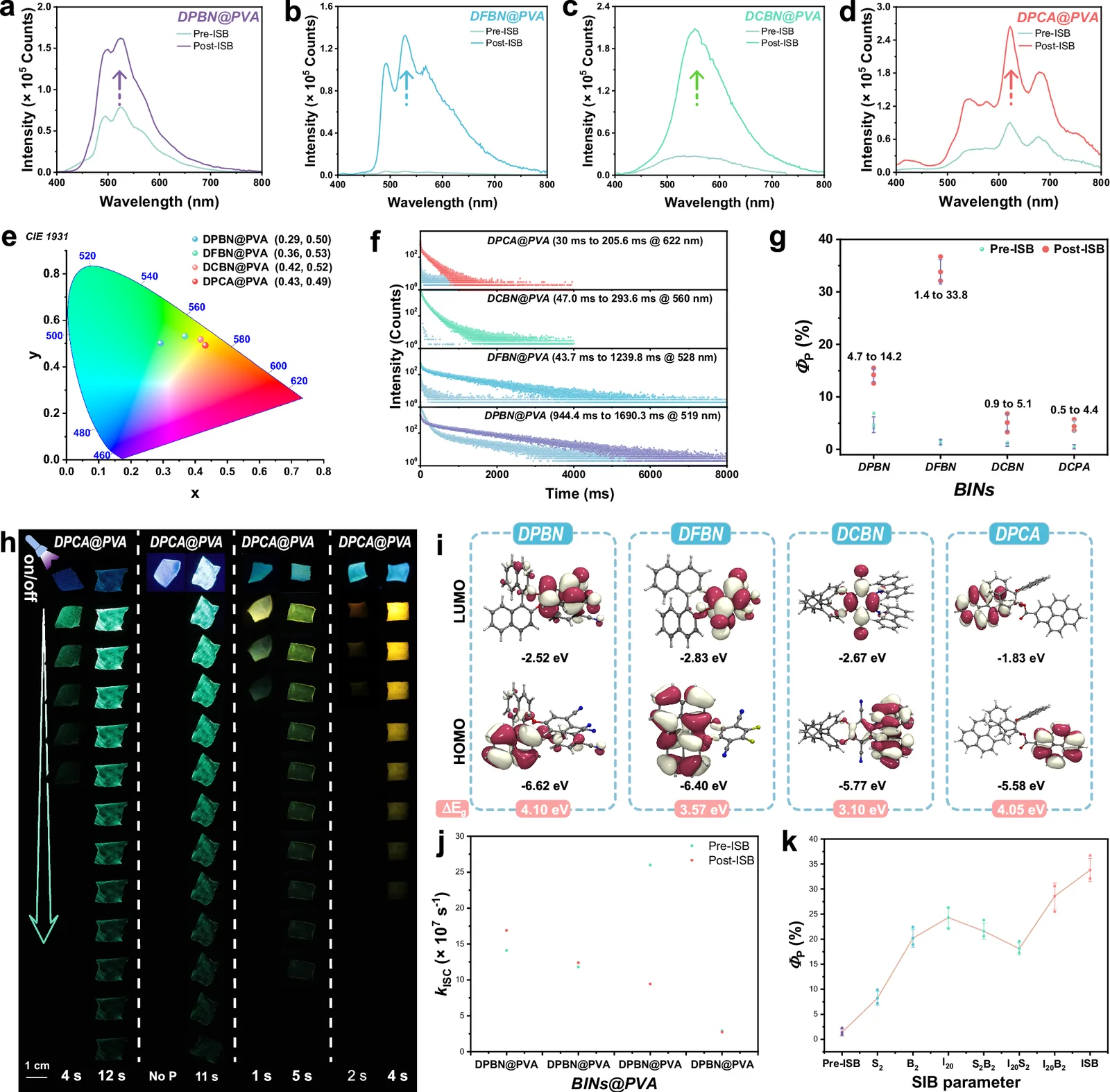
Photophysical properties of *BIN* films. Time-gated spectra of **a** *DPBN@PVA*, **b** *DFBN@PVA*, **c** *DCBN@PVA*, and **d** *DPCA@PVA* films. The dashed line attributes to pre-ISB treatment and the solid line attributes to post-ISB treatment. **e** CIE coordinate diagram corresponding to the time-gated spectra of *BINs@PVA* films. **f** Lifetime decay curves of pre-ISB and post-ISB *BINs@PVA* films. **g** Absolute *Φ*_P_ of pre-ISB and post-ISB *BINs@PVA* films (Error bar = standard deviation, *n* = 3). **h** Afterglow photographs of pre-ISB and post-ISB *BINs@PVA* films. **i** HOMO and LUMO distributions of the optimized and simplified *BINs*. **j** *k*_ISC_ of pre-ISB and post-ISB *BINs@PVA* films, and the calculated data are from Supplementary Table 1. **k** Absolute *Φ*_P_ of *DFBN@PVA* films with varying ISB treatments (Error bar = standard deviation, *n* = 3).

Noteworthy changes were observed in the steady-state photoluminescence spectra of post-ISB *BINs@PVA* films. The subtle shift suggests that ISB treatment induces a reconfiguration of the electron distribution within *BINs@PVA* films. The fluorescence emission peaks undergo the following shifts: *DPBN@PVA* from 459 to 484 nm, *DFBN@PVA* from 458 to 454 nm, *DCBN@PVA* from 496 to 536 nm, and *DPCA@PVA* remains at 524 nm with a slight change in the fluorescence peak shape. In addition, the fluorescence lifetime of *BINs@PVA* films exhibits a nanosecond character (Supplementary Fig. 19, Supplementary Table 1). Prior to ISB treatment, the *BINs@PVA* films, except for the *DPBN@PVA* film, exhibited no visible afterglow to the naked eye after removing the 6 W UV lamp. Following ISB treatment, these RTP films showed the capability to emit a range of colors, presenting a persistent afterglow up to 11 s (Supplementary Movie S1). Upon removal of the excitation source, the post-ISB *DPBN@PVA*, *DFBN@PVA*, *DCBN@PVA*, and *DPCA@PVA* films exhibited cyan (517 nm), green (528 nm), orange (560 nm), and red (622 nm) afterglow emission, corresponding to Commission Internationale de l'Éclairage (CIE) color coordinates of (0.29, 0.50), (0.36, 0.53), (0.42, 0.52), and (0.49, 0.44), respectively (Fig. 2a–e, Supplementary Fig. 20). Therefore, we categorize these delayed photoluminescence phenomena as phosphorescence, and the corresponding lifetime profiles along with time resolved emission spectroscopy (TRES) support this classification, thereby confirming the accuracy of the observed afterglow characteristics (Fig. 2f, Supplementary Fig. 21). Furthermore, the integration of HAE led to a pronounced augmentation in the *Φ*_P_ of the post-ISB *BINs@PVA* films, accompanied by a marked prolongation of the afterglow duration, offering compelling evidence that ISB treatment substantially enhances the efficiency of RTP emission (Fig. 2g, h, Supplementary Figs. 22, 23). The photophysical properties and kinetic rate constants of the pre-ISB and post-ISB *BINs@PVA* films are listed in Supplementary Table 1.

Meanwhile, the temperature dependent time-gated spectra and lifetime decay profiles confirm that all *BINs@PVA* films exhibit RTP characteristic rather than thermally activated delayed fluorescence (TADF) (Supplementary Fig. 24). The calculated energy gaps (*ΔE*_g_) between highest occupied molecular orbital (HOMO) and lowest unoccupied molecular orbital (LUMO) for *DPBN, DFBN, DCBN* and *DPCA* are 4.10, 3.57, 3.10, and 4.05 eV, respectively (Fig. 2i). The nearly complete spatial separation between HOMO and LUMO distributions indicates that a charge transfer (CT) transition is likely to occur upon excitation. The long, persistent and high-efficiency multicolor RTP emission of post-ISB *BINs@PVA* films may be intrinsically associated with the promoted *k*_ISC_ and lower phosphorescent non-radiative decay rate constant (${k}_{{{{\rm{nr}}}}}^{P}$), as evidenced by kinetic calculations (Fig. 2j, Supplementary Table 1), thereby markedly improving *Φ*_P_ and *τ*_P_ (Fig. 2f, k, Supplementary Table 1). Furthermore, the dramatic enhancement in *k*_ISC_ following the ISB treatment, particularly for *DFBN@PVA,* where *k*_ISC_ increased by over 23-fold, can be principally attributed to the incorporation of iodine. Simultaneously, the ${k}_{{{{\rm{nr}}}}}^{P}$ of *DFBN@PVA* decreased by more than a factor of 39, which can be primarily ascribed to the formation of a robust, highly compact cross-linked network between *BA* and *PVA*. Consequently, *DFBN@PVA* exhibited a vivid green afterglow lasting up to 11 s under a 6 W UV lamp. Generally, the quantum yield and lifetime of phosphorescence are inversely correlated due to the trade-off between competing radiative and non-radiative decay processes. However, both the quantum yield and lifetime of the four *BINs@PVA* films were markedly enhanced following ISB treatment, demonstrating a substantial improvement in their RTP properties.

**Fig. 3:**
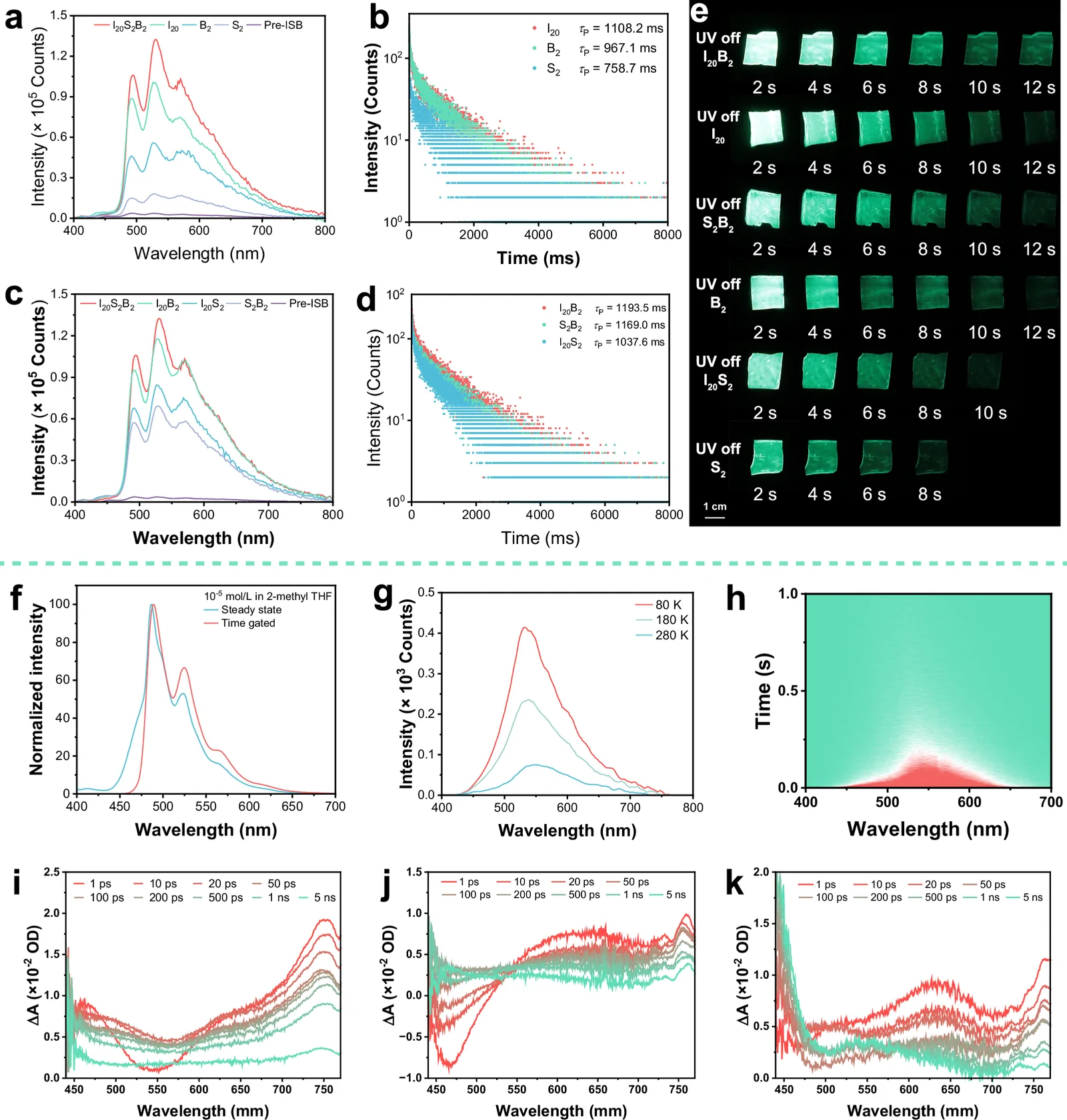
Elucidating the distinctive features of *BINs.* **a** Phosphorescence spectra and **b** decay curves of *DFBN@PVA* film under I_20_, B_2_, and S_2_ treatment, respectively. **c** Phosphorescence spectra and **d** decay curves of *DFBN@PVA* film under I_20_B_2_, I_20_S_2_, and S_2_B_2_ treatment, respectively. Curves indicated for I_20_S_2_B_2_ and Pre-ISB in **a** and **c** are the same, respectively. **e** Afterglow photographs of *DFBN@PVA* films under various treatments. **f** Steady-state PL spectra and time-gated spectra of *DCBN* in 2-methyltetrahydrofuran (10 μM) at 77 K. **g** Temperature-dependent time-gated spectra of *DCBN@PVA* film. **h** TRES of *DCBN@PVA* film. Transient absorption spectra of (**i**) isolated *DCBN* dispersed in *DMSO*, **j** pre-ISB *DCBN@PVA* film and **k** post-ISB *DCBN@PVA* film, ranging from 1 ps to 5 ns.

To further elucidate the impact of ISB treatment on RTP performance, control experiments were conducted. We prepared a series of *DFBN@PVA* films with I_20_, S_2_, B_2_, I_20_S_2_, S_2_B_2_ and I_20_B_2_ treatment. Treating the *DFBN@PVA* films individually with *iodine* staining (I), stretching (S), or *BA* treatment (B) could each positively enhance and promote the RTP performance (Fig. 3a–c). Only the one with I_20_S_2_ treat decreased the RTP performance, which can be explained that stretching promotes the adsorption of iodine by *PVA*, and the concentration of iodine in the *PVA* matrix is too high to result in fast *k*_P_ for quenching RTP. Others play a promoting role in RTP performance but are inferior to ISB treatment (Fig. 3c, d). The enhancement provided by *iodine* and *BA* is predominant, while stretching plays a supplementary role in assisting RTP enhancement and iodine adsorption^26^. Thus, following the ISB treatment, the synergistic interactions among HAE, molecular orientation and the cross-linked network facilitate ISC while attenuating *k*_nr_, indicating that the observed enhancement should be resulted from the holistic interactions of the organic system rather than the isolated contribution of individual components. Although some studies have shown that the introduction of heavy atoms does not inevitably shorten phosphorescence lifetime and our previous work has illustrated that a cross-linked network could partially enhance ISC^6,22,26–28^, this work is still a rare example for synergistically enhancing RTP *Φ*_P_ and *τ*_P_ via ISB treatment.

In addition, we noticed that the time-gated spectra of *DPCA@PVA* films were not accordant with the afterglow color. To exclude impurity-induced RTP emission^29^, the *DPCA* molecule was subjected to additional purification using silica gel column chromatography to ensure high purity for subsequent characterization. Intriguingly, both the phosphorescence intensity and lifetime exhibited a modest increase corresponding to the times of purification cycles applied (Supplementary Fig. 25), which could be attributed to the slight variation in doping concentration. However, the afterglow color of *DPCA@PVA* still did not fully match the time-gated emission profile. To analyze this discrepancy, 622 nm was selected as the reference peak, and *τ*_P_ was measured at intervals of 20 nm. As shown in Supplementary Fig. 25c, *τ*_P_ increased, accompanied by a blue shift in the emission peak. These findings could collectively explain that the afterglow color of *DPCA@PVA* does not fully align with the time-gated spectra.

A perplexing observation is that these *BINs* exhibit faint and transient afterglow signals even at 77 K (Supplementary Movie 2). Under ideal conditions, their afterglow duration time should be capable of that of the *BA*-treated films, given that both the low-temperature environment and the cross-linked network induced by BA treatment inherently provide a rigid matrix to mitigate ${k}_{{{{\rm{nr}}}}}^{P}$ and facilitate the extended RTP lifetimes. To delve deeper into this phenomenon, *DCBN* was chosen as a model molecule, and femtosecond transient absorption spectroscopy (fs-TA) was employed to examine the transition from TADF to RTP (Fig. 3f–h), for which *DCBN* emits TADF at the isolated state^30^, but emits RTP at the film state.

Within the 1 ps to 5 ns time window, the fs-TA spectra resolve two quasi-simultaneously generated excited-state absorption (ESA) bands centered at approximately 450 and 757 nm (Fig. 3i). The nearly synchronous temporal evolution of these two ESA features is emblematic of TADF^31,32^, consistent with their assignment to a rapid RISC process. By contrast, the pre-ISB *DCBN@PVA* film exhibits a gradual attenuation of the stimulated emission (SE) signal near 460 nm, accompanied by the emergence of a weak ESA feature (Fig. 3j), in line with its comparatively inefficient RTP (Fig. 2k). The presence of well-defined isosbestic points in the fs-TA spectra of both pre-ISB and post-ISB *DCBN@PVA* films substantiates a clean transition between the lowest singlet and triplet excited-state manifolds. Upon ISB treatment, a pronounced ESA band rapidly develops at around 450 nm in the *DCBN@PVA* film (Fig. 3k). Relative to the untreated counterpart, this marked enhancement is plausibly ascribed to the iodine-induced HAE, which effectively accelerates ISC.

Notably, the ESA signals at around 450 and 761 nm display opposite temporal evolution trends, associated with the characteristic triplet-state population dynamics in phosphorescence. From a kinetic standpoint, the 450 nm feature is attributable to the lowest triplet excited state, whereas the 757 nm band originates from the singlet excited state. The pronounced shift in the SE signal of *DCBN*, transitioning from the isolated state to pre-ISB and post-ISB *DCBN@PVA* films, is ascribed to solid-state solvation effects, with the isosbestic points indicating that dielectric-controlled dynamics predominantly govern this process. Time-resolved absorption spectra provide additional support for this assignment (Supplementary Fig. 26a–c). Quantitative analysis of the singlet state dynamics was performed by fitting the fs-TA kinetic traces extracted from the ESA regions using a biexponential decay model (Supplementary Fig. 26d–f). Compared with the pre-ISB film, the post-ISB-treated *DCBN@PVA* film exhibits a distinctly shortened singlet excited state lifetime concomitant with a prolonged triplet-state persistence. This kinetic reconfiguration underscores the role of ISB as an efficient ISC accelerator, thereby favoring RTP emission.

Further insight is gained by spanning 1 fs to 1 ps. Isolated *DCBN* displays an ultrafast RISC process within this time window (Supplementary Fig. 27a), whereas film-state *DCBN* is characterized by a pronounced SE signal. Given the close spectral proximity between the SE band and the lowest triplet ESA feature (Supplementary Fig. 27b), this behavior indicates that RISC is substantially suppressed in the film state, with the weak triplet ESA signal being masked by SE. In ISB-treated *DCBN* films, a distinct triplet ESA feature emerges within 1 ps (Supplementary Fig. 27c). As evidenced by Supplementary Fig. 19e, ISB treatment effectively modulates the *ΔE*_ST_ of *DCBN*. When combined with temperature-dependent time-gated spectra (Supplementary Fig. 24), these observations unequivocally demonstrate that the afterglow emission of ISB-treated *DCBN@PVA* originates from RTP rather than TADF. Consequently, the rapid emergence of the triplet ESA signal can be unambiguously attributed to ISB-promoted ISC, rather than RISC.

### Mechanism studies of ISB enhancing RTP

**Fig. 4:**
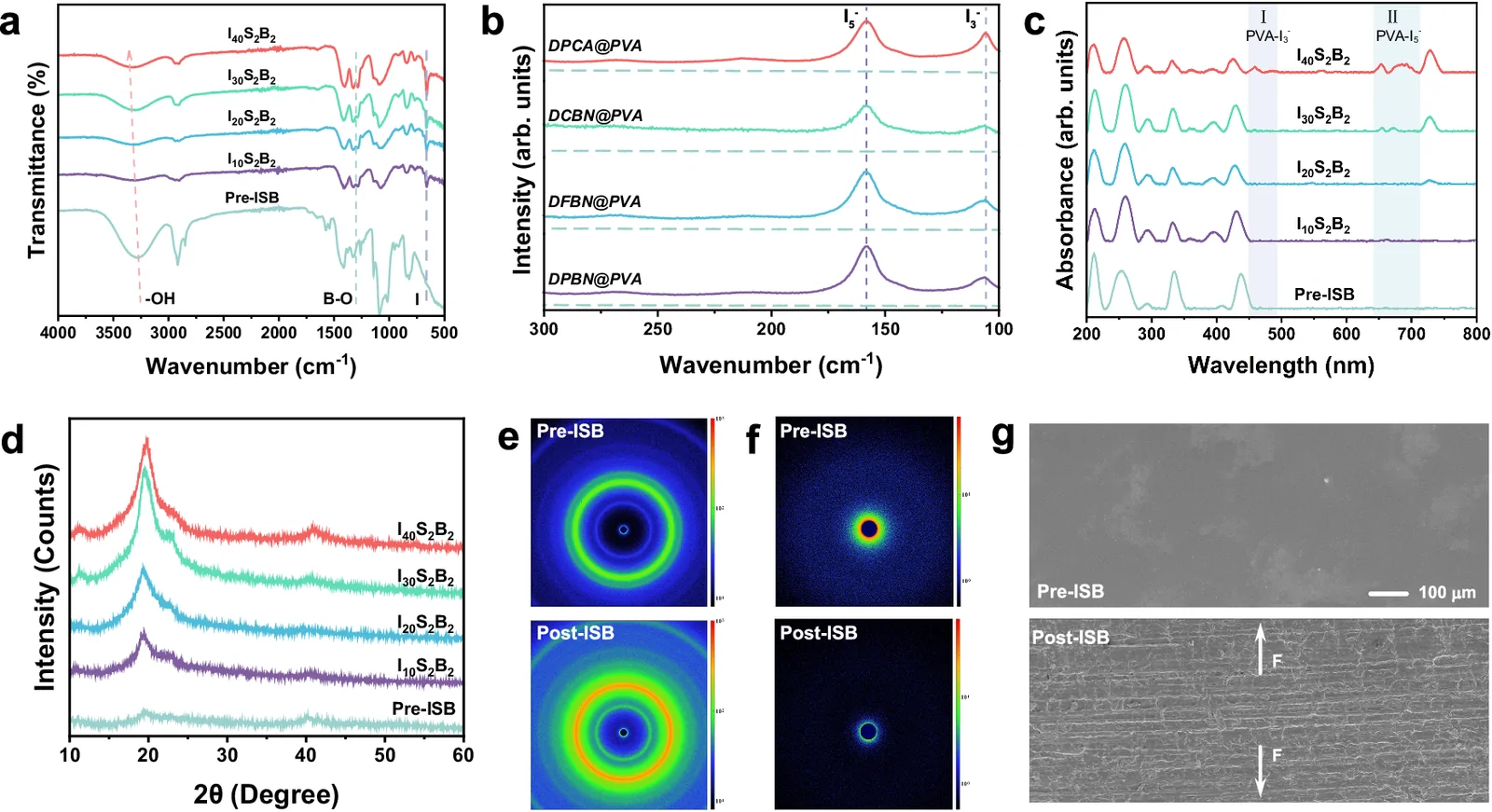
Mechanism study of ISB enhancing RTP via chemical species and structural transformation. **a** FT-IR of *DFBN@PVA* films with various ISB treatments. **b** Raman spectra of post-ISB *BINs@PVA* films. **c** Solid state UV-Vis of *DFBN@PVA* films with varying ISB treatment. **d** Powder XRD patterns of *DFBN@PVA* films with different ISB treatment. **e** 2D WAXS patterns of pre-ISB and post-ISB *DFBN@PVA* films. **f** 2D SAXS patterns of pre-ISB and post-ISB *DFBN@PVA* films. **g** SEM images of pre-ISB and post-ISB *DFBN@PVA* films.

To explore the impact of ISB on the luminescent properties of *BINs@PVA* films, we performed several tests to assess the incorporation of iodine and *BA* within the *PVA* matrix following ISB treatment. According to FT-IR spectra (Fig. 4a, Supplementary Fig. 28), a prominent B–O vibration peak at 1325 cm^−1^, and the characteristic iodine ion absorption peak at 660 cm^−1^ were observed following ISB treatment, confirming the successful incorporation of iodine and boron elements into the *PVA* matrix. The absorption peak around 3300 cm^−1^ should be attributed to the stretching vibration of hydroxyl groups (−OH) involved in hydrogen bonding, shifting to a higher wavenumber upon ISB treatment. This shift is due to the interaction of iodine ions (*I*_3_^−^, *I*_5_^−^) and *BA* with the oxygen of −OH groups in *PVA*, weakening both intermolecular and intramolecular hydrogen bondings^33,34^. Raman spectra of post-ISB *BINs@PVA* films showed two strong scattering peaks (Fig. 4b), suggesting that iodine elements primarily exist in the forms of *I*_3_^−^ (106 cm^−1^) and *I*_5_^−^ (160 cm^−1^) in the amorphous region of *PVA* after ISB treatment^35^. In the preparation process of post-ISB *BINs@PVA*, the iodine concentration was estimated to be approximately 0.05 M, which is consistent with the Raman spectral features reported for *PVA* films soaked in low-concentration *I*_*2*_/*KI* solutions in the literature^36^. Therefore, it is hypothesized that both *I*_3_^−^ and *I*_5_^−^ ions are efficient in enhancing the RTP performance of *BINs@PVA* films. In contrast, Raman spectra of only iodine-treated *DFBN@PVA* film lacked these peaks, further supporting that stretching enhances iodine adsorption by *PVA* (Supplementary Fig. 29)^25,37^.

However, Raman spectra can only indicate changes in the concentrations of *I*_3_^−^ and *I*_5_^−^ ions, without distinguishing whether the polyiodide ions are in a free state or complexes with *PVA* molecular segments. Therefore, we conducted solid-state UV-Vis spectra for *BINs@PVA* films (Fig. 4c, Supplementary Fig. 30). The absorption peaks in regions I and II were enhanced with prolonged iodine staining, implying the gradual formation of *PVA-I*_*3*_^−^ and *PVA-I*_*5*_^−^ complexes in the amorphous region^37,38^. The *PVA-I* complexes could function as junction points within the *PVA* network^39^. As shown in Supplementary Tables 2, 3, quantitative determination of iodine and boron in the *BIN@PVA* films was carried out using inductively coupled plasma-mass spectrometry (ICP-MS), and relative test parameters are summarized in Supplementary Table 4. A substantially higher phosphor-to-boron (~0.02) molar ratio was observed as compared with that of phosphor to iodine (~10), consistent with the difference in processing time. Specifically, a relatively high iodine content was observed, which may constitute one of the key factors contributing to the markedly enhanced ISC process in the phosphorescent system. Stretching also promotes the formation of a crystalline structure after ISB treatment, as evidenced by the marked increase in peak intensity at 2θ = 20° in the powder XRD pattern (Fig. 4d, Supplementary Fig. 31). In addition, the broadened diffraction peak around 2θ = 20° in the stained films with the prolonged ISB treated time reveals the lattice parameters of *PVA* crystals progressively contract, consistent with the relationship between peak width and lattice size proposed by Hosemann^40^.

The enhancement of 2D WAXS diffraction rings and the shift observed in the 1D WAXS profiles further corroborate this finding (Fig. 4e, Supplementary Fig. 32a). Furthermore, the reduction in 2D SAXS diffraction rings and the position of the scattering peaks at q~0.06 Å shifting to higher q values in 1D SAXS indicate a decline in both the long-range periodicity and crystal thickness of *DFBN@PVA* films (Fig. 4f, Supplementary Fig. 32b), likely due to the remolding of *PVA* crystalline order by the incorporation of iodine. Differential scanning calorimetry (DSC) analysis shows that the decrease in melting temperature (*T*_m_) results from iodine disrupting *PVA* hydrogen bonds (Supplementary Fig. 33). Alternatively, a pronounced increase in glass transition temperature (*T*_g_) after ISB treatment is attributed to BA-induced cross-linking that restricts *PVA* chain mobility. This observed phenomenon can be ascribed to the situation that the penetration of iodine ions into *PVA* films during ISB attenuates the intermolecular hydrogen-bonding interactions and compromises the stability of the crystalline domains through coordination with hydroxyl moieties along the polymer chains, thereby provoking deformation of the lamellar structures and a reduction in lattice parameters^40^. Furthermore, the thermal decomposition of temperature (*T*_d_) of post-ISB *BINs@PVA* films is markedly elevated, indicating a substantial improvement in thermal stability following ISB treatment (Supplementary Fig. 34). Moreover, SEM images of *BINs@PVA* films following ISB treatment reveal distinct orientation perpendicular to the stretching direction, providing further corroboration of this structural transformation (Fig. 4g, Supplementary Fig. 35). In addition, *X*-ray photoelectron spectroscopy (XPS) was employed to further assess the structural stability of the *BIN* films. As shown in Supplementary Fig. 36, the N 1 *s* signal of the ISB-treated films appears markedly attenuated. This attenuation is attributed to the enhanced electrostatic interactions between the *PVA* matrix and the −CN groups induced by the ISB treatment, rather than structural degradation of the phosphors. In conclusion, ISB treatment profoundly influenced the configuration and conformation of *PVA* chains within *BINs@PVA* films, inducing a substantial enhancement in the thermal stability. This structural modification is likely a fundamental factor underlying the extraordinary improvement in the RTP performance of the *BINs@PVA* films.

**Fig. 5:**
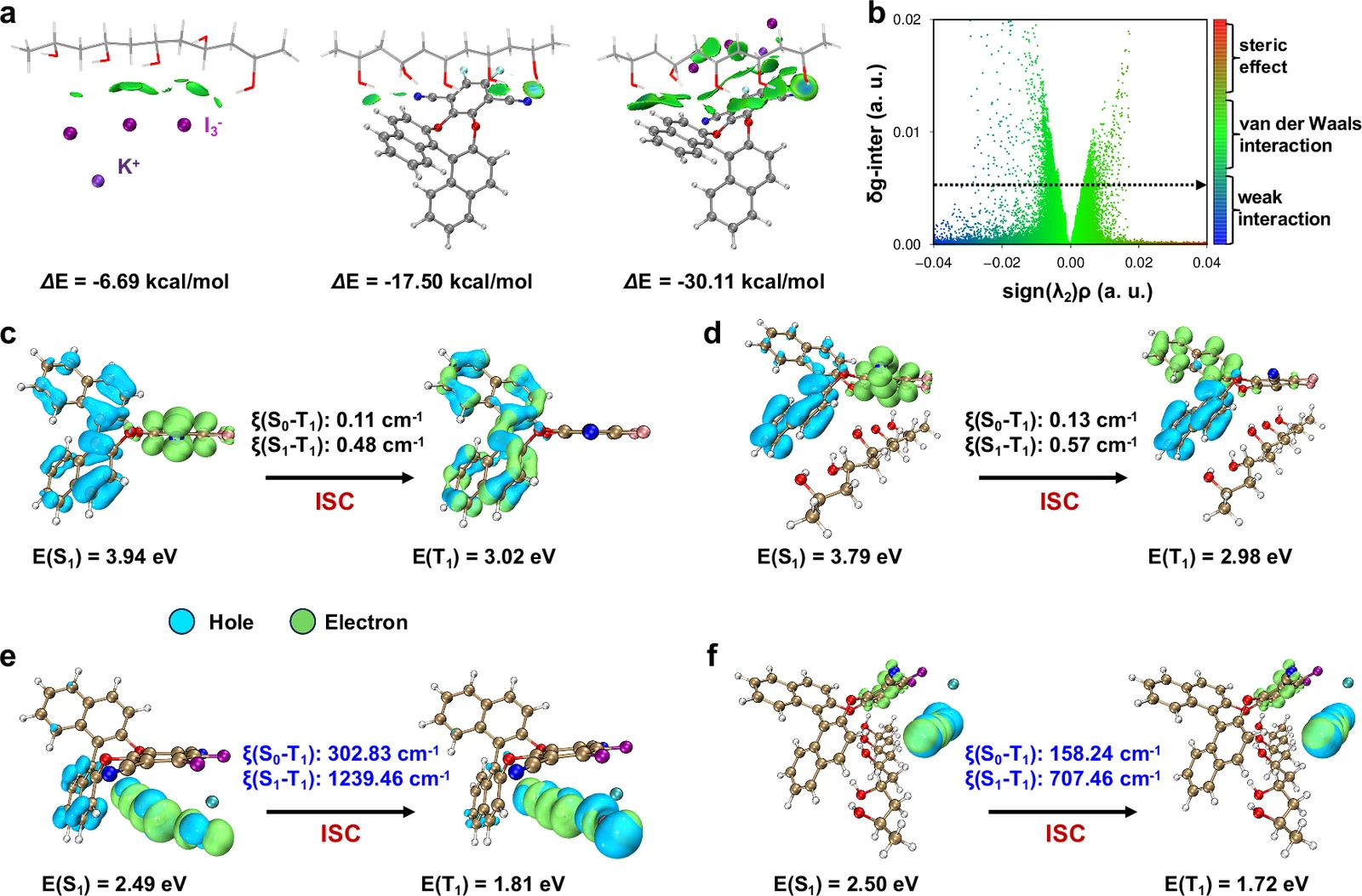
Mechanism study of ISB enhancing RTP via theory calculations. **a** Intermolecular interaction energies (considered on BSSE corrections) and IGMH analysis (*δ*_g-inter_ = 0.005 a. u.) of *PVA* model with *KI*_*3*_, *DFBN*, or *DFBN-I*_*3*_ model. **b** Distribution of different interactions between *DFBN* and *KI₃@PVA* by IGMH scatter map. Electron-hole analysis, SOC constants, and energy levels of the S_1_ and T_1_ states for (**c**) *DFBN*, **d** *DFBN@PVA*, **e** *DFBN-KI*_*3*_, and **f** *DFBN-KI*_*3*_*@PVA* models based on their S_0_ optimized geometry.

To gain deeper insight into the potential mechanism of ISB enhancing RTP, we conducted density functional theory (DFT) to analyze the interactions between phosphors and polymer matrix, and the time-dependent DFT (TD-DFT) calculations to analyze the excited state energy levels and spin-orbit coupling matrix element (SOCME) values between S_1_ and T_1_ states. The simplified theoretical model, *DFBN-KI*_*3*_*@PVA*, was developed to simulate the post-ISB *DFBN@PVA* films. The computational models, especially the positions of *I*_3_^−^ are arrayed randomly in the one-dimensional column parallel to the *PVA* chain axis, were constructed based on the reported literature^36,41^. The independent gradient model based on Hirshfeld partition (IGMH) analysis was employed to reveal weak interactions in the post-ISB-treated *DFBN@PVA* films (Fig. 5a). The intermolecular interaction energy corrected by the basis set superposition error (BSSE) between *DFBN* and *KI*_*3*_*@PVA* (−30.11 kcal/mol) was stronger than that between *PVA-KI*_*3*_ (−6.69 kcal/mol) and *DFBN-PVA* (−17.50 kcal/mol), underscoring the strong binding interaction in the *BINs-KI*_*3*_*@PVA* complex system. As shown in Fig. 5b, the IGMH scatter maps reveal that van der Waals interactions (green regions) dominate, with minor contributions from hydrogen bonding and electrostatic interactions (blue regions) between the phosphors and the polymer matrix, consistent with the XPS observation that electrostatic interactions between *BINs* and *PVA* are enhanced (Supplementary Fig. 36). Theoretical calculations show that ISB endows multiple interactions in *DFBN@PVA* systems to increase structural rigidity and promote phosphorescence emission^42^. As shown in Fig. 2i, the spatial separation of the LUMO and HOMO orbitals in *BINs* suggests a distinct electron density distribution, potentially affecting the electronic interactions within the *DFBN@PVA* RTP systems.

The analysis of electron-hole distribution (Fig. 5c) indicates that the electronic configurations of the S_1_ states of DFBN are characterized by CT ^1^π-π* transitions, while the T_1_ states are dominated by local excited (LE) ^3^π-π*. In contrast, for *DFBN-KI*_*3*_*@PVA*, the S_1_ state corresponds to a CT ^1^n-π* transition and the T_1_ state adopts a CT ^3^n-π* transition, accompanied by a decrease in the energy difference between S_1_ and T_1_ states (*ΔE*_ST_) from 0.92 eV to 0.78 eV. The charge distribution from *DFBN*, *DFBN@PVA*, *DFBN-KI*_*3*_ to *DFBN-KI*_*3*_*@PVA* undergoes changes, indicating that ISB drives the system towards a configuration more favorable for RTP emission (Fig. 5c–f). The SOCME values between S_1_ and T_1_ states based on different iodine models (*I*^−^, *I*_2_, *I*_3_^−^, and *I*_*5*_^−^) in *DFBN* were calculated (Supplementary Fig. 37). Although the incorporation of iodine enhanced the ISC process, *DFBN-KI*_*3*_ exhibited the largest SOC constants rather than *DFBN-KI*_*5*_, indicating that the regulation of iodine concentration is very important for ISC. This observation corresponds to the results of the control experiments. Furthermore, the SOCME values for *DFBN*, *DFBN@PVA*, *DFBN-KI*_*3*_ and *DFBN-KI*_*3*_*@PVA* revealed an exponential increase from 0.89 cm^−1^ to 707.46 cm^−1^ in the SOCME for the S_1_-T_1_ transitions. Thus, the calculations indicate that ISB promotes *K*_ISC_, leading to high quantum yield RTP emission. The similar conclusion holds for the other three *BIN* systems (Supplementary Figs. 38–40). In addition, we integrated the other eight phosphors into *PVA* and subjected them to ISB treatment, leading to a dramatic augmentation in both RTP lifetime and intensity for all systems. Specifically, the RTP lifetime of *9-fluorenemethanol* extraordinarily increased from 60.1 ms to 3241.3 ms, demonstrating the exceptional versatility of the ISB treatment in enhancing RTP performance (Supplementary Figs. 41, 42).

### Applications to harsh environmental conditions

**Fig. 6:**
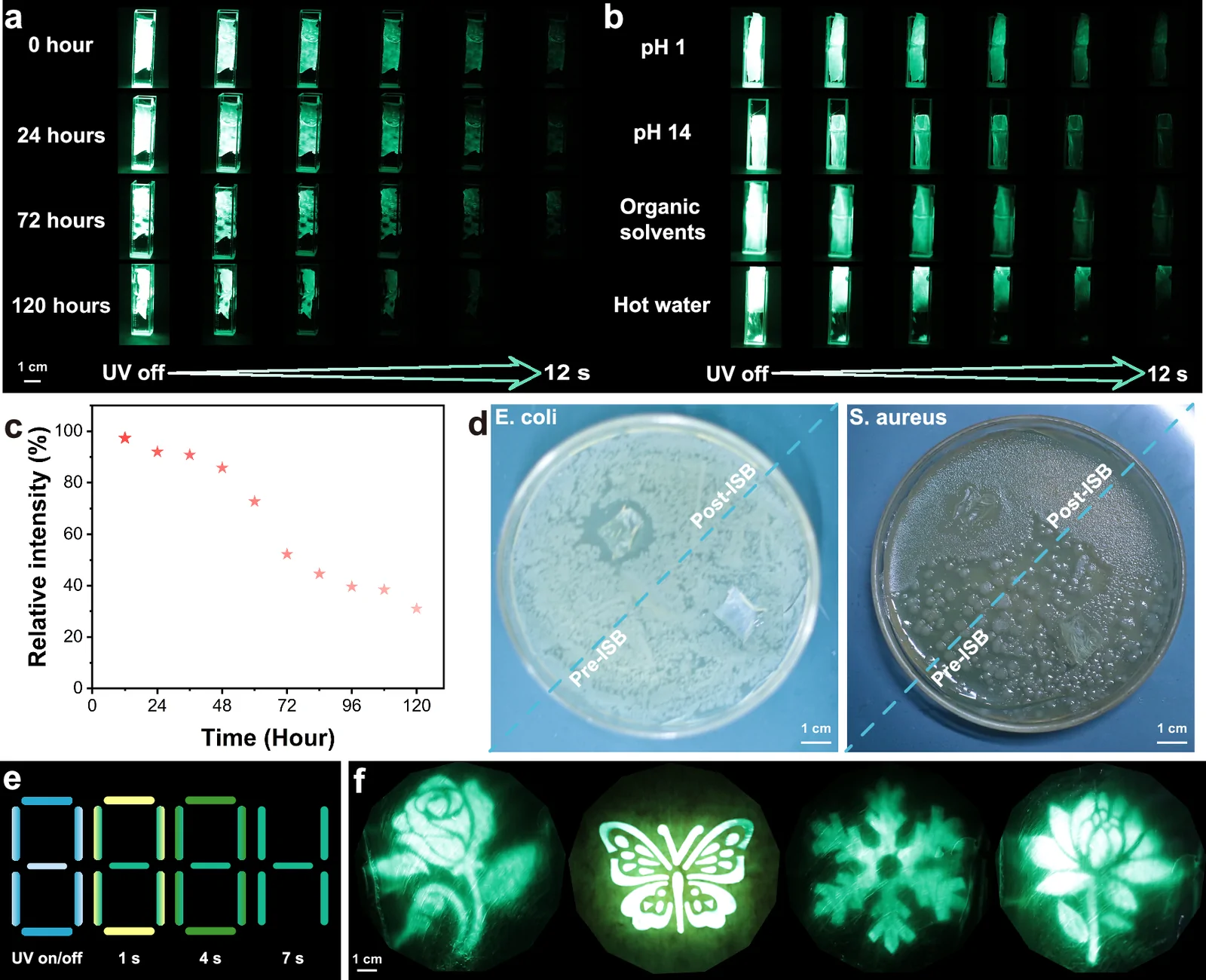
Applications based post-ISB *DFBN@PVA* films. **a** Afterglow photographs of post-ISB *DFBN@PVA* films immersed in water for a period. **b** Afterglow photographs of post-ISB *DFBN@PVA* films immersed in *HCl* (pH 1), *NaOH* (pH 14), mixed solvents (*DCM*: *DMF*: *DMSO*: *EtAc* = 1:1:1:1), and 100 °C water for 10 min. Notes: *DFBN@PVA* films were subjected to irradiation using a UV lamp to augment the luminance intensity in the afterglow photographs. **c** Phosphorescence intensity decay rate of post-ISB *DFBN@PVA* films. **d** Antibacterial tests of post-ISB *DFBN@PVA* films by the inhibition ring experiment. **e** Schematic representation of dynamic anti-counterfeiting. The number 8 is fabricated from two RTP materials, namely *DFBN@PVA* and *DPCA@PVA*. Under ultraviolet excitation, it mainly appears as a blue number 8. However, when the ultraviolet light stops exciting (less than 4 s), the number 8 shows a mixture of yellow and blue colors. After 4 s, the number 8 transforms into the letter H as the afterglow of *DPCA@PVA* film faded out. **f** Potential applications of *DFBN@PVA* films in complex pattern displays.

Considering the exceptional RTP performance of *DFBN@PVA* films and the high efficacy of the ISB strategy, we explored their potential applications. Initially, thermal stability evaluation confirms that the decomposition temperatures of all *BINs* exceed 200 °C, excluding thermal decomposition during the ISB process (Supplementary Fig. 43). ISB simulations further indicate that the structural integrity of *BINs* is preserved throughout the ISB treatment (Supplementary Figs. 44, 45). These features make *BINs* promising systems for practical applications. Specifically, the compact cross-linked network formed in post-ISB *DFBN@PVA* films imparts substantial stability, rendering them highly resistant to harsh environmental conditions. Their afterglow persists for several seconds over a span of 5 days in water and remains bright even after 12 h of immersion in harsh solvents (Fig. 6a–c, Supplementary Figs. 46, 47). This feature can be attributed to the ultra-stable structure endowed by ISB treatment, allowing the RTP films to remain stable even at high temperature (Supplementary Fig. 48), in which iodine-treated *DFBN* films exhibit thermal stability comparable to that of the *BA-*treated counterparts, an outcome that further substantiates the role of *iodine* species as effective junctions within the *PVA* matrix. Throughout a full heating-cooling cycle, the post-ISB *DFBN@PVA* film retained discernible afterglow even at temperatures exceeding 150 °C (Supplementary Fig. 49), and its emission performance was almost fully restored to the initial state after the cycle, representing a rare example among polymer-based RTP materials. Furthermore, a distinct antibacterial ring forms surrounding the post-ISB *DFBN@PVA* films, evidencing their bactericidal efficacy against both Gram-negative bacteria (E. coli) and Gram-positive bacteria (S. aureus) with noticeable growth inhibition (Fig. 6d). Furthermore, we fabricated a pattern using post-ISB *DFBN@PVA* and *DPCA@PVA* films, where the displayed information evolves dynamically following the cessation of UV excitation, highlighting substantial potential of the films in dynamic anti-counterfeiting applications (Fig. 6e). We also showcased the capability of post-ISB *DFBN@PVA* films in designing intricate phosphorescence patterns (Fig. 6f).

## Discussion

In conclusion, we introduce a strategy, termed ISB, which not only advances the understanding of RTP material design but also offers a pathway to overcome the inherent challenges in achieving both high *Φ*_P_ and extended lifetime. Notably, *DFBN@PVA* films exhibit considerable dual-performance phosphorescence emission with a *τ*_P_ of 1239.8 ms and an absolute *Φ*_P_ of 33.8%. The structural analysis and theoretical calculations reveal that ISB precisely modulates the cross-linked structure and electron configuration within *BINs@PVA* films via ISB, offering a promising platform for future development of efficient, stable, and multifunctional RTP materials. Furthermore, the exceptional resilience of post-ISB *DFBN@PVA* films in harsh environments has been achieved, where they can sustain bright RTP emission while immersed in water for five days, marking the longest duration of stable phosphorescence generation observed in underwater RTP materials based on *PVA* matrix. Consequently, post-ISB *DFBN@PVA* films provide opportunities for their potential across a broad spectrum of practical applications, ranging from multicolor secure information systems to underwater phosphorescence and advanced antibacterial technologies.

## Methods

### Materials

Unless otherwise stated, all reagents utilized in the experiments were acquired from commercial suppliers: 1,1’-bi-2-naphthol (*BINOL*, 99%), 1,1’-binaphthyl (98%), *S*−1,1’-binaphthyl-2,2’-dicarboxylic acid (98%), (*S*)-(-)−3-3’-bis(3,5-bis(trifluoromethyl)phenyl)−1,1’-bi-2-naphthol (95%), 2,2’-bis(diphenylphosphino)−1,1’-binaphthalene (97%), carbazole (≥99.9%), 4,4’-di(9H-carbazol-9-yl)−1,1’-biphenyl (98%), 1-(3-dimethylaminopropyl)−3-ethylcarbodiimide hydrochloride (≥99.5%), 4-dimethylaminopyridine (99.0%), N,N-dimethylformamide (≥99,5%), 9-fluorenemethanol (98%), 2-hydroxyterephthalic acid (98%), 4-nitrophthalonitrile (98%), poly(vinyl alcohol) (*PVA*) (molecular weight = 85,000 to 124,000 g/mol, 100% hydrolyzed), potassium carbonate (≥99%), 1-pyrenecarboxylic acid (97%), and tetrachloroterephthalonitrile (99%). Silica gel with 200-300 mesh was used for flash column chromatography. The synthesis of *DFBN*, *DCBN* and *DPCA* was according to the reported literature papers^30,43^.

### Measurements

*BINOL@PVA* was excited at 254 nm, *DPBN@PVA*, *DFBN@PVA*, *DCBN@PVA* and *DPCA@PVA* were irradiated using a 365 nm UV source. The FT-IR spectra were measured by a Nicolet iS-50 Spectrometer. The TA Q50 apparatus was utilized to conduct thermogravimetric analysis (TGA) at a heating rate of 10 °C min^−1^ under a *nitrogen* environment. The TA Q20 instrument was utilized to perform differential scanning calorimetry (DSC) at a heating rate of 10 °C min^−1^ under *nitrogen*. The DRX-400 NMR spectrometer was employed to record ^1^H NMR and ^13^C NMR spectra with TMS as an internal standard. The AB Sciex Triple TOF 5600+ was used for high-resolution mass spectrometry (HRMS) in electrospray ion mode. The transient absorption measurements were detected by the HELIOS Ultrafast Systems (365 nm, 100 fs, 5 mJ per pulse, 1 kHz repetition rate). The Edinburgh FLS 1000 fluorescence spectrophotometer equipped with a xenon lamp (450 W) was used for recording steady-state spectra in which the excitation conditions were continuous. A laser device (280 nm, 365 nm) was employed for *τ*_F_ measurements. A microsecond flash-lamp (100 W) was used to characterize the time-gated spectra and *τ*_P_, TRES and temperature-dependent time-gated spectra. An integrating sphere equipped was used to calculate the absolute photoluminescence quantum yield (*Φ*_PL_) with the xenon lamp excitation and the absolute phosphorescence quantum yield (*Φ*_P_) with a microsecond flash lamp excitation. The fluorescence quantum yield (*Φ*_F_) was obtained by subtracting *Φ*_P_ from *Φ*_PL_. All spectra acquired under microsecond flash lamp excitation were collected with a 2 ms delay and an 8 ms gate. The Rigaku D/Max-2500 was used to record powder *X*-ray diffraction (XRD) using Cu K_α_ radiation with a 2θ range of 5–60°, at 40 kV and 30 mA, and a scanning rate of 0.01° s^−1^ at room temperature. The XPS measurements were performed on an AXIS Ultra DLD (Kratos, USA) using a monochromatic Al K_α_ X-ray source (anode HT = 15 kV) operated at a vacuum better than 10^−7^ Pa. Inductively coupled plasma-mass spectrometry (ICP-MS) from Thermol Fisher Scientific was used. The JSM-6700F (JEOL) was used to obtain scanning electron microscope (SEM) images. The Canon EOS 80D camera was used to record photos and supporting videos.

### Synthesis of *DPBN*

A reaction involving 1,1’-bi-2-naphthol (*BINOL*, 2.9 g, 10 mmol), 4-nitrophthalonitrile (3.8 g, 22 mmol), and potassium carbonate (4.1 g, 30 mmol) was conducted under *nitrogen* in dried DMF (30 mL) at ambient temperature for 20 h. Subsequently, the resultant mixture was quenched by the cold dilute hydrochloric acid (2 N, 500 mL). The precipitated solid was subsequently isolated and washed with distilled *water* until the effluent exhibited neutrality. Furthermore, the crude product was purified via a double recrystallization process from a 1:1 mixture of *methanol* and *acetonitrile*, eventually yielding a high-purity white powder product (4.3 g, 79%). ^1^H NMR (400 MHz, DMSO-*d*_6_) δ 8.19 (d, J = 9.0 Hz, 1H), 8.07 (d, J = 8.2 Hz, 1H), 7.85 (d, J = 8.7 Hz, 1H), 7.57-7.44 (m, 3H), 7.38 (t, J = 7.7 Hz, 1H), 7.17 (dt, J = 8.7, 2.4 Hz, 2H). ^13^C NMR (101 MHz, DMSO-*d*_6_) δ 160.86, 149.78, 136.25, 133.67, 131.87, 131.56, 128.94, 127.91, 126.46, 125.82, 122.72, 122.67, 122.29, 120.41, 116.70, 116.06, 115.48, 108.34. HRMS (m/z): [M]^+^ calcd. for C_36_H_18_N_4_O_2_, 539.1463; found: 539.1498.

### Synthesis of *DFBN*

In a 50 mL flask, dry *DMF* (25 mL) was introduced to dissolve tetrafluoroterephthalonitrile (5 mmol, 1 g), potassium carbonate (1.4 g, 10 mmol) and *BINOL* (1.4 g, 5 mmol). The resulting mixture was stirred at room temperature under a *nitrogen* atmosphere for 12 h. After the reaction, the residue was carefully poured into water and extracted with ethyl acetate. The combined organic layers were purified by column chromatography (*ethyl acetate*/*n-hexane* = 1:5) to afford a light-yellow powder (1.9 g, 87% yield). ^1^H NMR (400 MHz, DMSO-*d*_6_) δ 8.35–8.30 (m, 1H), 8.18 (dt, J = 8.3, 0.9 Hz, 1H), 7.68-7.62 (m, 2H), 7.54 (ddd, J = 8.3, 6.8, 1.3 Hz, 1H), 7.39 (dd, J = 8.5, 1.1 Hz, 1H). ^13^C NMR (101 MHz, DMSO-*d*_6_) δ 149.14, 146.37, 132.64, 132.42, 131.63, 129.27, 128.41, 127.15, 126.32, 124.55, 120.84, 109.92, 103.07. HRMS (m/z): [M]^+^ calcd. for C_28_H_12_F_2_N_2_O_2_, 446.0867; found, 446.1206.

### Synthesis of *DCBN*

A mixture of *DFBN* (1 g, 2.2 mmol), carbazole (0.8 g, 5 mmol), and *potassium carbonate* (1.5 g, 11 mmol) was dissolved in dry *DMF* (25 mL). The resultant reaction mixture was stirred at ambient temperature for 2.5 h. Subsequently, the reaction was quenched by the addition of *water* and extracted with *dichloromethane* (*CH*_*2*_*Cl*_*2*_). The combined organic layers were purified via column chromatography using a *hexane*/*ethyl acetate* (8:1) eluent, ultimately yielding *DCBN* as a yellow solid (1.3 g, 81%). ^1^H NMR (400 MHz, DMSO-*d*_6_) δ 8.45–8.40 (m, 1H), 8.24 (d, J = 8.4 Hz, 1H), 7.90–7.85 (m, 2H), 7.77 (d, J = 8.8 Hz, 1H), 7.68 (ddd, J = 8.1, 6.8, 1.2 Hz, 1H), 7.61–7.54 (m, 2H), 7.48–7.42 (m, 2H), 7.17 (ddd, J = 8.3, 7.2, 1.3 Hz, 1H), 7.12–7.04 (m, 3H). ^13^C NMR (101 MHz, DMSO-*d*_6_) δ 151.91, 149.33, 139.89, 139.73, 136.14, 132.51, 131.75, 129.28, 128.36, 127.17, 126.46, 126.01, 125.93, 124.60, 123.36, 123.30, 121.65, 121.28, 120.60, 120.54, 114.75, 112.48, 111.39, 111.21. HRMS (m/z): [M]^+^ calcd. for C_52_H_28_N_4_O_2_, 741.2246; found, 741.2290.

### Synthesis of *DPCA*

A solution containing *BINOL* (1 g, 3.5 mmol), *4-dimethylaminopyridine* (*DMAP*, 1.2 g, 9.8 mmol), *pyrene-1-carboxylic acid* (1.9 g, 7.7 mmol), and *1-(3-dimethylaminopropyl)−3-ethylcarbodiimide hydrochloride* (*EDC·HCl*, 2.2 g, 11.5 mmol) was stirred at 40 °C for 22 h to facilitate the coupling reaction under N_2_ atmosphere. Upon completion of the reaction, the mixture was quenched with water and extracted with *CHCl₃* twice. The combined organic layers were washed with water and dried over *sodium sulfate* (*Na₂SO₄*) effectively. The crude product was further purified by column chromatography using a *hexane*/*dichloromethane* (6:1) eluent system, yielding the final product as a yellow powder (727.4 mg, 28%). ^1^H NMR (400 MHz, CDCl_3_) δ 8.87 (d, J = 9.4 Hz, 1H), 8.22–8.17 (m, 2H), 8.14–8.05 (m, 3H), 8.04–7.96 (m, 4H), 7.92 (d, J = 8.2 Hz, 1H), 7.75 (d, J = 8.9 Hz, 1H), 7.60 (dd, J = 8.3, 1.2 Hz, 1H), 7.49 (ddd, J = 8.2, 6.8, 1.3 Hz, 1H), 7.42 (ddd, J = 8.2, 6.8, 1.4 Hz, 1H). ^13^C NMR (101 MHz, CDCl_3_) δ 165.94, 147.52, 134.51, 133.69, 131.78, 131.42, 130.87, 130.25, 129.74, 129.35, 128.72, 128.12, 127.08, 127.01, 126.37, 126.26, 126.21, 125.88, 124.71, 124.58, 124.23, 124.03, 122.35, 122.22. HRMS (m/z): [M]^+^ calcd. for C_50_H_30_O_4_, 742.2144; found, 742.1969.

### Preparation of *BINs@PVA* films and ISB treatment process

The *BINs* were dissolved in a *PVA-DMSO* solution, with the *PVA* concentration in *DMSO* set at 45 mg mL^−1^, and the resulting *BINs* solution was poured into a PTFE mold (100 mm × 80 mm × 5 mm). The solvent was then evaporated in an oven with forced air circulation at 85 °C, thereby producing the *BINs@PVA* films. Upon detachment of the films from the mold, it was removed from the oven, following which it was allowed to undergo cooling to room temperature. The cooled films were subsequently cut into small squares (50 mm × 40 mm) and immersed in deionized water at 35 °C for at least 30 min to ensure complete swelling equilibrium. Following this, the *BINs@PVA* films were soaked in 100 mL iodine solution (*I*_*2*_: 0.5 g; *KI*: 0.25 g) for 10, 20, 30 and 40 s (denoted as I_10_, I_20_, I_30_, and I_40_), after which they were stretched to twice their original length using a simple stretching device and held for 2 min (denoted as S_y_). Upon completion of the stretching process, the *BINs@PVA* films were subjected to a post-treatment procedure in anhydrous ethanol for 5 min, after which they were crosslinked by immersion in a 4 wt% *boric acid* (*BA*) solution for several minutes (denoted as B_z_). Subsequently, the BA on the surface of the film was removed by washing with deionized water, after which the film was placed in an oven at 60 °C overnight. For convenience, the aforementioned parameters are defined as I_x_S_y_B_z_.

### Antibacterial performance

An agar medium, composed of peptone (10 g), beef extract (10 g), NaCl (5 g) and agar (15 g), with the pH regulated within the 7.2–7.5 range, underwent sterilization at a pressure of 15 lbs for a controlled duration of 30 min. After sterilization, the medium was transferred into a sterile petri dish to enable solidification. A suspension of Staphylococcus aureus or Escherichia coli, precisely calibrated to a concentration range of 10^−5^−10^−6^ cfu mL^−1^, was then evenly dispensed across the agar plate at a volume of 0.1 mL and permitted to dry naturally. Throughout these procedures, aseptic conditions were rigorously maintained to preclude any potential contamination. The *DFBN@PVA* sample, with exacting precision and care, was positioned on the agar plate within a culture room thermostatically controlled at 37 °C for a period of 12 h.

### Absolute photoluminescence, phosphorescence, and fluorescence quantum yields

The absolute *Φ*_PL_ was measured using an integrating sphere equipped in the FLS1000 spectrometer. In this method, the number of absorbed excitation photons was determined by comparing the excitation light intensity with and without the sample inside the integrating sphere. Therefore, the calibration of excitation pulse intensity was inherently accounted for, and no external calibration was required. The calculations were defined by the following Eq. (1).

1 $${{\varPhi }}_{{{\rm{PL}}}}=\frac{{{L}}_{{{\rm{sam}}}}}{{{E}}_{{{\rm{ref}}}}-{{E}}_{{{\rm{sam}}}}}$$

where *E*_ref_ represents the excitation light intensity measured in the absence of the sample, *E*_sam_ represents the excitation light intensity measured in the presence of the sample (i.e., the unabsorbed excitation light), *L*_sam_ represents the emission intensity of the sample, and the denominator (*E*_ref_-*E*_sam_) denotes the absorbed excitation photons. This approach is widely recognized as a reliable method for determining absolute quantum yields.

The time-gating technique was employed only to separate the phosphorescence component from the total emission. Specifically, the time-gated spectrum was collected with a 2 ms delay and an 8 ms gate under the microsecond flash-lamp excitation to isolate phosphorescence, while the absolute phosphorescence quantum yield was still determined based on the integrating sphere method. According to Supplementary Fig. 23, the delayed fluorescence was excluded in *BINs@PVA*. Therefore, the sum of$\,{\varPhi }$_F_ and *Φ*_P_ equaled the total photoluminescence quantum yield (*Φ*_PL_).

### Kinetic calculations and photophysical rate constants

Based on the measured *Φ*_F_, *Φ*_P_, *τ*_F_, and *τ*_P_ data, the radiative and nonradiative decay rates as well as the ISC rate can be calculated according to standard photophysical dynamic equations^44,45^. Theoretically, the *τ*_F_, *Φ*_F_, *τ*_P_, *Φ*_P_ and *Φ*_ISC_ can be expressed in Eqs. (2–7), respectively.

2 $${\it\tau }_{{{\rm{F}}}}=\frac{1}{{k}_{{{\rm{r}}}}^{{{\rm{F}}}}+{k}_{{{\rm{nr}}}}^{{{\rm{F}}}}+{k}_{{{\rm{ISC}}}}}$$

3 $${\it\tau }_{{{\rm{P}}}}=\frac{1}{{k}_{{{\rm{r}}}}^{{{\rm{P}}}}+{k}_{{{\rm{nr}}}}^{{{\rm{P}}}}}$$

4 $$ {\varPhi }_{{{\rm{F}}}}={\varPhi }_{{{\rm{PL}}}}{\varPhi }_{{{\rm{P}}}}=\frac{{k}_{{{\rm{r}}}}^{{{\rm{F}}}}}{{k}_{{{\rm{r}}}}^{{{\rm{F}}}}+{k}_{{{\rm{nr}}}}^{{{\rm{F}}}}+{k}_{{{\rm{ISC}}}}}={k}_{{{\rm{r}}}}^{{{\rm{F}}}}\times {\it\tau }_{{{\rm{F}}}}$$

5 $${\varPhi }_{{{\rm{ISC}}}}=\frac{{k}_{{{\rm{ISC}}}}}{{k}_{{{\rm{r}}}}^{{{\rm{F}}}}+{k}_{{{\rm{nr}}}}^{{{\rm{F}}}}+{k}_{{{\rm{ISC}}}}}={k}_{{{\rm{ISC}}}}\times {\it\tau }_{{{\rm{F}}}}$$

6 $${\varPhi }_{{{\rm{P}}}}={\varPhi }_{{{\rm{ISC}}}}\times \frac{{k}_{{{\rm{r}}}}^{{{\rm{P}}}}}{{k}_{{{\rm{r}}}}^{{{\rm{P}}}}+{k}_{{{\rm{nr}}}}^{{{\rm{P}}}}}={\varPhi }_{{{\rm{ISC}}}}\times {k}_{{{\rm{r}}}}^{{{\rm{P}}}}\times {\it\tau }_{{{\rm{P}}}}$$

7 $${\varPhi }_{{{\rm{ISC}}}}=1-{\varPhi }_{{{{\rm{F}}}}}{\varPhi }_{{{\rm{IC}}}}$$

where ${k}_{{{\rm{r}}}}^{{{\rm{F}}}}$ and ${k}_{{{\rm{nr}}}}^{{{\rm{F}}}}$ represent the radiative and nonradiative rate constant of fluorescence respectively, *k*_ISC_ and *Φ*_ISC_ are the rate constant and QY of ISC respectively, and ${k}_{{{\rm{r}}}}^{{{\rm{P}}}}$ and ${k}_{{{\rm{nr}}}}^{{{\rm{P}}}}$ are the radiative and nonradiative rate constant of RTP respectively. Generally, the internal conversion (IC) between S_1_ and S_0_ is negligible^46–48^ when their energy gap exceeds 2 eV. Since the energy gap between S_1_ and S_0_ is calculated using the equation ΔE_S1→S0_ = 1240/λ, this implies that if the fluorescence emission wavelength is less than 620 nm, the excited singlet state predominantly relaxes via fluorescence emission and ISC. In this work, *BINs@PVA* films satisfy this assumption according to Supplementary Fig. 18. Combined with the above equations, ${k}_{{{\rm{r}}}}^{{{\rm{F}}}}$, *k*_ISC_ and *Φ*_ISC_ can be obtained by the following Eqs. (8–10).

8 $${\it{k}}_{{{\rm{P}}}}=\frac{{\varPhi }_{{{\rm{F}}}}}{{\it\tau }_{{{\rm{F}}}}}$$

9 $${\varPhi }_{{{\rm{ISC}}}}=1-{\varPhi }_{{{\rm{F}}}}$$

10 $${\it{k}}_{{{\rm{ISC}}}}=\frac{1-{\varPhi }_{{{\rm{F}}}}}{{\it\tau }_{{{\rm{F}}}}}$$

With the Eqs. (2), (4) and (5), *k*_ISC_ and *k*^F^_nr_ can be expressed in Eqs. (10) and (11).

11 $${\it{k}}_{{{\rm{nr}}}}^{{{\rm{F}}}}=\frac{1}{{\it{\tau }}_{{{{\rm{F}}}}}}-{{\it{{\rm{k}}}}}_{{{\rm{r}}}}^{{{\rm{P}}}}-{k}_{{{\rm{ISC}}}}=\frac{1-{\it{\varPhi }}_{{{\rm{F}}}}-{\it{\varPhi }}_{{{\rm{ISC}}}}}{{\it{\tau }}_{{{{\rm{F}}}}}}$$

Thus, with the Eqs. (3) and (6), *k*^P^_r_ and *k*^P^_nr_ can be expressed in Eqs. (12) and (13).

12 $${{\it{\rm{k}}}}_{{{\rm{r}}}}^{{{\rm{P}}}}=\frac{{\varPhi }_{{{\rm{P}}}}}{{\varPhi }_{{{\rm{ISC}}}}\times {\it\tau }_{{{\rm{P}}}}}$$

13 $${k}_{{{\rm{nr}}}}^{{{\rm{P}}}}=\frac{1}{{\it\tau }_{{{\rm{P}}}}}-{k}_{{{\rm{r}}}}^{{{\rm{P}}}}$$

### Theoretical calculation methods

The geometry structures of molecules *DPBN*, *DFBN*, *DCBN*, and *DPCA* (Supplementary Data 1) before and after ISB were optimized by dispersion corrected density functional theory (DFT-D3) at the B3LYP-D3/def2-SVP level^49,50^. The transition energy and spin-orbit coupling constants (SOC) of S_1_ and T_1_ states were calculated at M062X/def2-TZVP level for *BINs* and M062X/DKH-def2-TZVP level with the second-order Douglas-Kroll-Hess (DKH2) Hamiltonian for composites by the time-dependent density functional theory (TD-DFT) method^51–53^. The energy of intermolecular interactions between *BINs* and *KI*_*3*_*@PVA* substrates was calculated at the B3LYP-D3/def2-TZVP level by the Eq. (14), which was considered on the basis set superposition error (BSSE) correction^54^.

14 $$\varDelta E={E}_{{{{\rm{Phosphor@}}}}{{{\rm{matrix}}}}}{-E}_{{{{\rm{Phosphor}}}}}{-E}_{{{{\rm{m}}}}{{{\rm{atrix}}}}}{-E}_{{{{\rm{B}}}}{{{\rm{SSE}}}}}$$

All the calculation processes were supported by the ORCA program package^55,56^. The electronic excitation characters of S_1_ and T_1_ states were performed by hole-electron analysis using the Multiwfn 3.8(dev) code^57,58^. The molecular orbitals, an independent gradient model based on Hirshfeld partition (IGMH) maps^59^ and visual study of interactions in chemical systems, were rendered by means of Visual Molecular Dynamics (VMD)^60^ software based on the wavefunction files exported by Multiwfn.

## Supplementary information

Supplementary Information

Description of Additional Supplementary Files

Supplementary Data 1

Supplementary Movie 1

Supplementary Movie 2

Transparent Peer Review file

## Data Availability

All the data supporting the findings of this study are available within the article and its supplementary information files and from the corresponding author upon request.
