# Supplementary material for: Coordinatively improving polymeric phosphorescence lifetime and quantum yield via triplet exciton modulation
Source: Nat Commun. 2026 Jun 11;17:8890. doi: 10.1038/s41467-026-74266-8 (PMC13500660; doi:10.1038/s41467-026-74266-8)
Supplement: Supplementary file 1 [file 41467_2026_74266_MOESM1_ESM.pdf]

Supplementary Information

Coordinatively Improving Polymeric Phosphorescence Lifetime and Quantum Yield via Triplet Exciton Modulation

Hou et al.

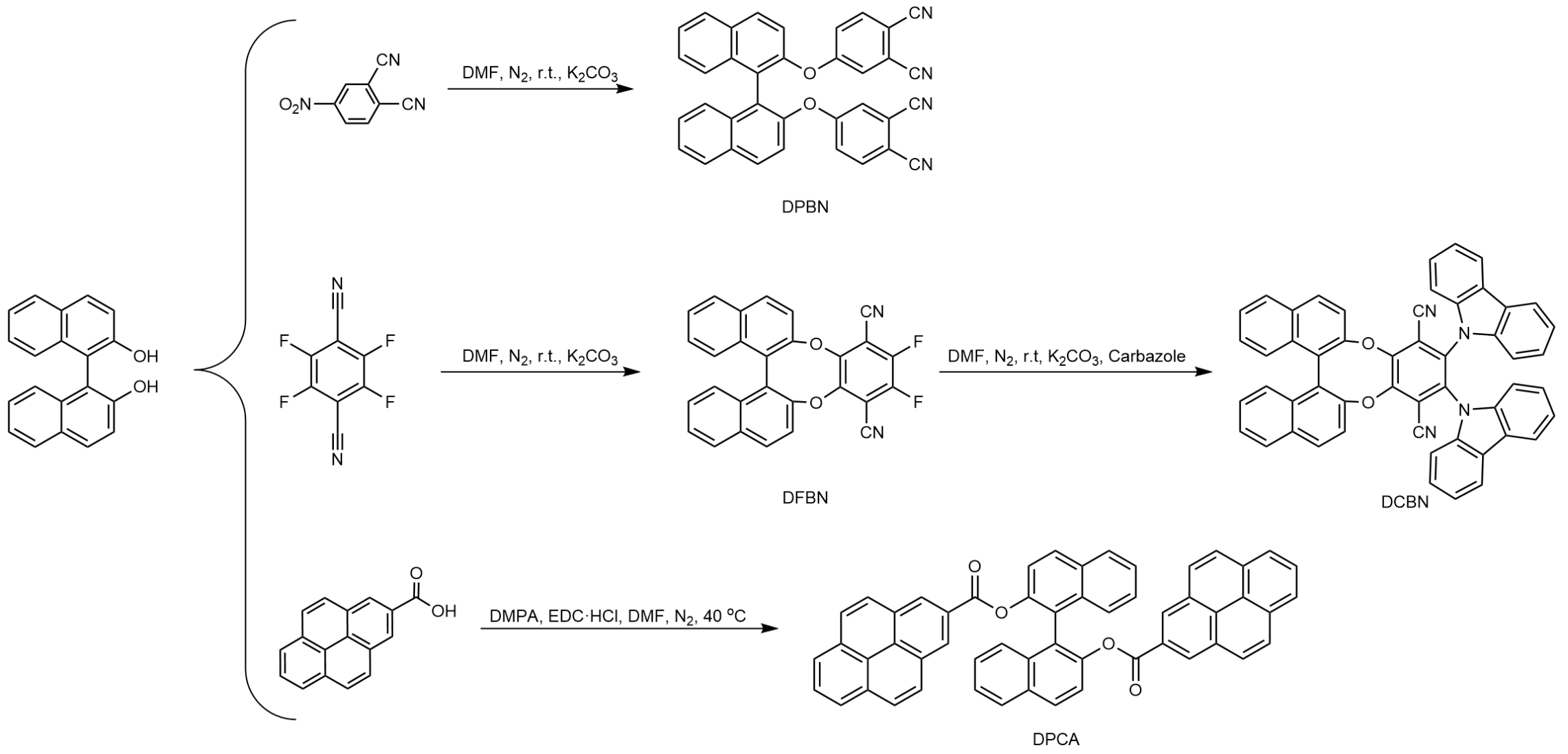

Supplementary Figure 1. Synthetic routes of *BINs*.

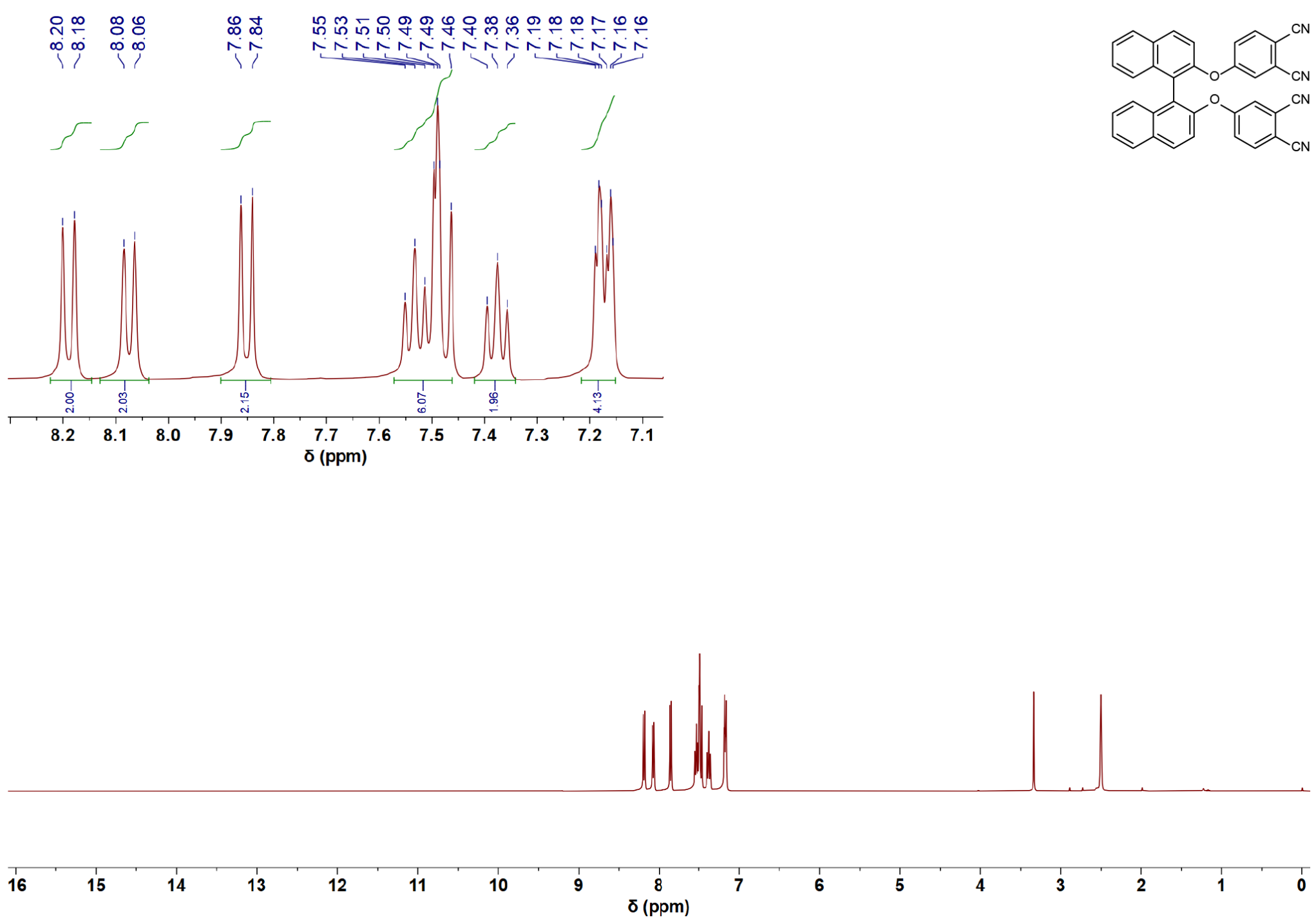

Supplementary Figure 2. ^{1}H NMR (400 MHz) spectrum of *DPBN* molecule in DMSO-d_6.

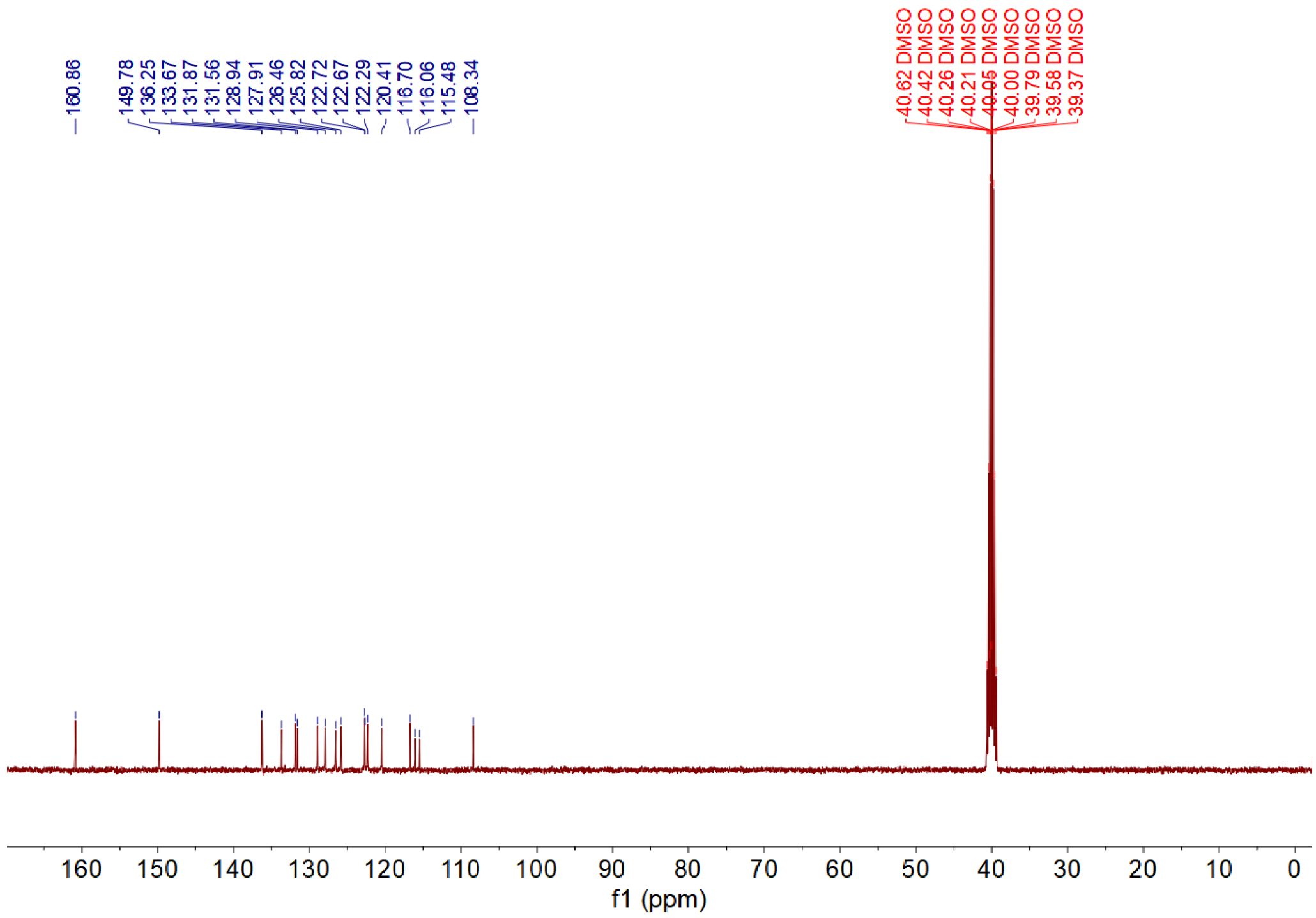

Supplementary Figure 3. ^{13}C NMR (101 MHz) spectrum of *DPBN* molecule in DMSO-d_6.

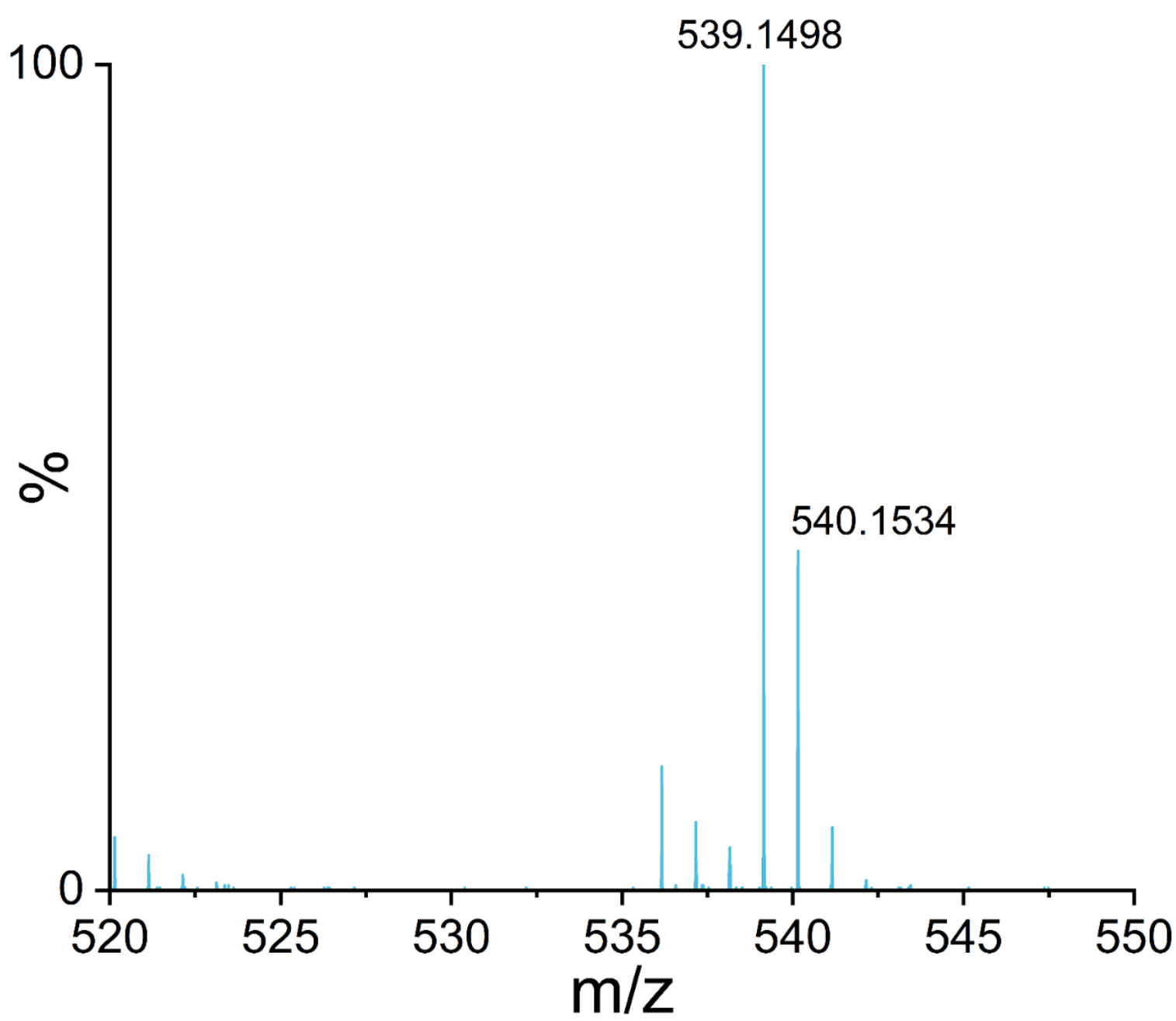

Supplementary Figure 4. HRMS of *DPBN* molecule.

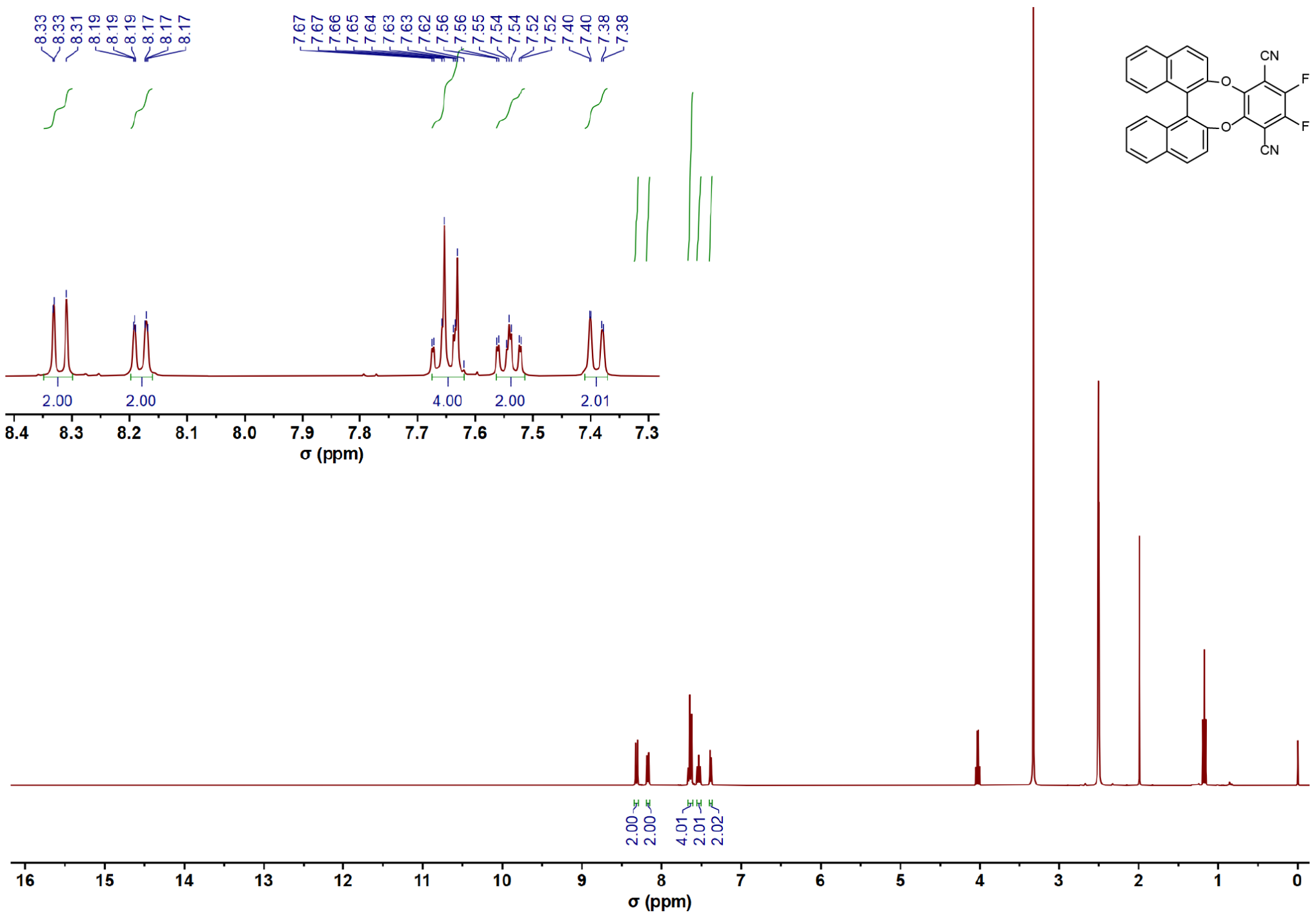

Supplementary Figure 5. ^{1}H NMR spectrum (400 MHz) of *DFBN* molecule in DMSO-d_6.

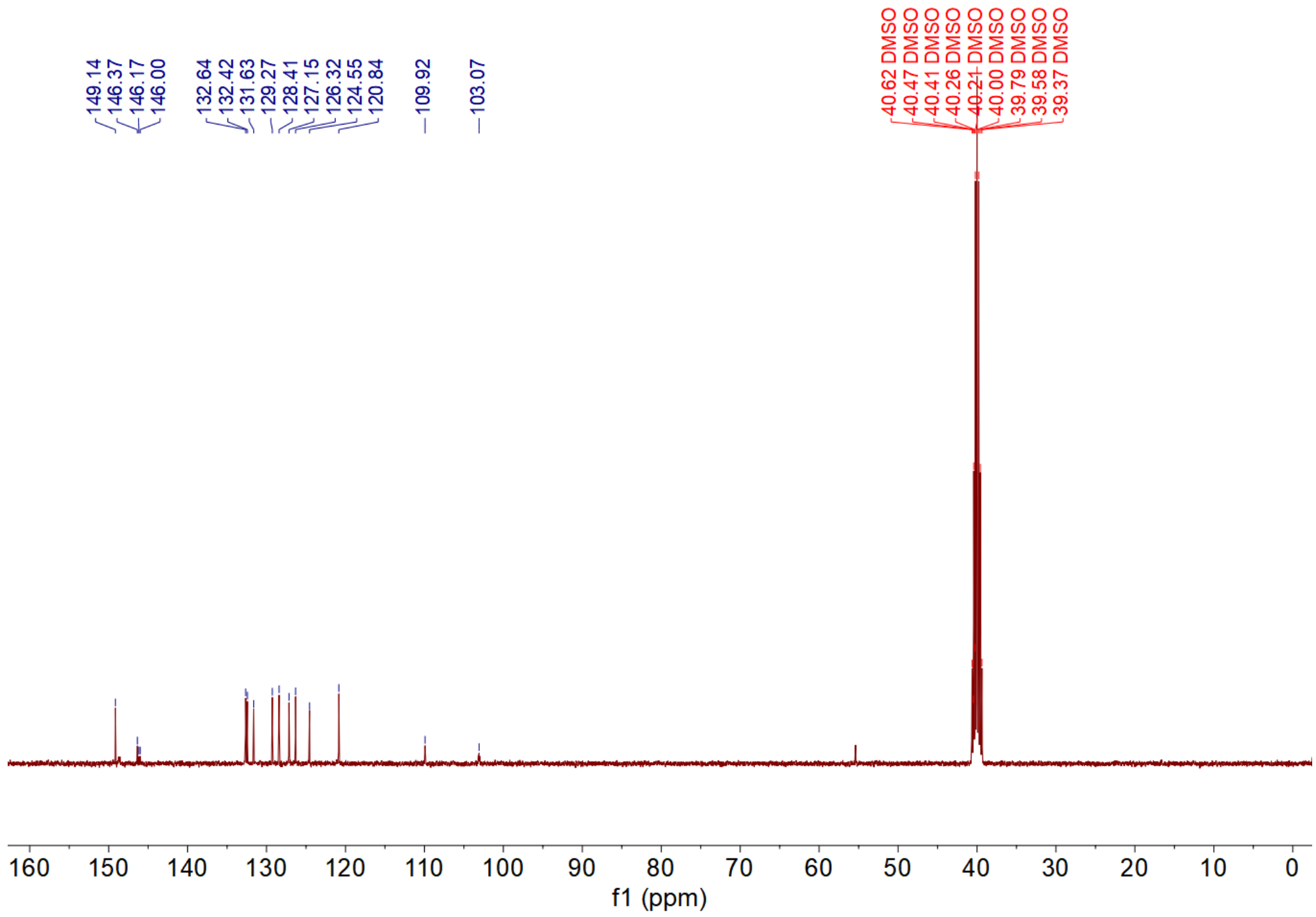

Supplementary Figure 6. ^{13}C NMR (101 MHz) spectrum of *DFBN* molecule in DMSO-d_6.

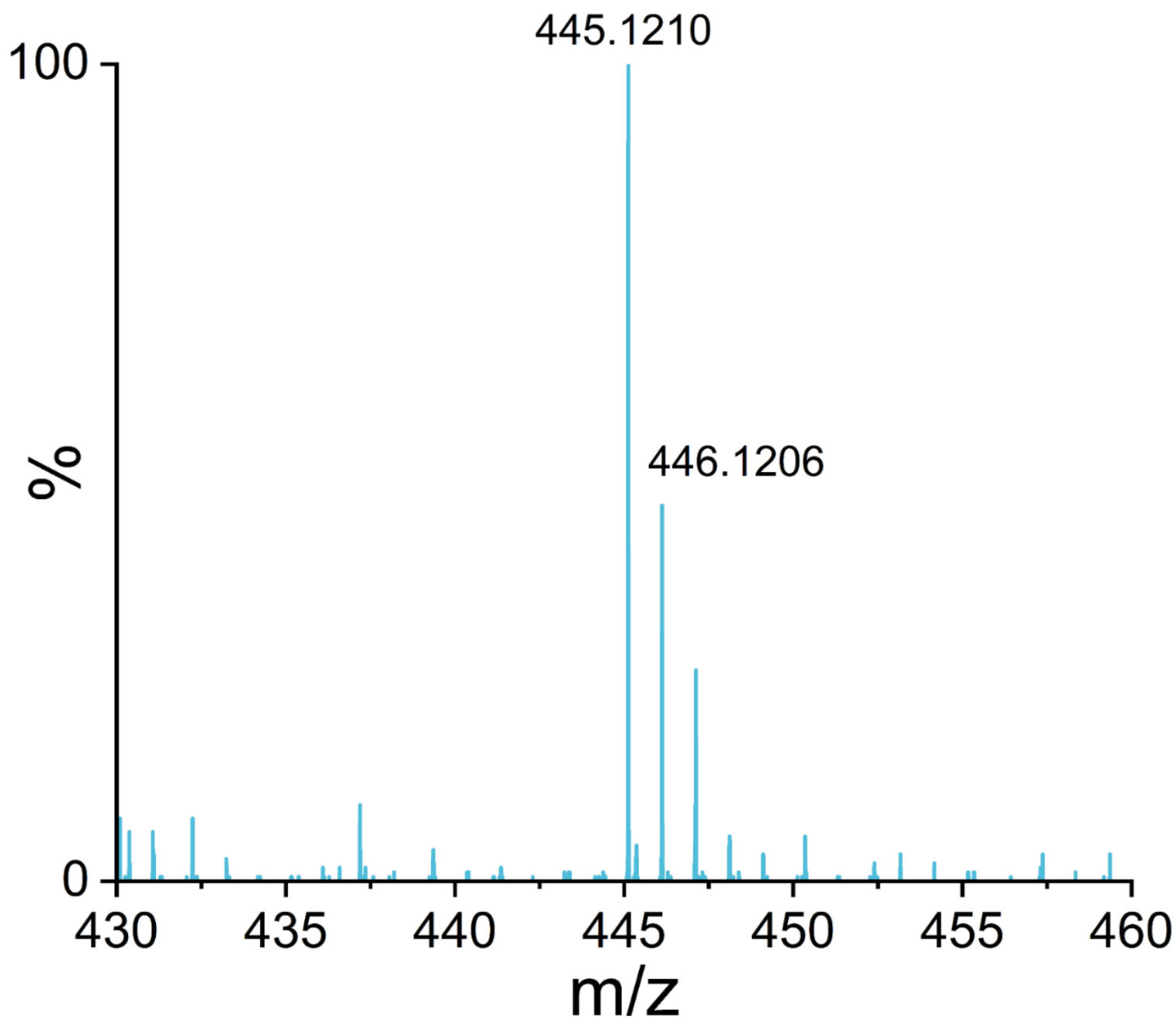

Supplementary Figure 7. HRMS of *DFBN* molecule.

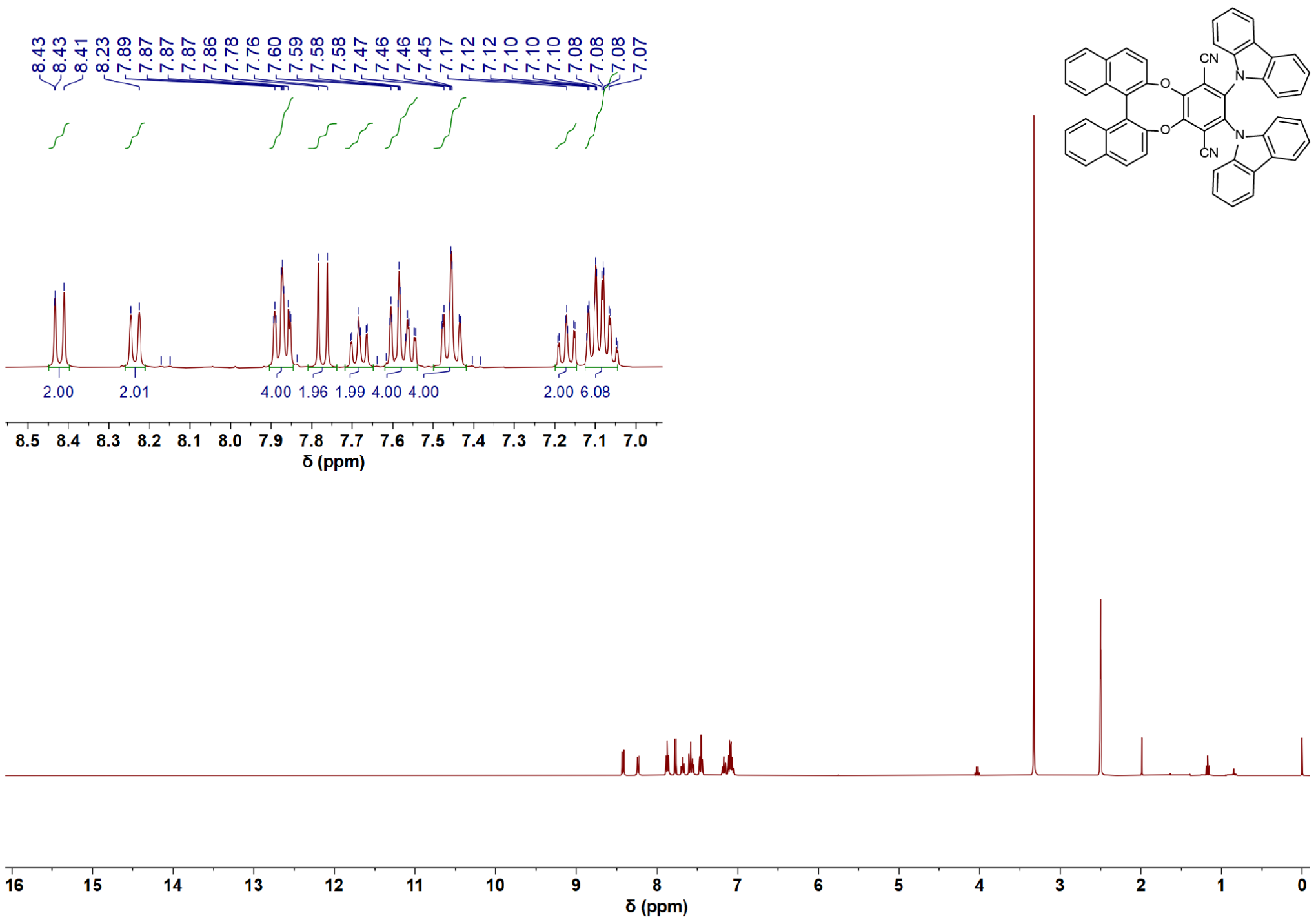

Supplementary Figure 8. ^{1}H NMR spectrum (400 MHz) of *DCBN* molecule in DMSO-d_6.

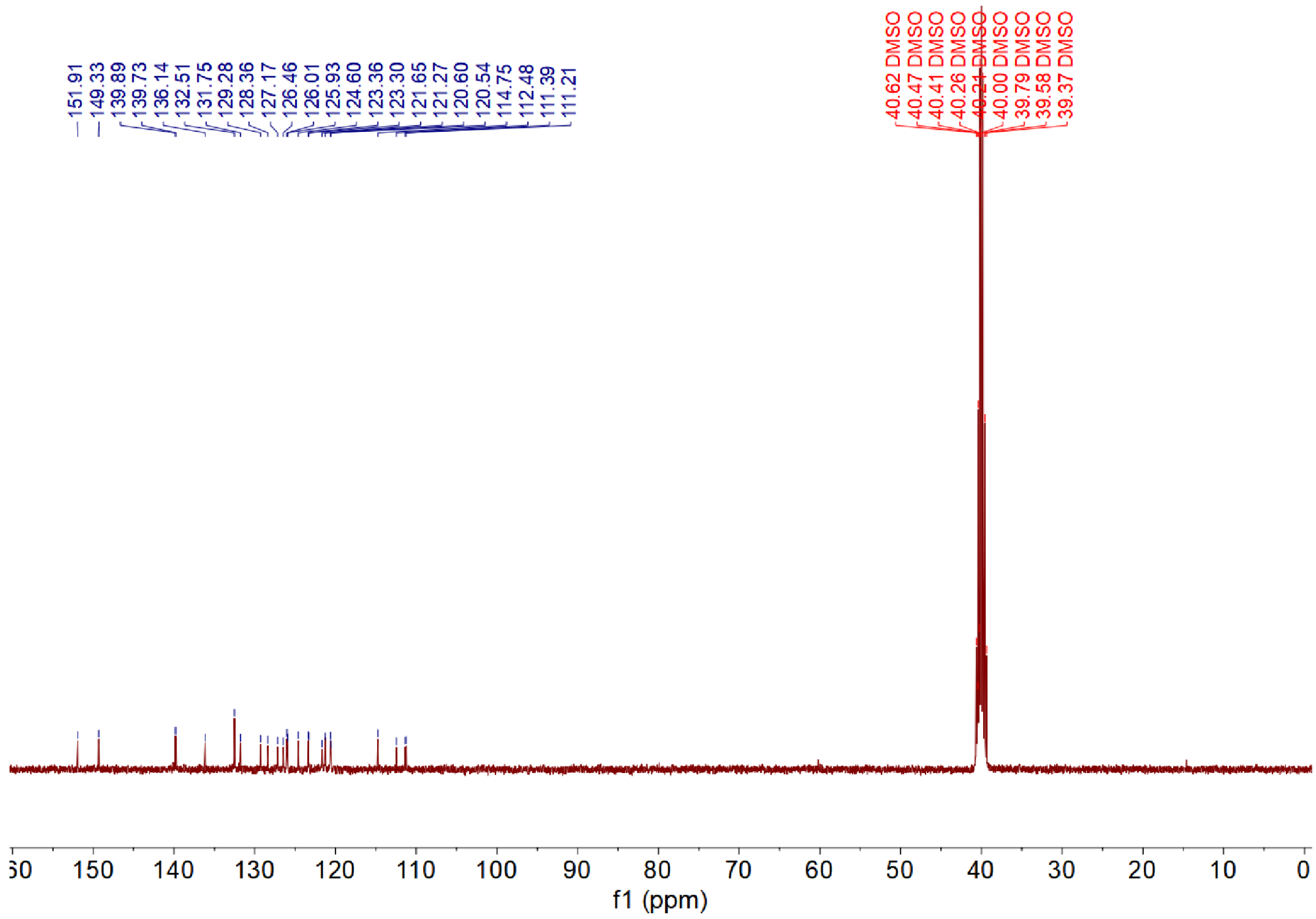

Supplementary Figure 9. ^{13}C NMR (101 MHz) spectrum of *DCBN* molecule in DMSO-d_6.

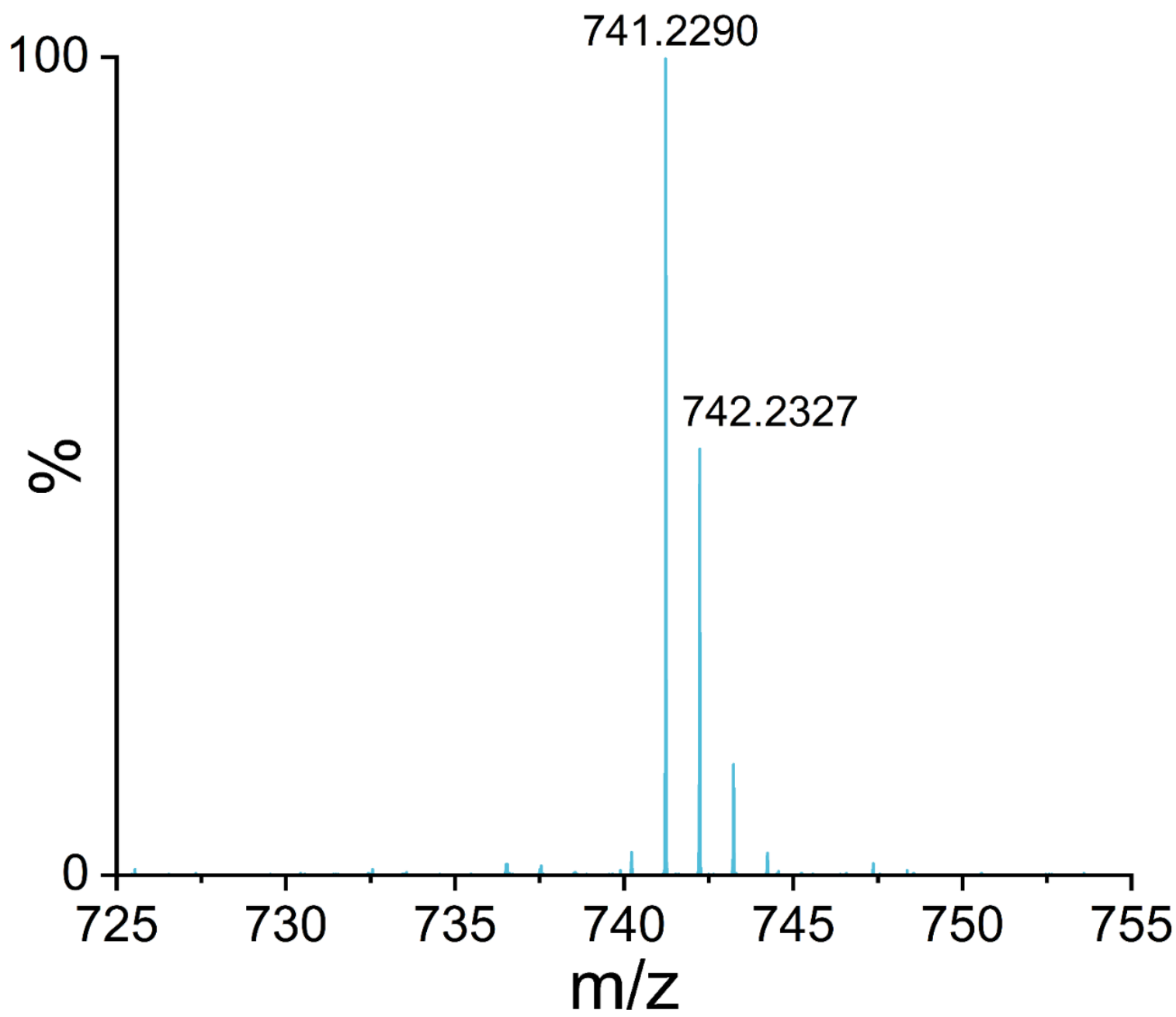

Supplementary Figure 10. HRMS of *DCBN* molecule.

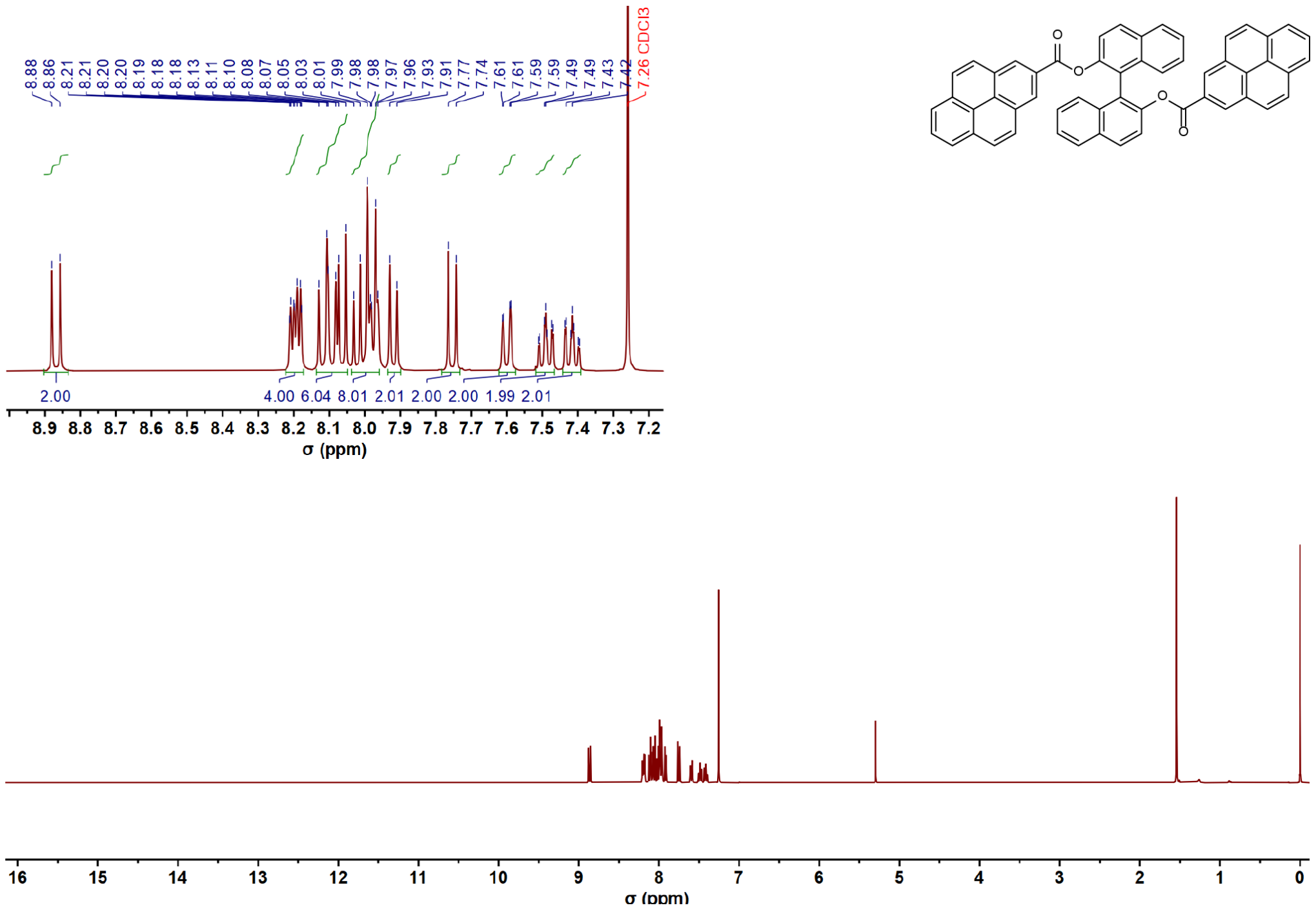

Supplementary Figure 11. ^{1}H NMR (400 MHz) spectrum of *DPCA* molecule in $CDCl_3$.

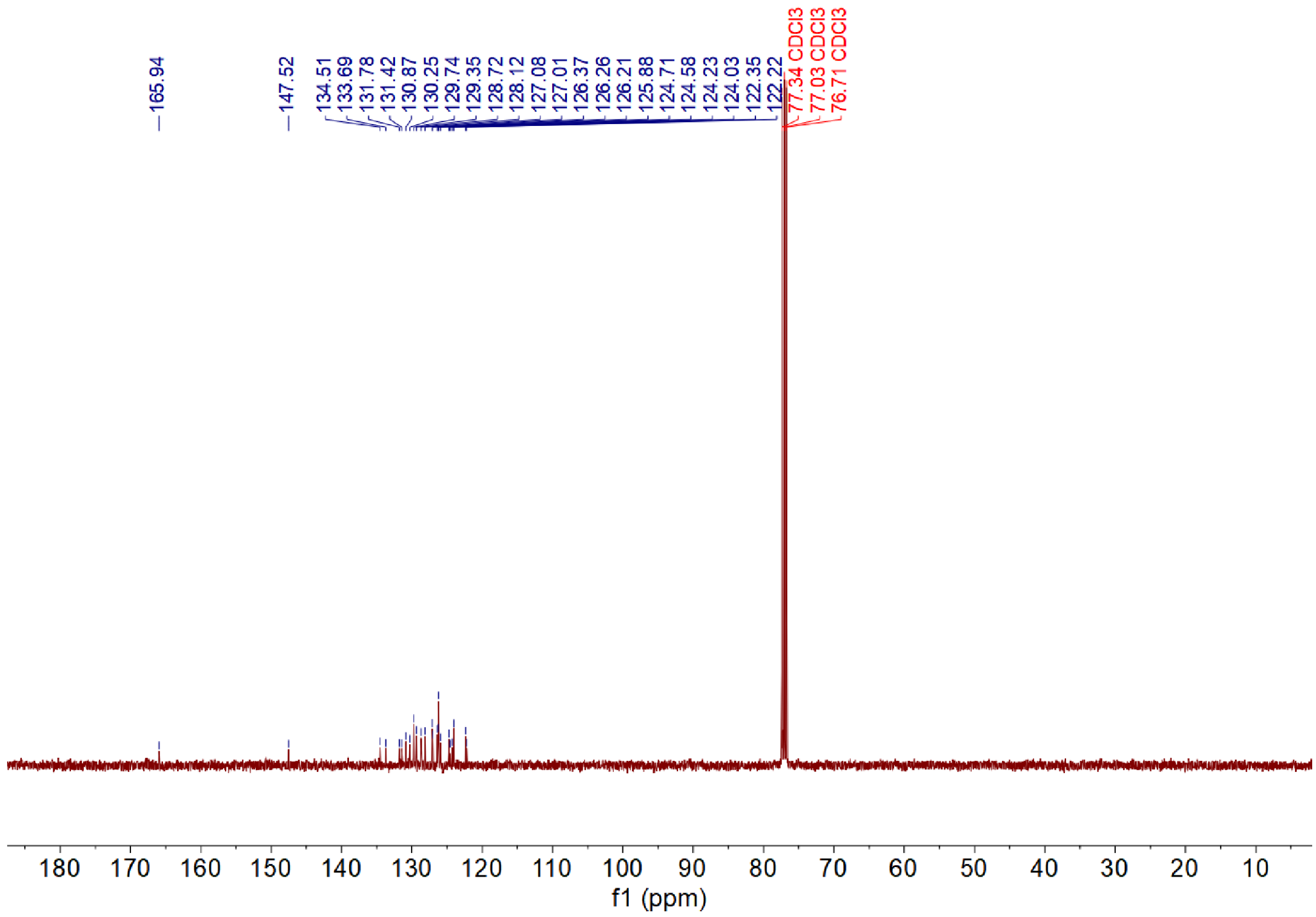

Supplementary Figure 12. ^{13}C NMR (101 MHz) spectrum of *DPCA* molecule in $CDCl_3$.

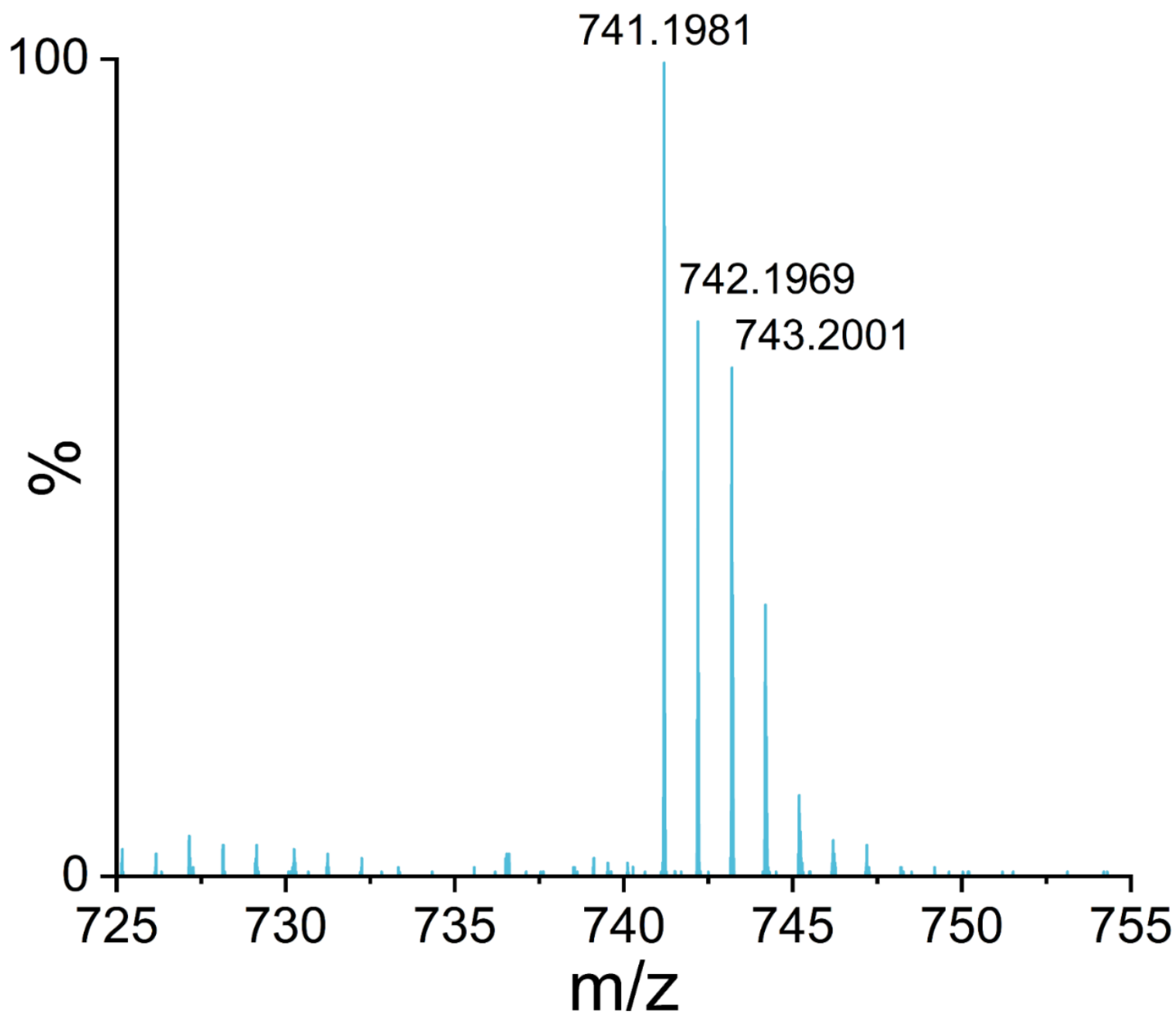

Supplementary Figure 13. HRMS of *DPCA* molecule.

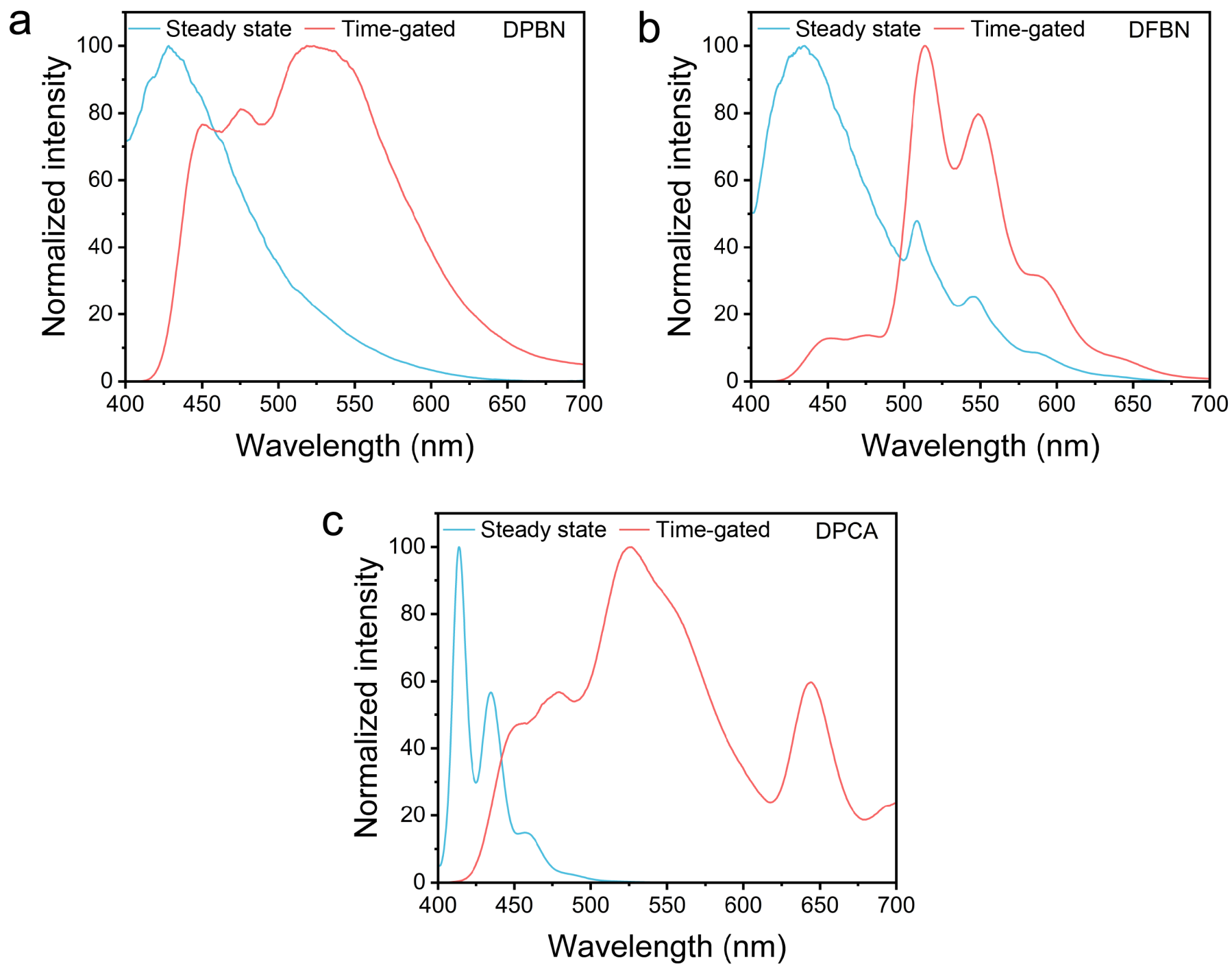

Supplementary Figure 14. Steady-state PL spectra and time-gated spectra. (**a**) *DPBN*, (**b**) *DFBN*, and (**c**) *DPCA* in 2-methyltetrahydrofuran (10 μM) recorded at 77 K by using an F-4700 fluorescence spectrophotometer.

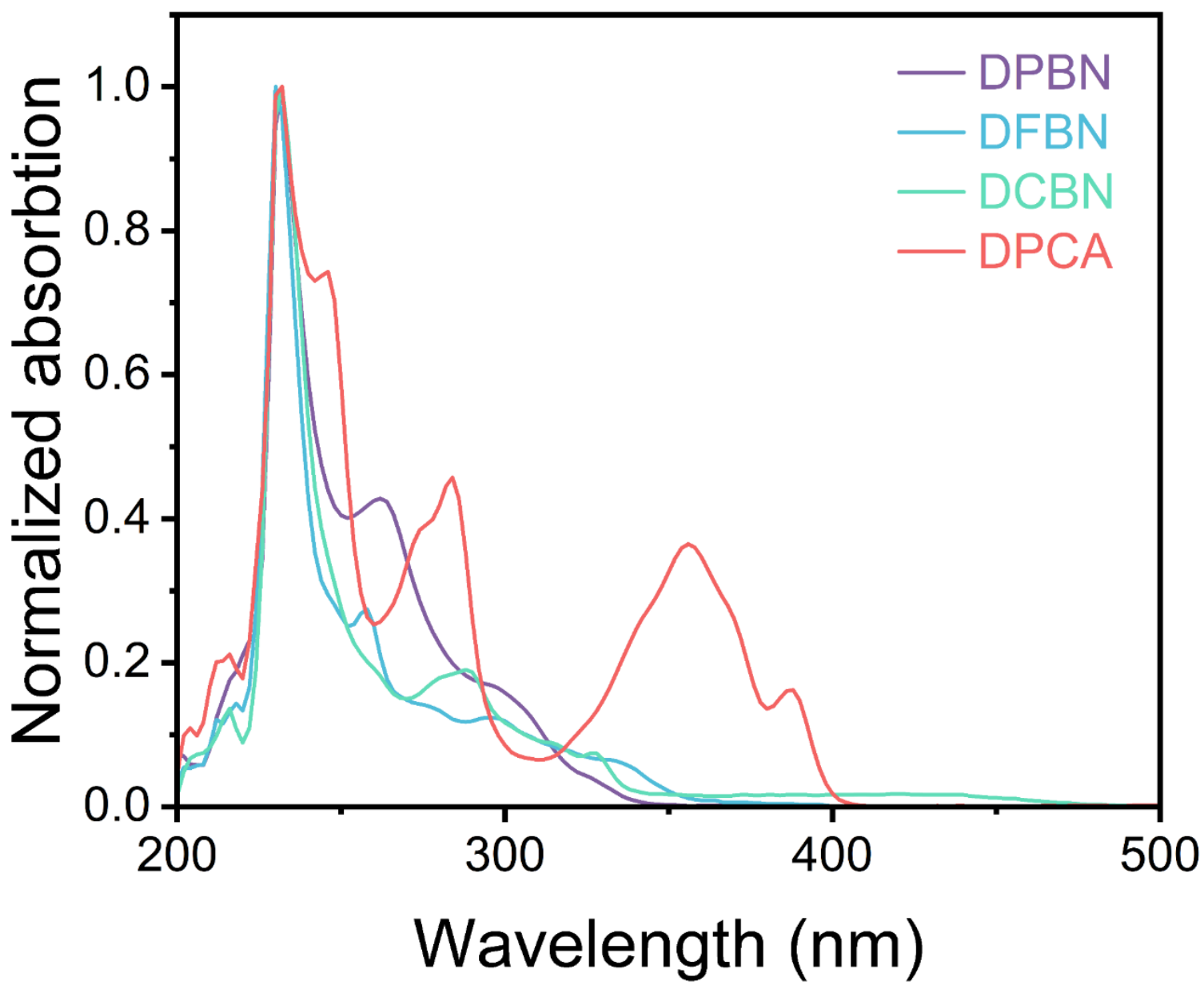

Supplementary Figure 15. UV-vis absorption spectra of *BINs* in CH_2Cl_2 (10 μM).

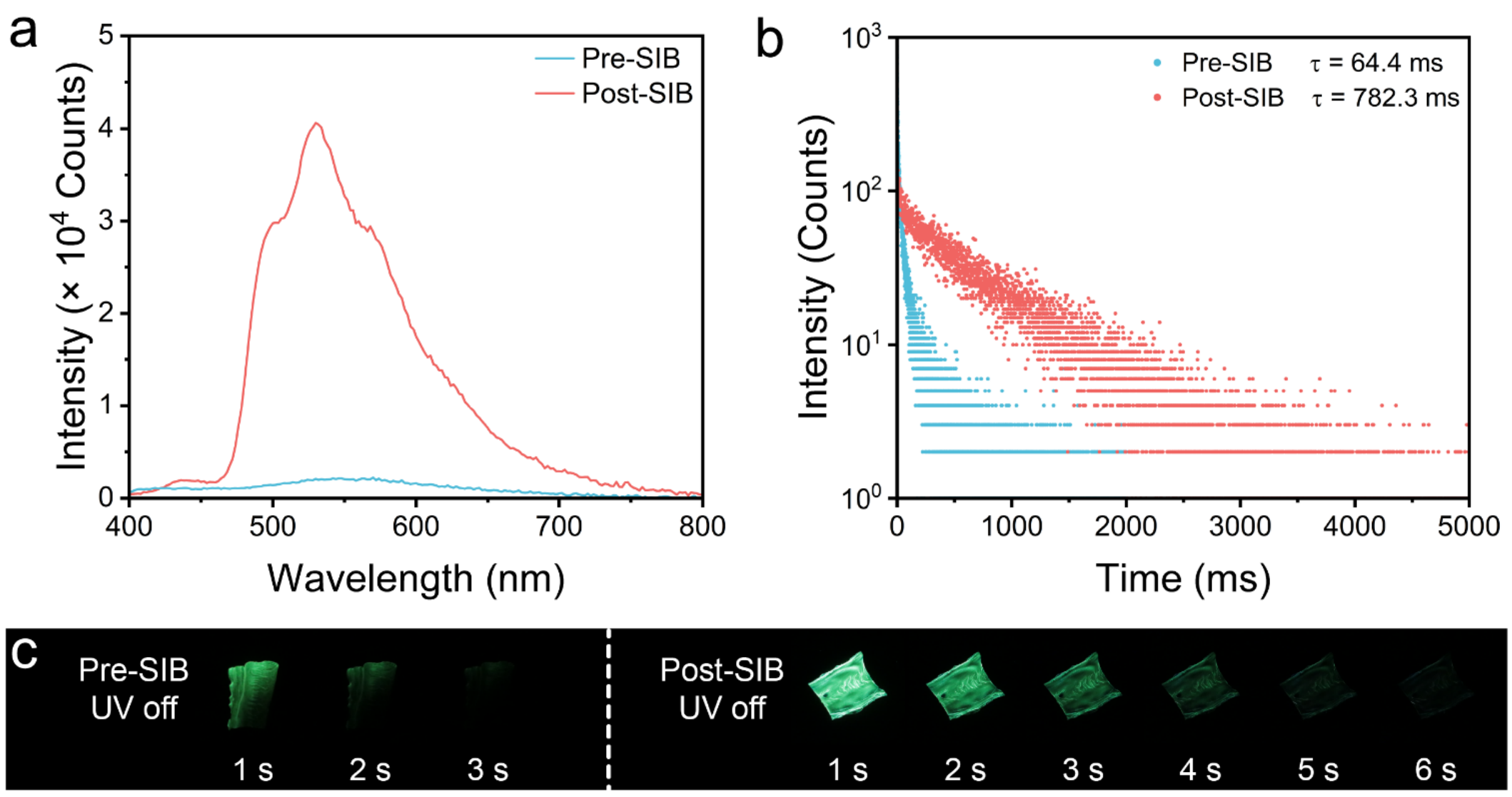

Supplementary Figure 16. Spectra and photographs. (**a**) Time-gated spectra, (**b**) phosphorescence decay curves and (**c**) afterglow photographs of *BINOL@PVA* before and after ISB treatment (pre-ISB and post-ISB) under 254 nm UV irradiation.

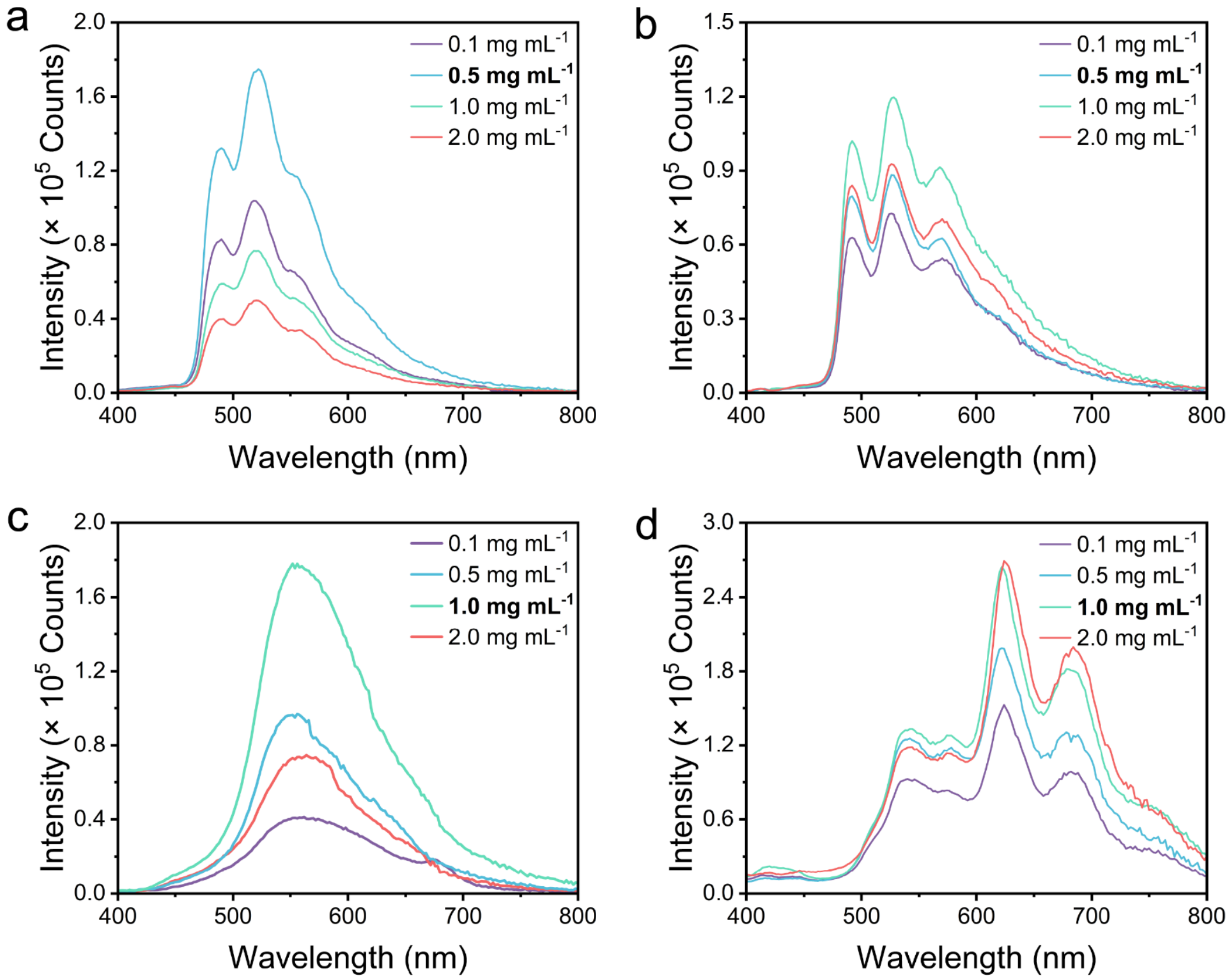

Supplementary Figure 17. Concentration-dependent time-gated spectra. (**a**) *DPBN@PVA*, (**b**) *DFBN@PVA*, (**c**) *DCBN@PVA* and (**d**) *DPCA@PVA*.

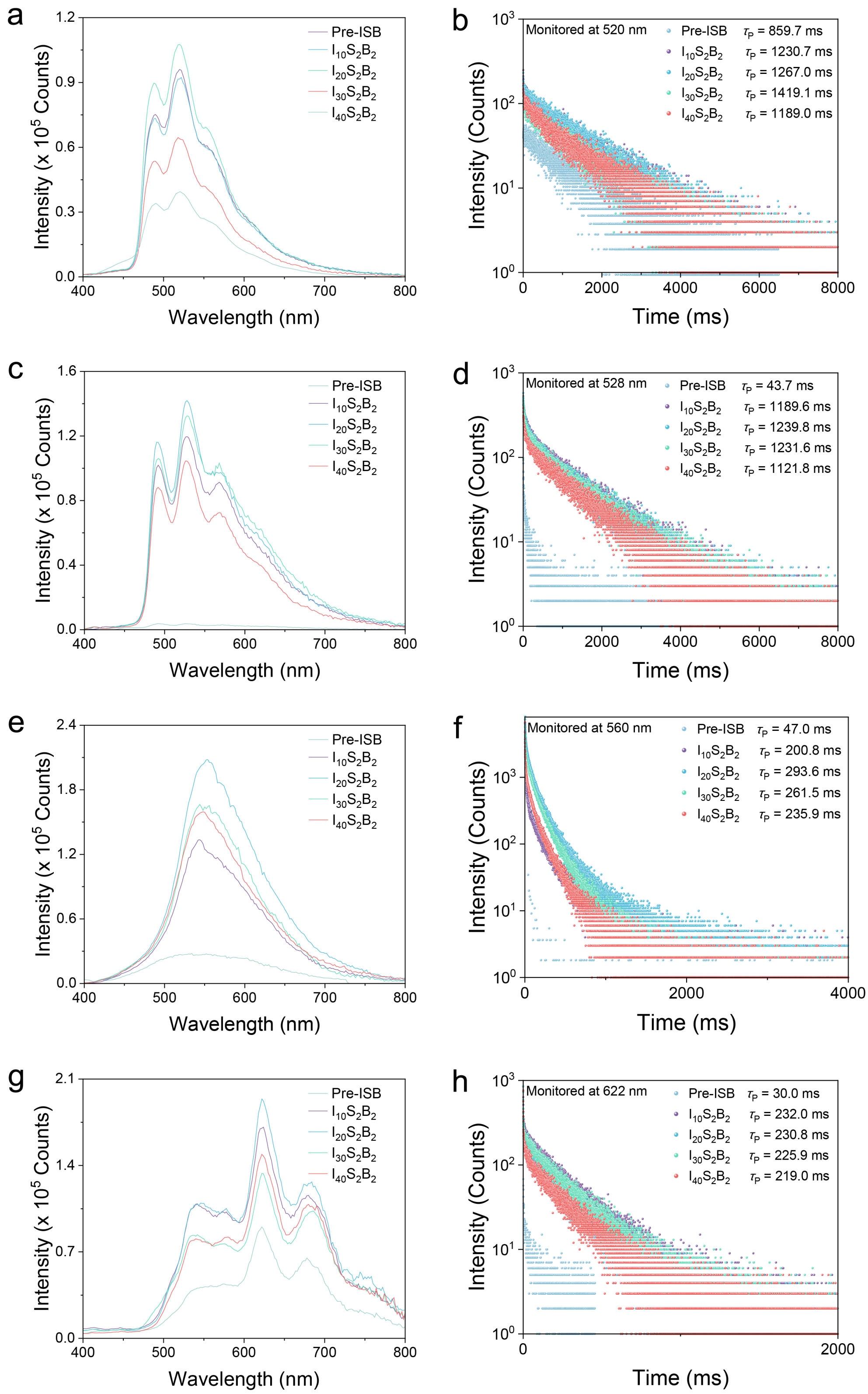

Supplementary Figure 18. Time-gated spectra and phosphorescence lifetime. Time-gated spectra of (**a**) *DPBN@PVA*, (**c**) *DFBN@PVA*, (**e**) *DCBN@PVA* and (**g**) *DPCA@PVA* films. Phosphorescence lifetime profiles of (**b**) *DPBN@PVA*, (**d**) *DFBN@PVA*, (**f**) *DCBN@PVA* and (**h**) *DPCA@PVA* films.

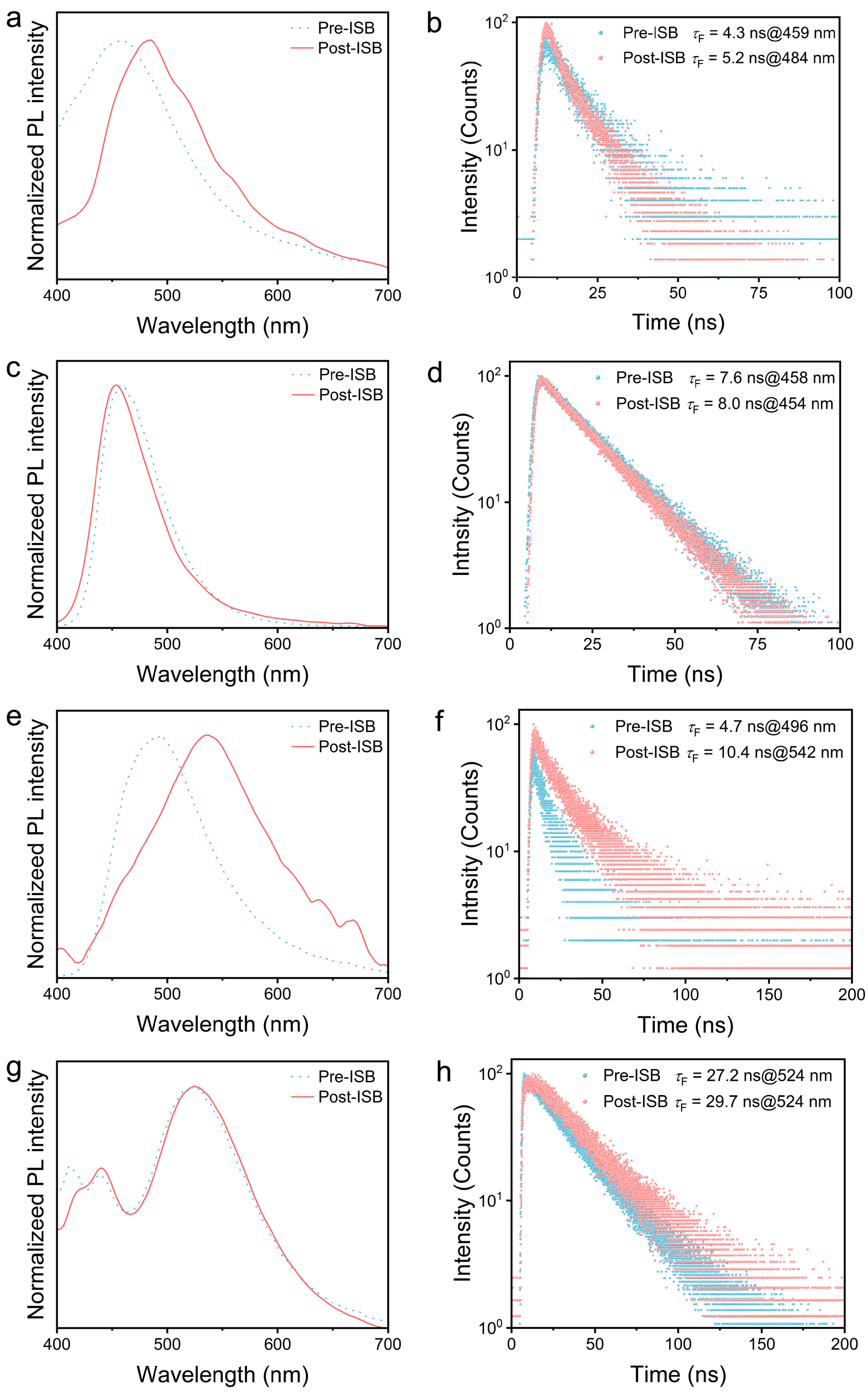

Supplementary Figure 19. Steady-state PL spectra and fluorescence lifetime. Steady-state PL spectra of (**a**) *DPBN@PVA*, (**c**) *DFBN@PVA*, (**e**) *DCBN@PVA* and (**g**) *DPCA@PVA*. Fluorescence lifetime profiles of (**b**) *DPBN@PVA*, (**d**) *DFBN@PVA*, (**f**) *DCBN@PVA* and (**h**) *DPCA@PVA*.

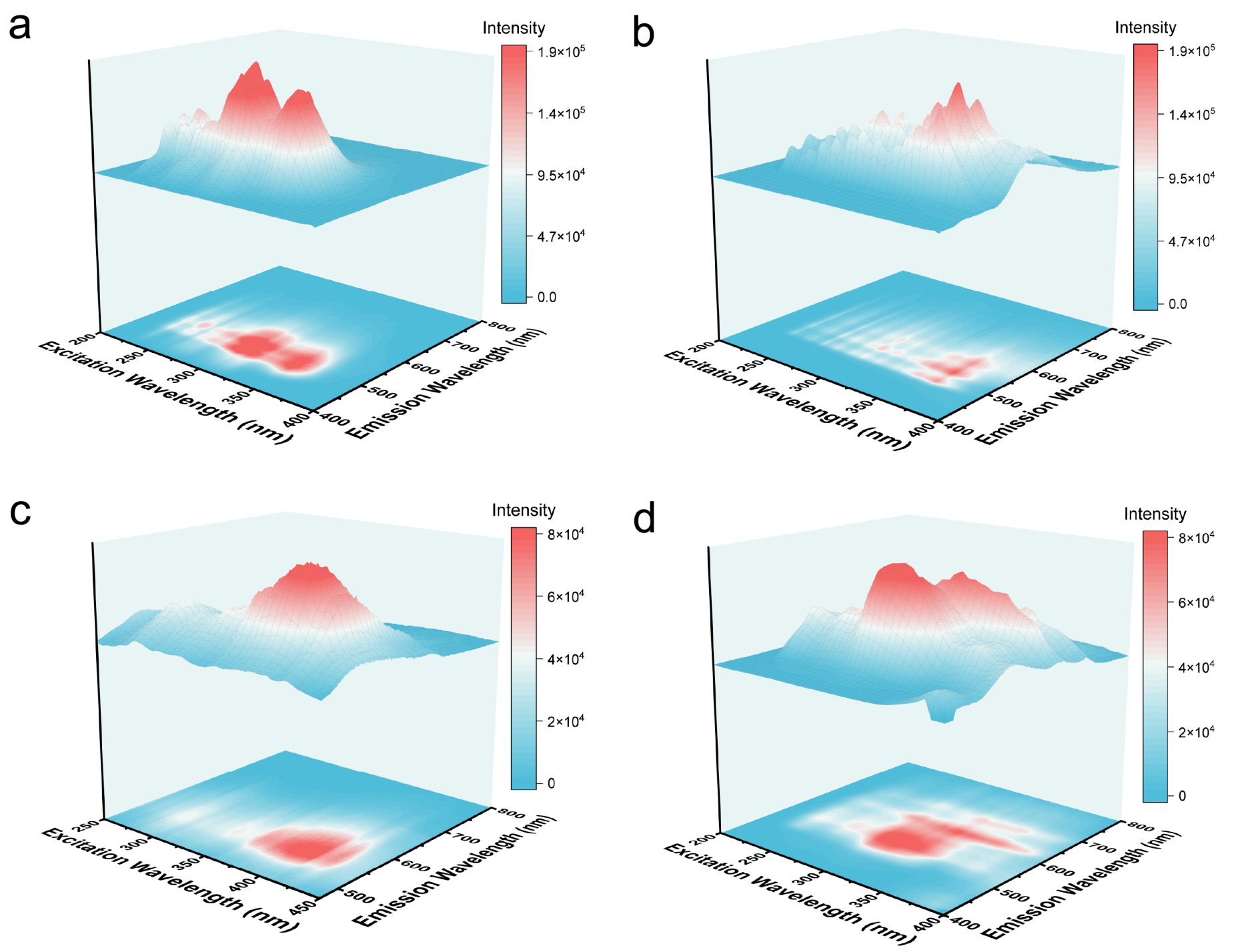

Supplementary Figure 20. Excitation-phosphorescence mapping. (**a**) *DPBN@PVA*, (**b**) *DFBN@PVA*, (**c**) *DCBN@PVA* and (**d**) *DPCA@PVA*.

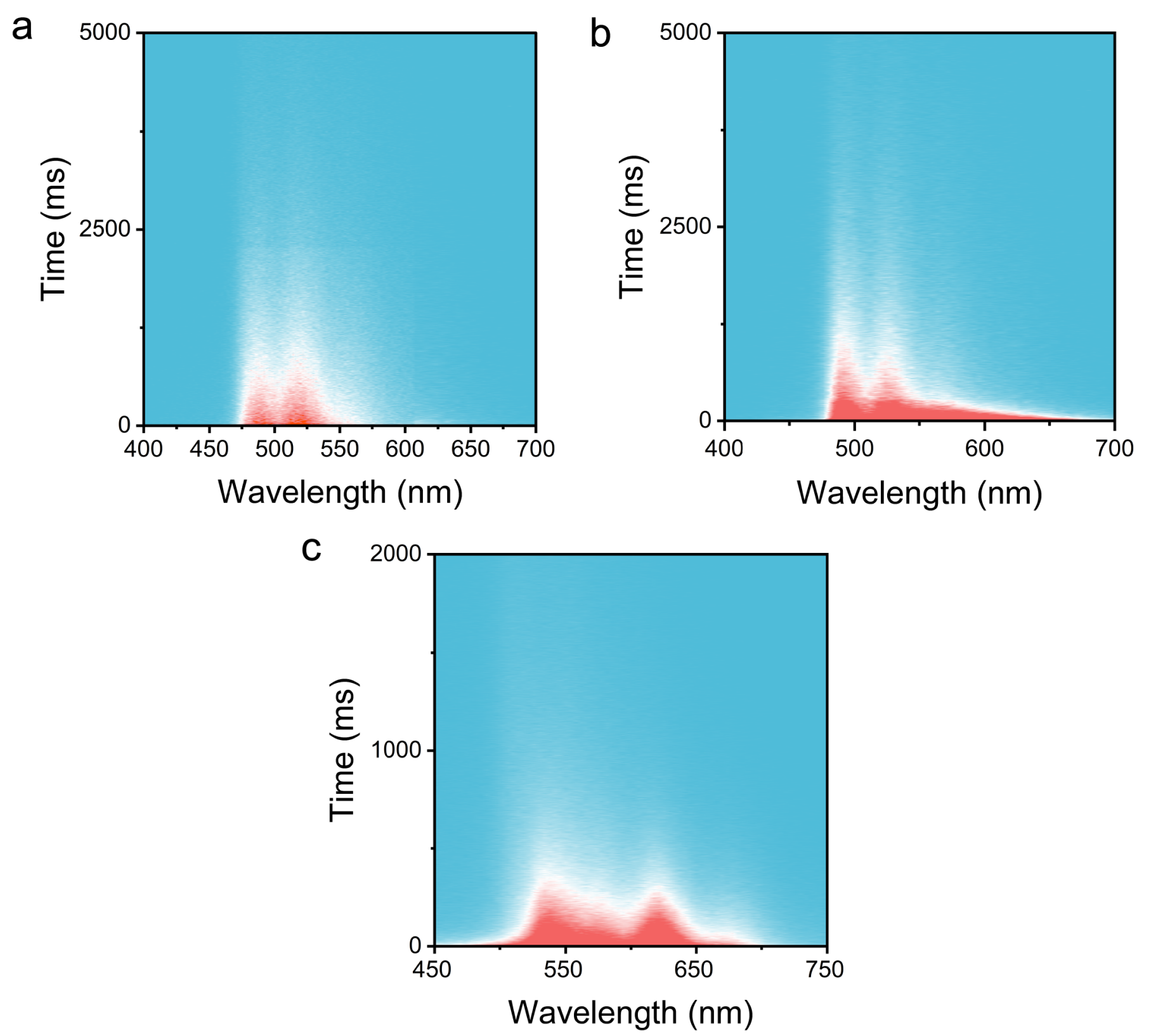

Supplementary Figure 21. Time resolved emission spectra. (**a**) *DPBN@PVA*, (**b**) *DFBN@PVA*, and (**c**) *DPCA@PVA*.

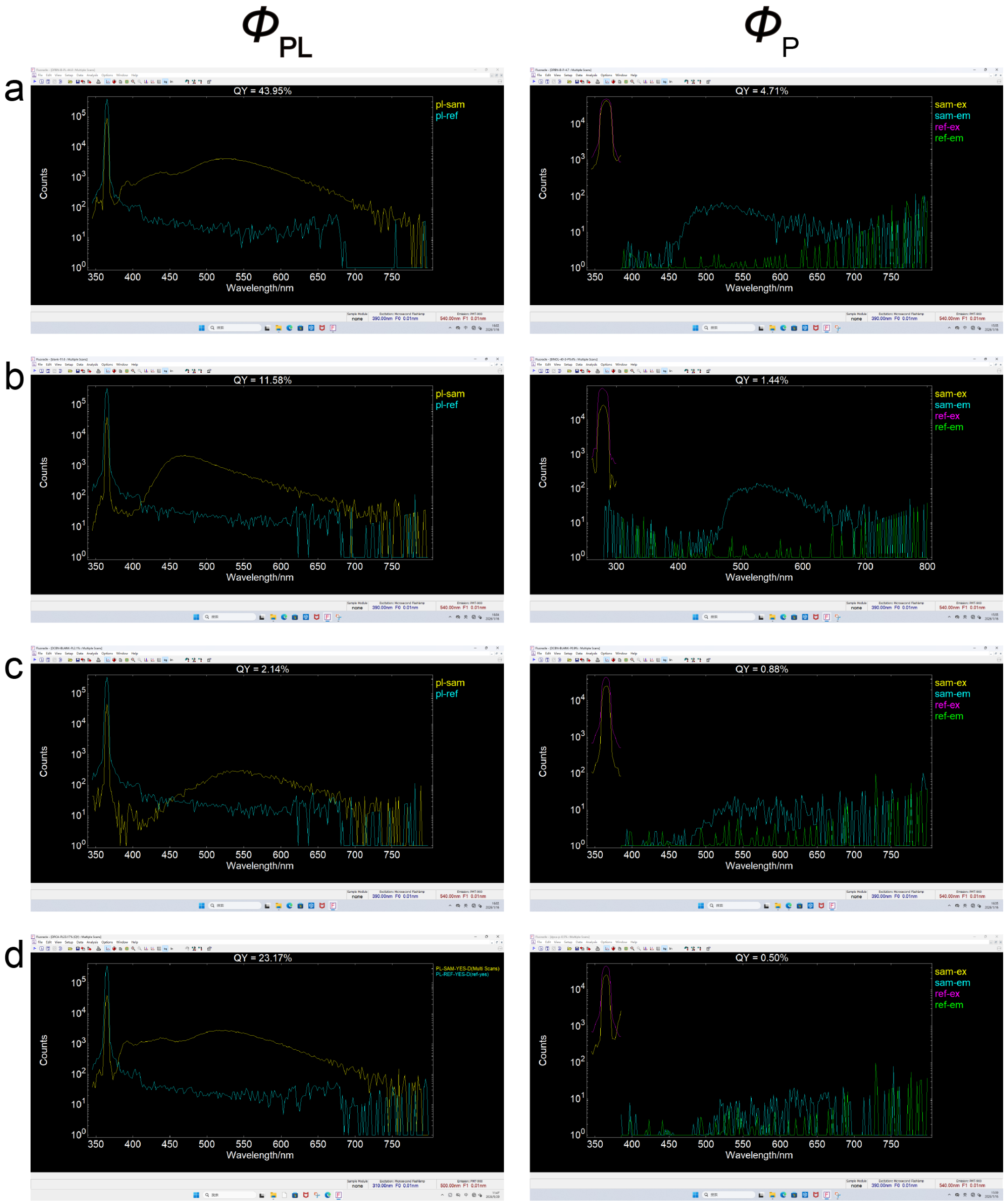

Supplementary Figure 22. Raw data for Φ_{PL} and Φ_P. (**a**) *DPBN@PVA*, (**b**) *DFBN@PVA*, (**c**) *DCBN@PVA*, and (**d**) *DPCA@PVA* films before ISB treatment. For *BINs@PVA*, TADF was excluded based on temperature-dependent spectroscopic measurements (Supplementary Figure 23). Therefore, Φ_{PL} can be considered as the sum of Φ_P and Φ_F. Experimentally, the absolute Φ_{PL} was measured using the same instrument equipped with a xenon lamp. The absolute Φ_P was determined using an integrating sphere equipped with a microsecond flash lamp on the Edinburgh FLS1000 spectrometer, enabling temporal discrimination of phosphorescence. Accordingly, the Φ_F was obtained by subtracting Φ_P from Φ_{PL}. The reference Φ_{PL} curves from Supplementary Figure 22b,c and Supplementary Figure 23d are the same.

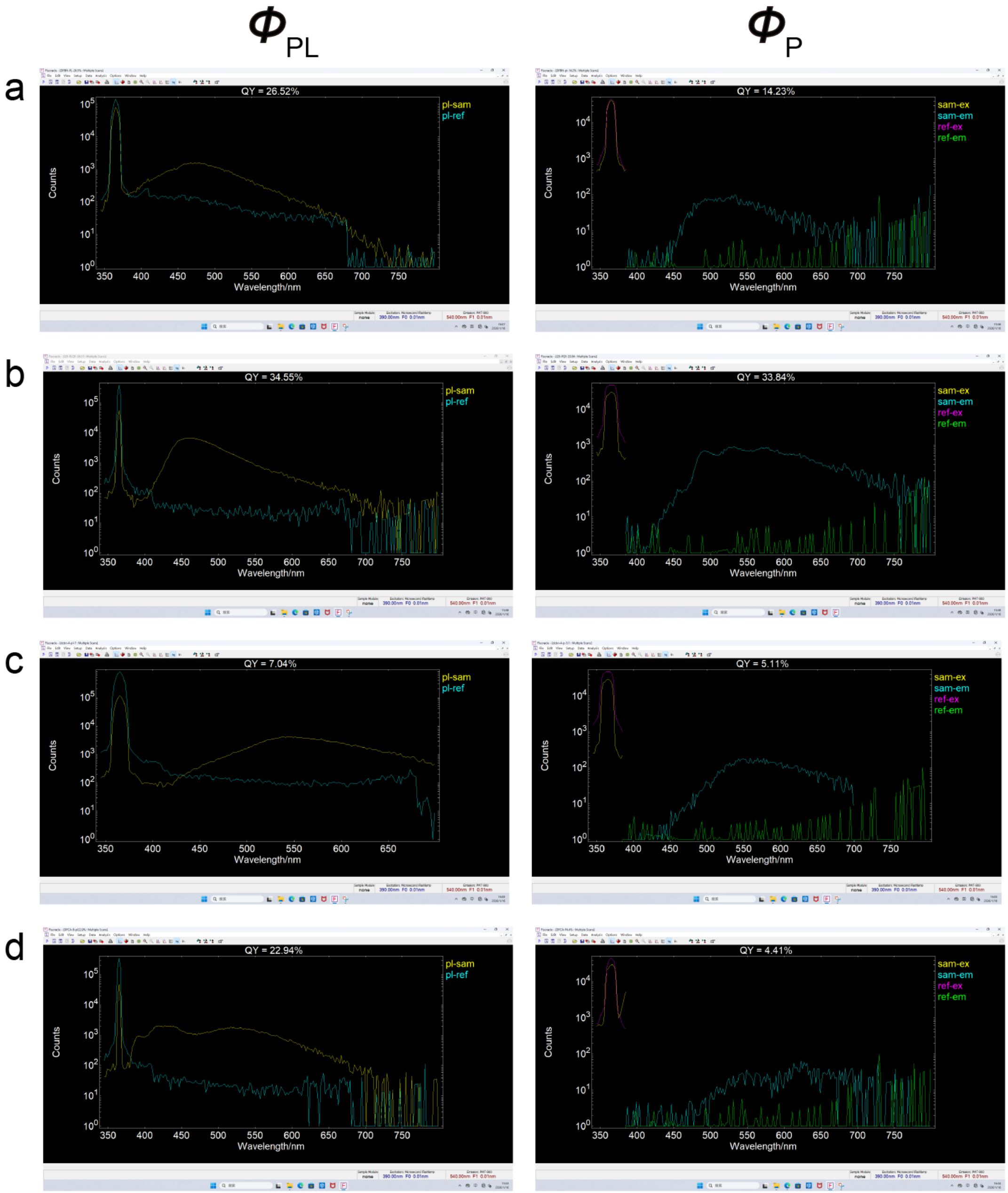

Supplementary Figure 23. **Raw data for Φ_{PL} and Φ_P.** (**a**) *DPBN@PVA*, (**b**) *DFBN@PVA*, (**c**) *DCBN@PVA*, and (**d**) *DPCA@PVA* films after ISB treatment. The reference Φ_{PL} curves from Supplementary Figure 22b,c and Supplementary Figure 23d are the same.

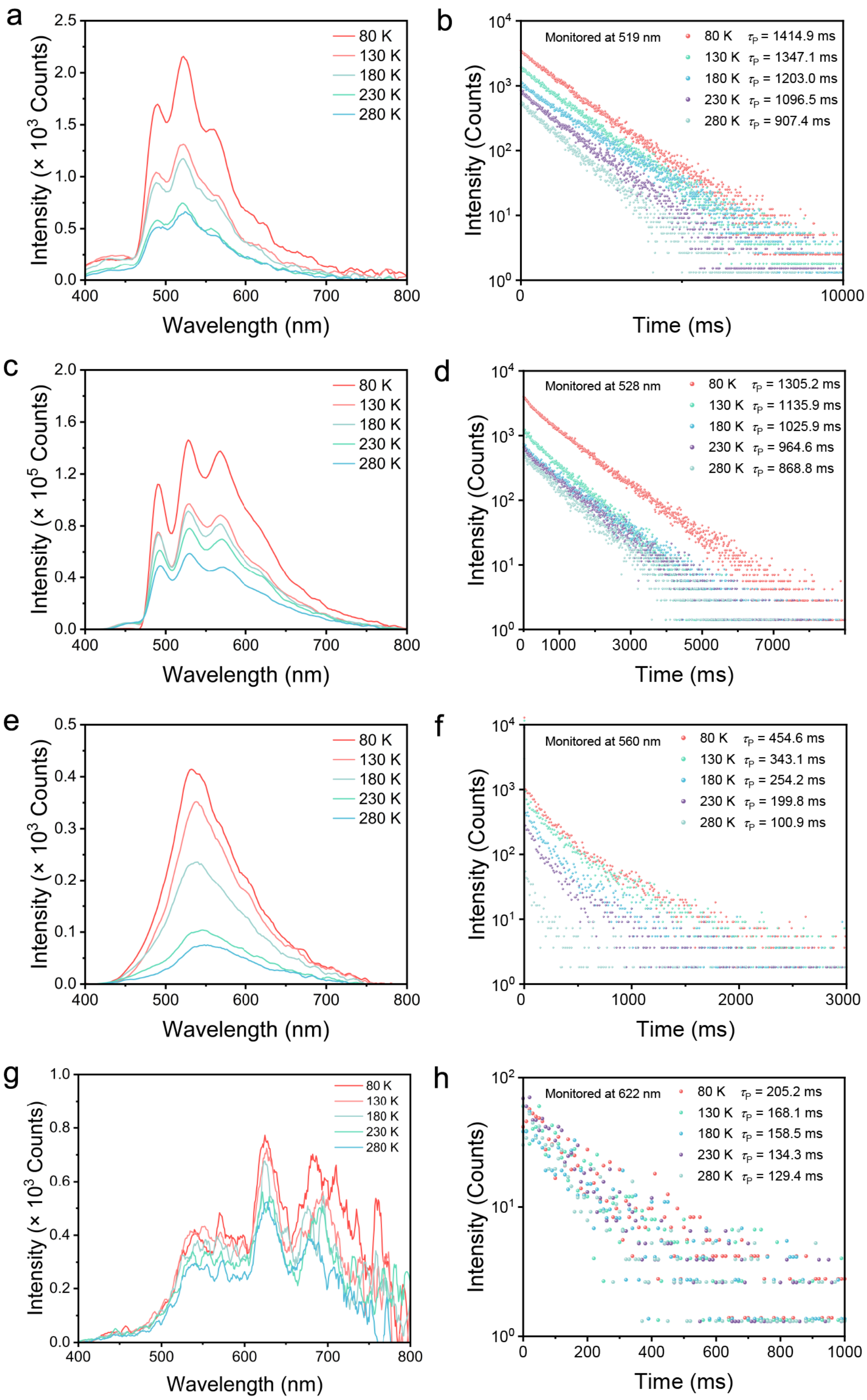

Supplementary Figure 24. Temperature-dependent results. Temperature-dependent time-gated spectra of (**a**) *DPBN@PVA*, (**c**) *DFBN@PVA*, (**e**) *DCBN@PVA* and (**g**) *DPCA@PVA*. Temperature-dependent phosphorescence lifetime decay profiles of (**b**) *DPBN@PVA*, (**d**) *DFBN@PVA*, (f) *DCBN@PVA* and (h) *DPCA@PVA*.

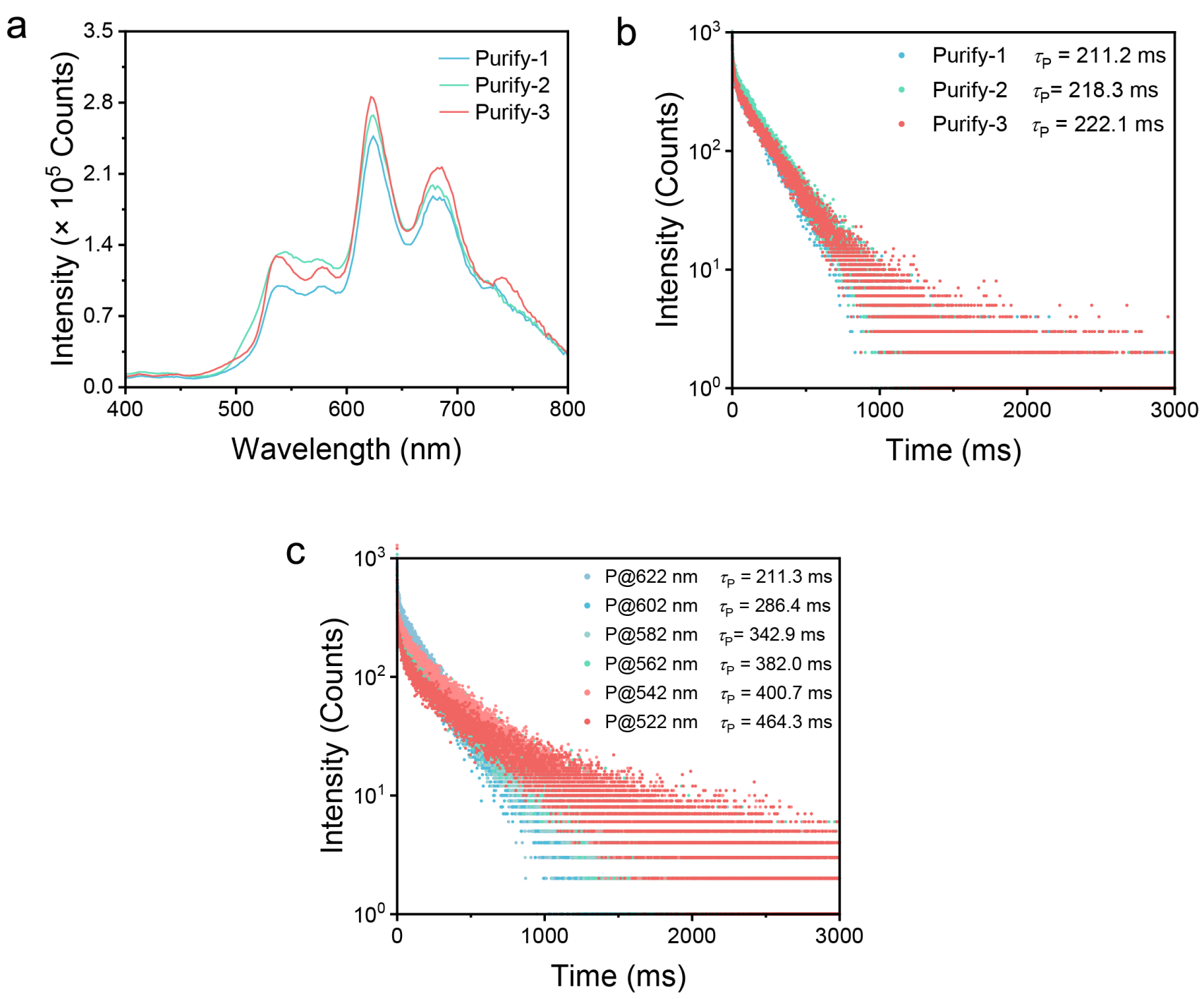

Supplementary Figure 25. Time-gated spectra and phosphorescence decay curves. (a) Time-gated and **(b)** phosphorescence decay curves (λ_{em} = 622 nm) of post-ISB *DPCA@PVA* films after the purification. **(c)** Phosphorescence decay curves of post-ISB *DPCA@PVA* film at different emission peaks.

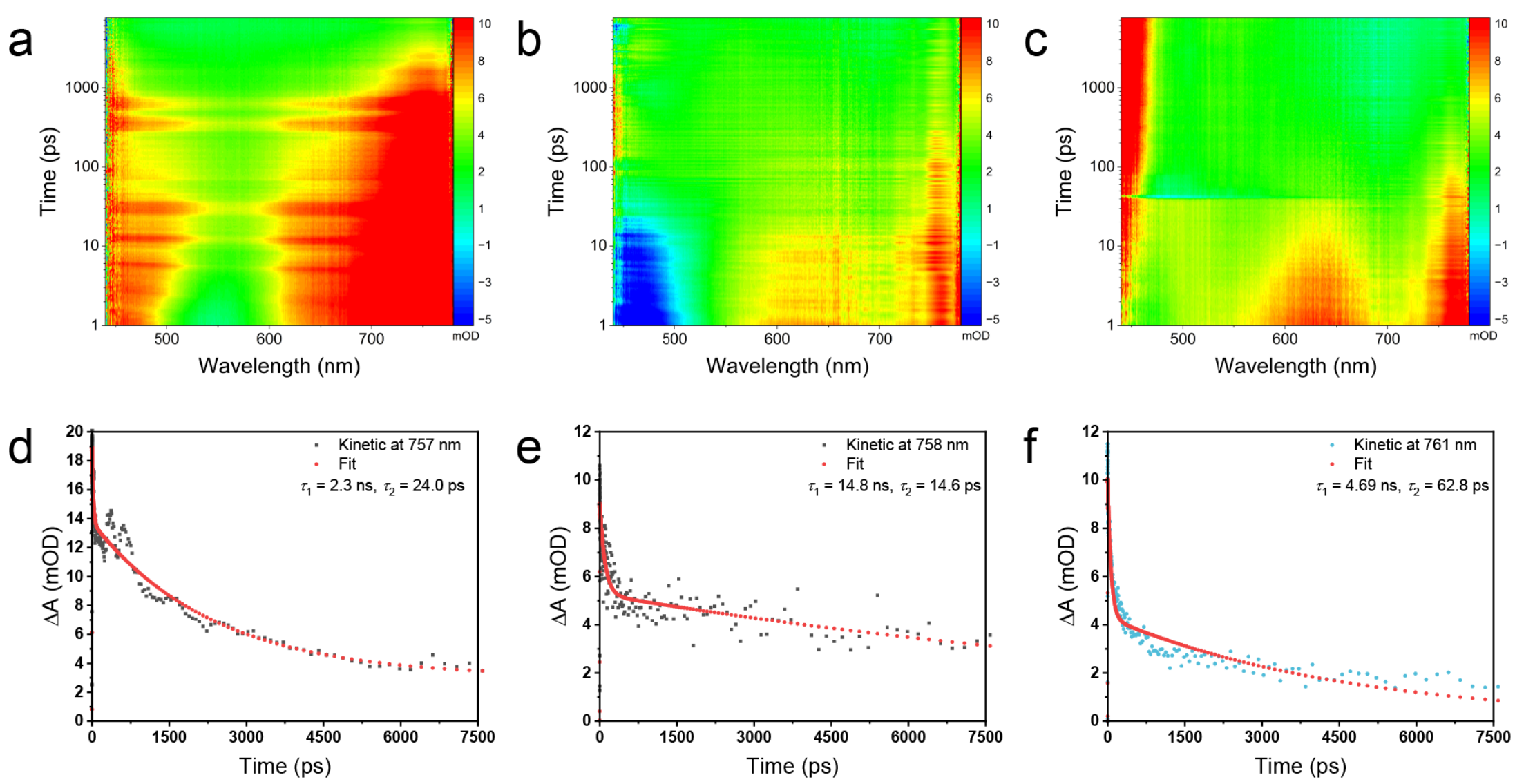

Supplementary Figure 26. Time-resolved absorption spectra and kinetic curves. Time-resolved absorption spectra of (**a**) isolated *DCBN*, (**b**) Pre-ISB *DCBN@PVA* film and (**c**) Post-ISB *DCBN@PVA* film. Kinetic curves of excited state absorption for (**d**) isolated DCBN, (**e**) Pre-ISB *DCBN@PVA* film and (**f**) Post-ISB *DCBN@PVA* film. *DCBN* is dispersed in *DMSO*.

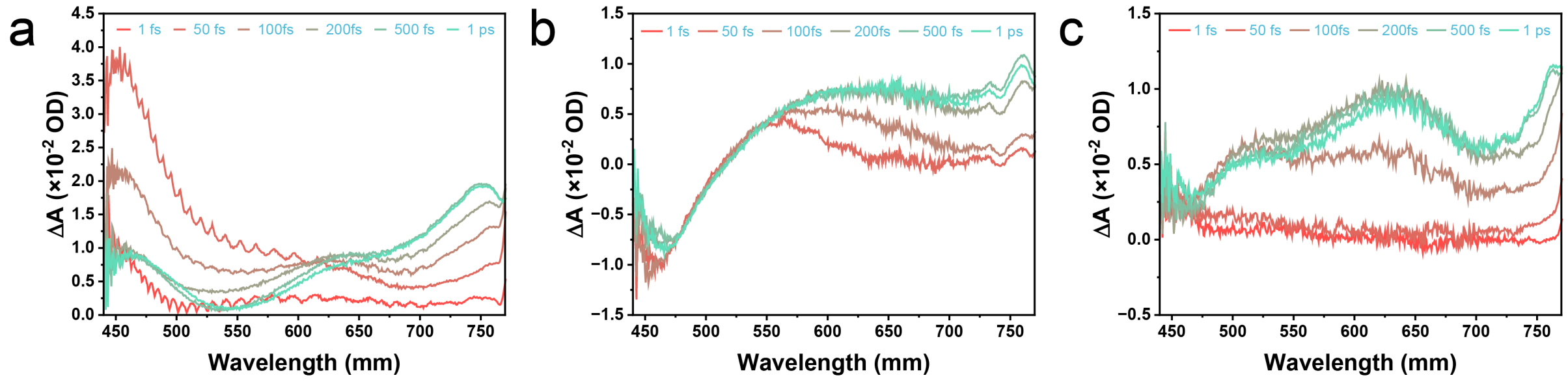

Supplementary Figure 27. Transient absorption spectra. (**a**) Isolated *DCBN* in *DMSO*, (**b**) Pre-ISB *DCBN@PVA* films and (**c**) Post-ISB *DCBN@PVA* films ranging from 1 fs to 1 ps.

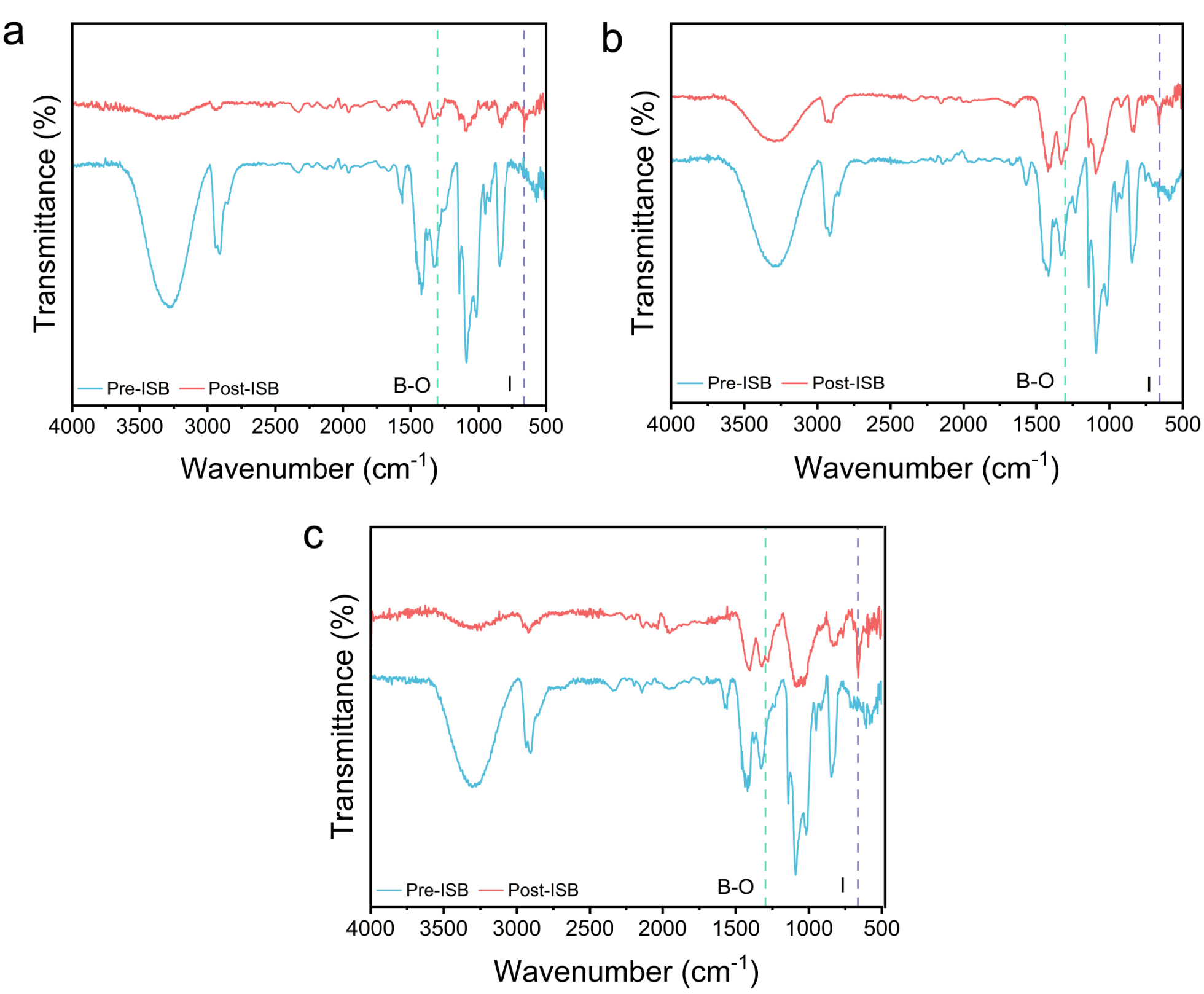

Supplementary Figure 28. FT-IR spectra. (**a**) *DPBN@PVA*, (**b**) *DCBN@PVA* and (**c**) *DPCA@PVA* upon pre- and post-ISB treatment.

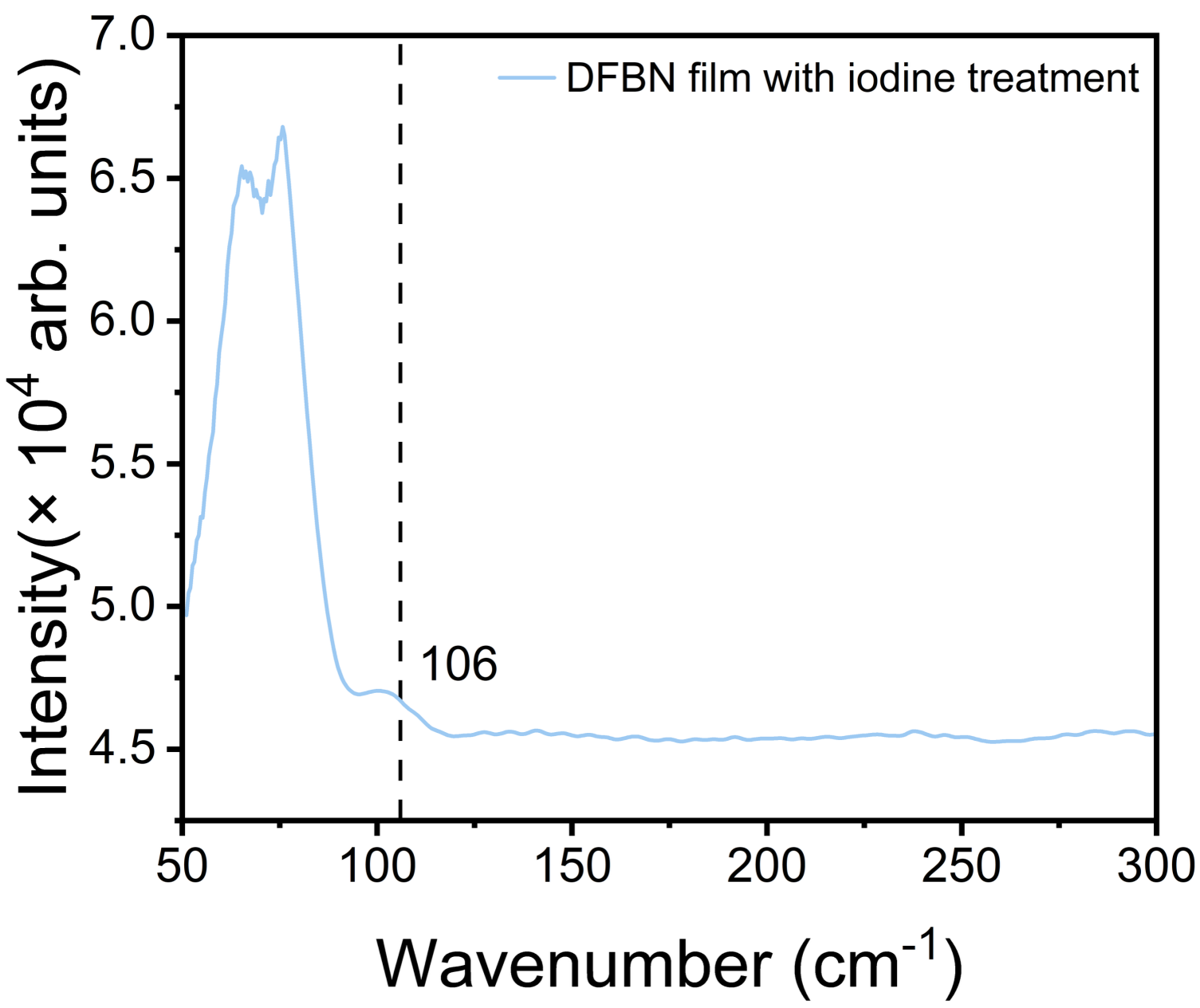

Supplementary Figure 29. Raman spectrum of *DFBN@PVA* film with iodine treatment.

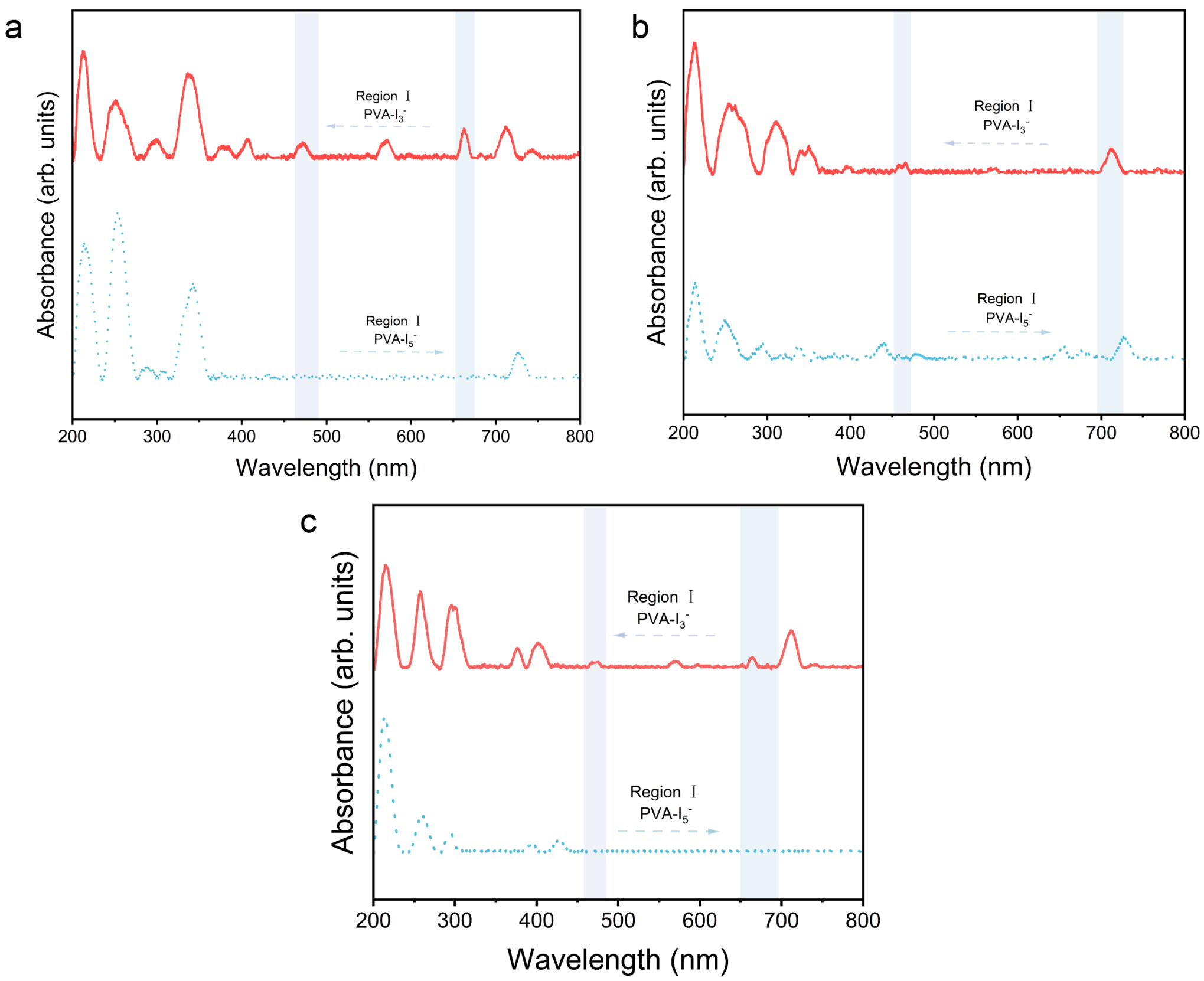

Supplementary Figure 30. Solid UV-vis spectra. (**a**) *DPBN@PVA*, (**b**) *DCBN@PVA* and (**c**) *DPCA@PVA*. The dashed line is for pristine film and the solid line is for ISB treated film.

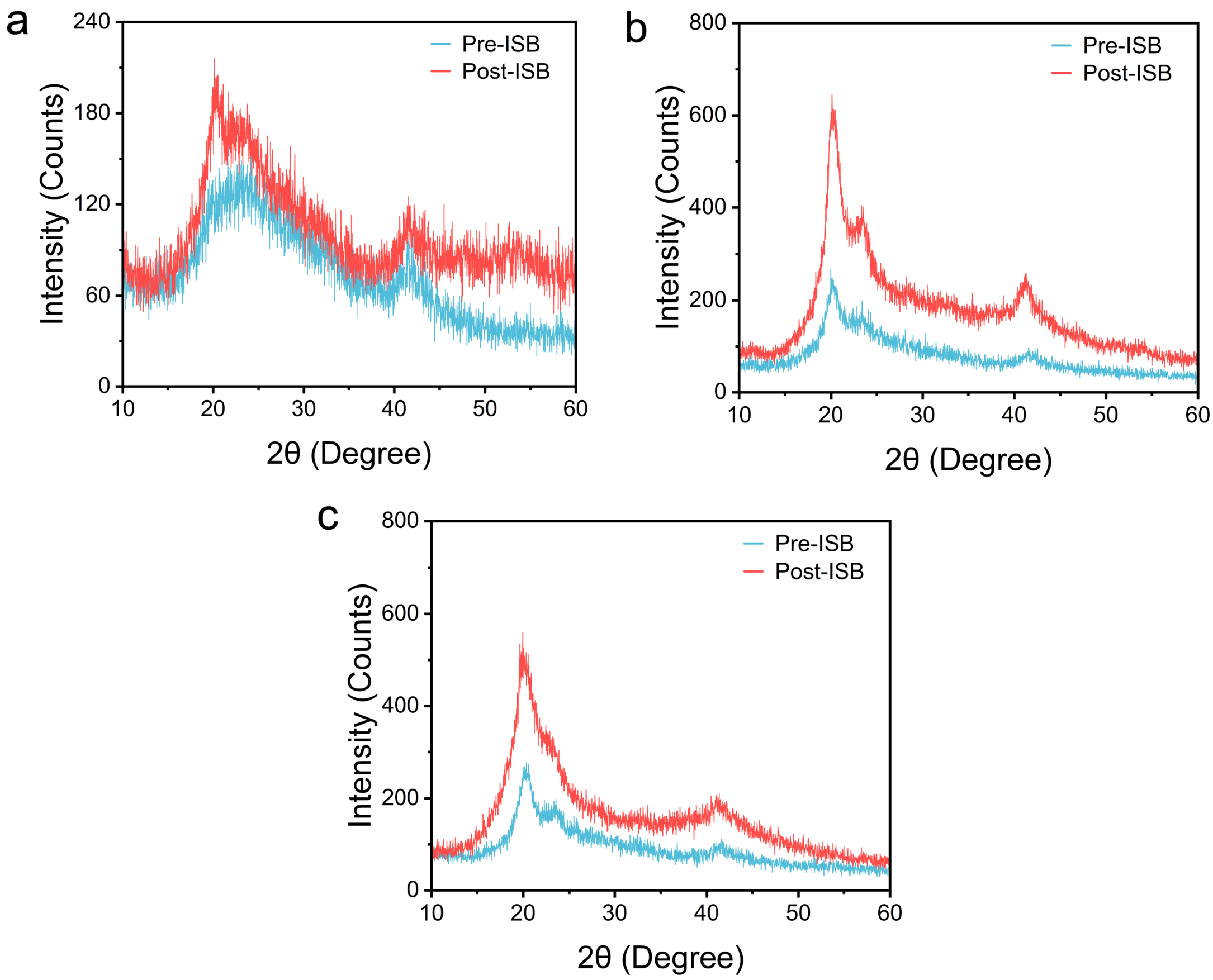

Supplementary Figure 31. Powder XRD patterns. (**a**) *DPBN@PVA*, (**b**) *DCBN@PVA* and (**c**) *DPCA@PVA* films upon pre- and post-ISB treatment.

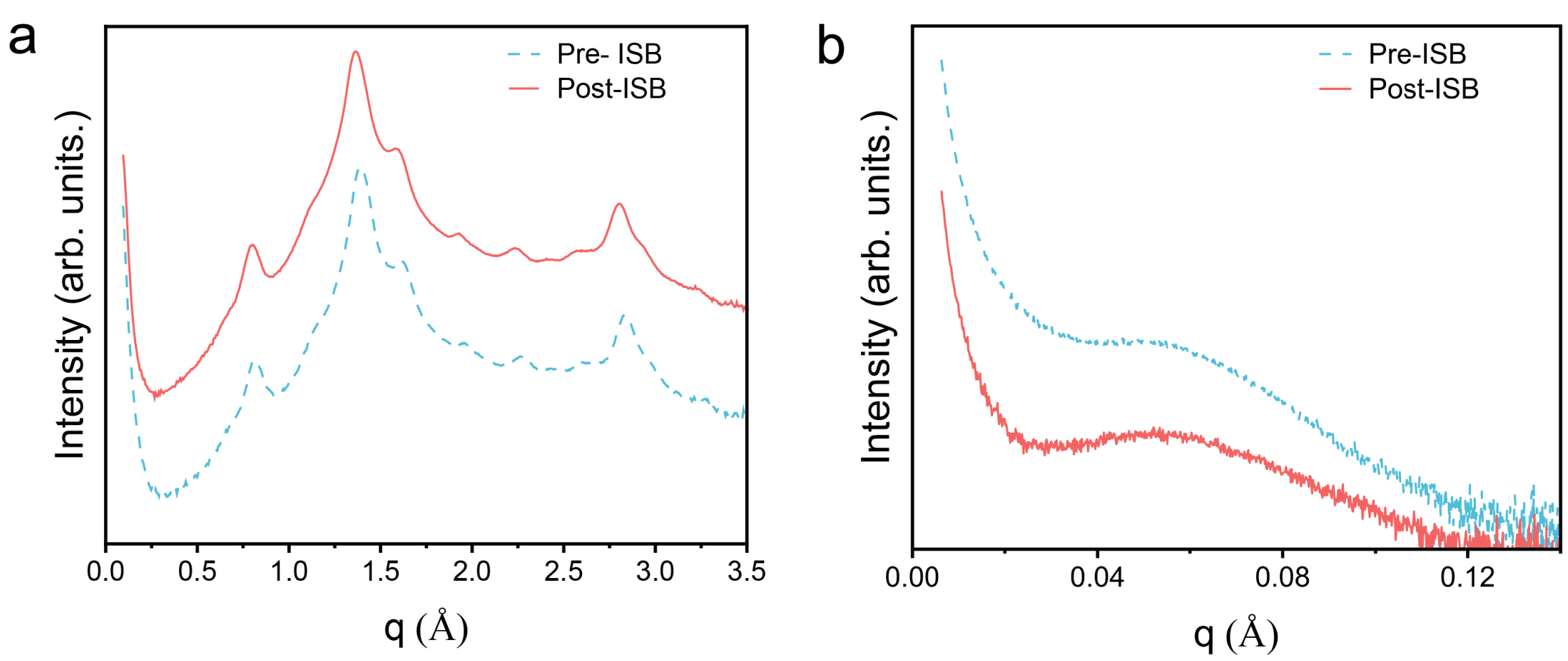

Supplementary Figure 32. 1D WAXS and 1D SAXS. (a) 1D WAXS and **(b)** 1D SAXS profiles of *DFBN@PVA* upon pre- and post-ISB treatment.

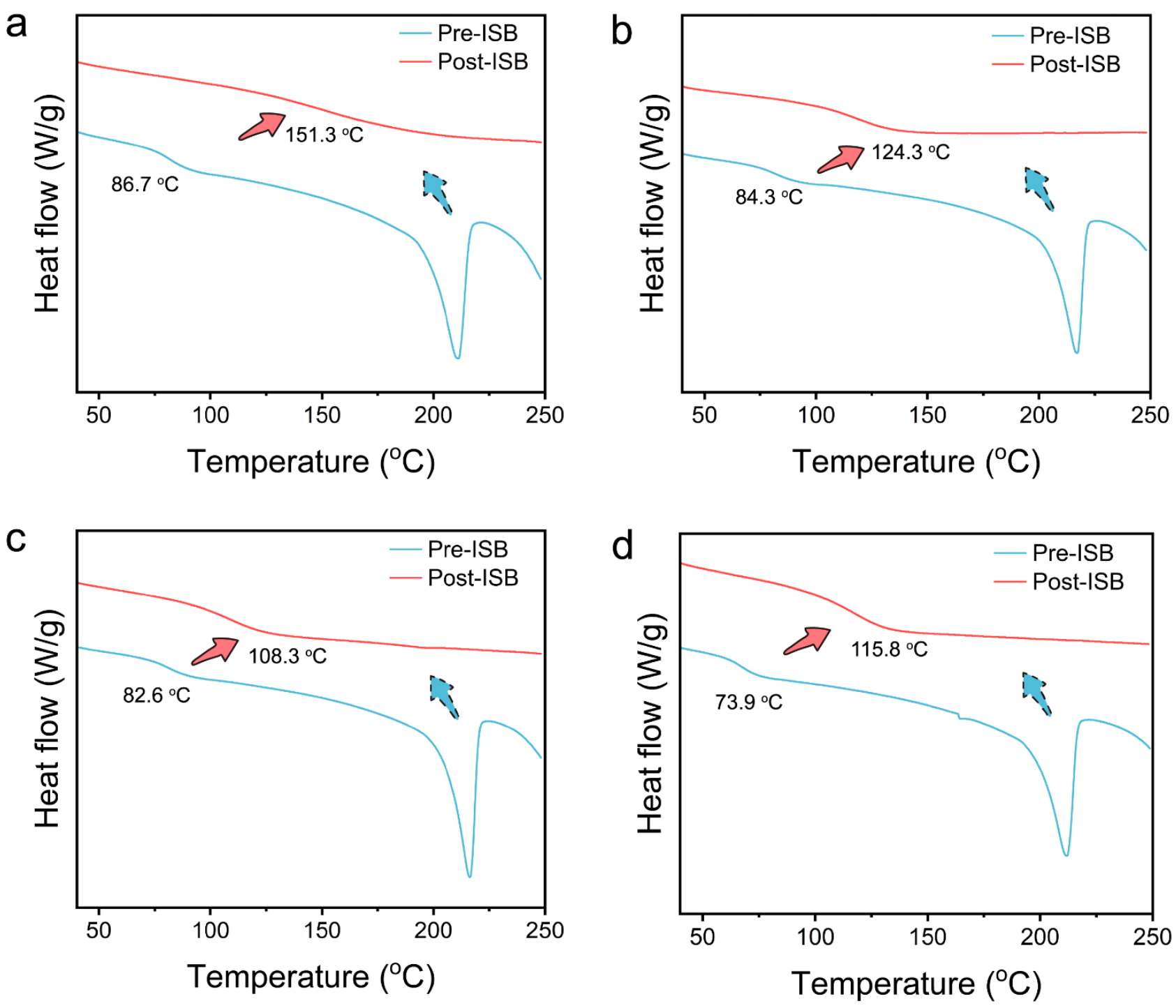

Supplementary Figure 33. DSC curves. (**a**) *DPBN@PVA*, (**b**) *DFBN@PVA*, (**c**) *DCBN@PVA* and (**d**) *DPCA@PVA* upon pre- and post-ISB treatment. T_g is defined as the midpoint temperature of the baseline shift observed in the heat flow-temperature curve of the DSC.

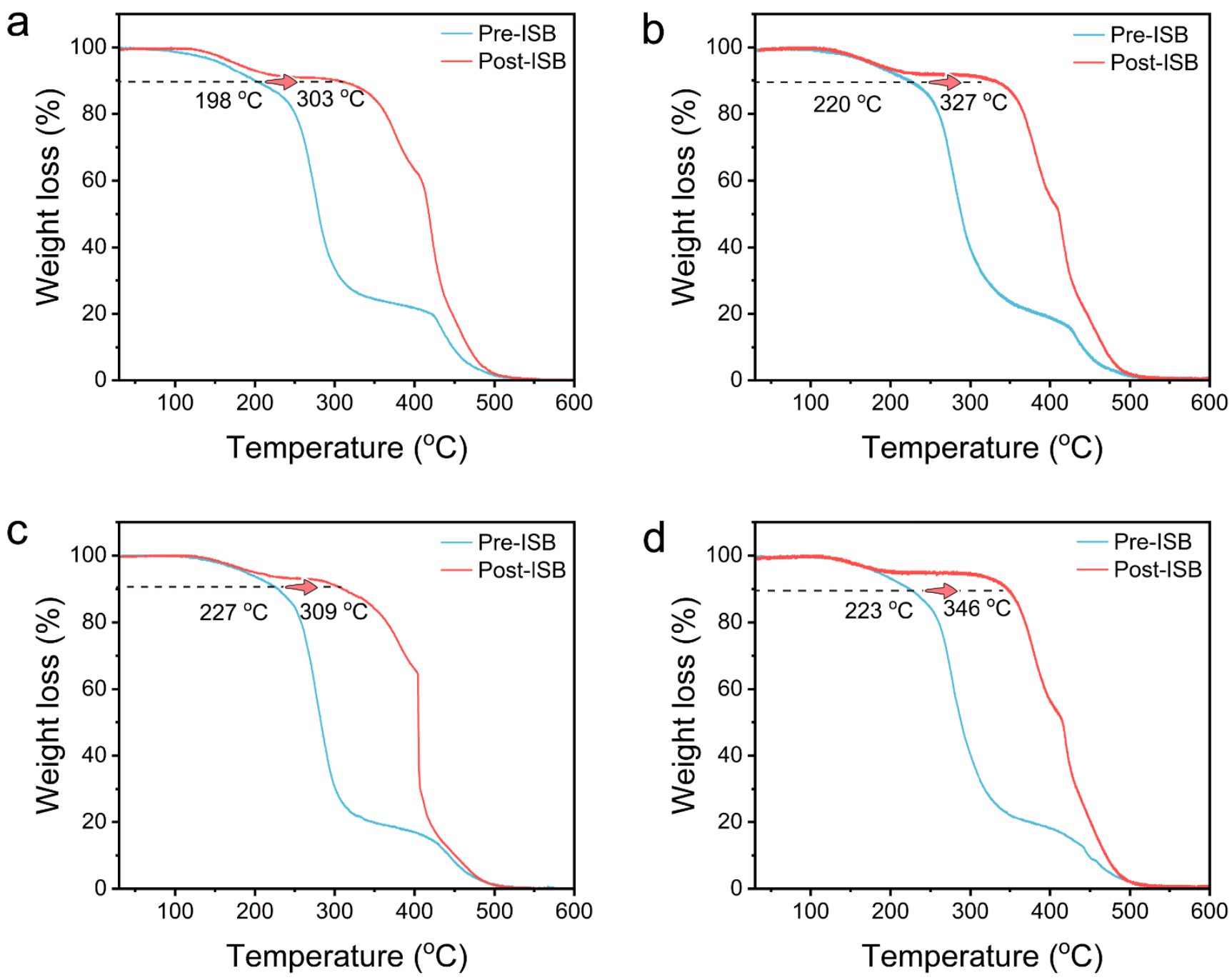

Supplementary Figure 34. TGA curves. (**a**) *DPBN@PVA*, (**b**) *DFBN@PVA*, (**c**) *DCBN@PVA* and (**d**) *DPCA@PVA* upon pre- and post-ISB treatment. T_d is defined as the temperature corresponding to 10% weight loss in TGA curve.

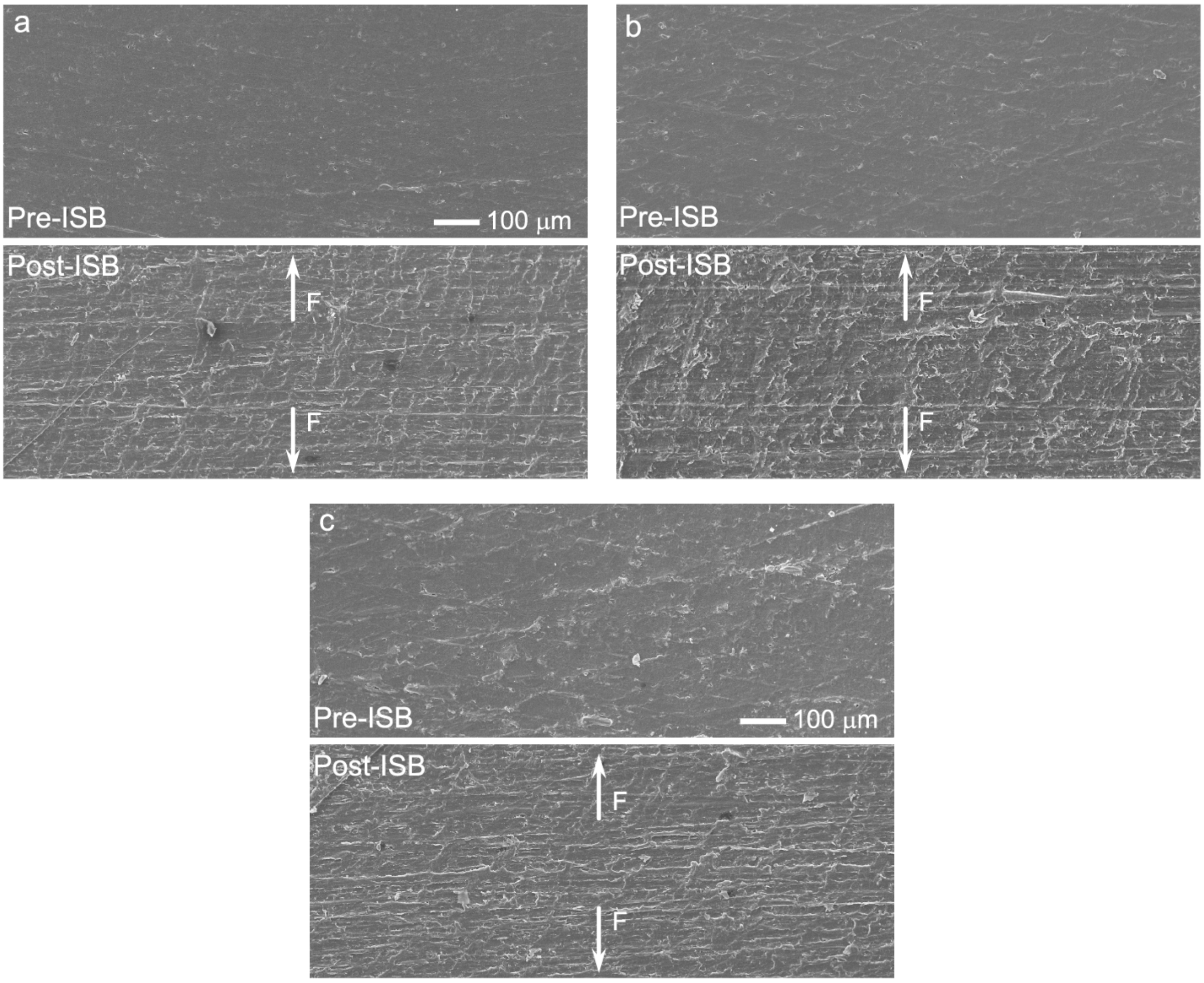

Supplementary Figure 35. SEM images. (**a**) *DPBN@PVA*, (**b**) *DCBN@PVA* and (**c**) *DPCA@PVA* upon pre- and post-ISB treatment.

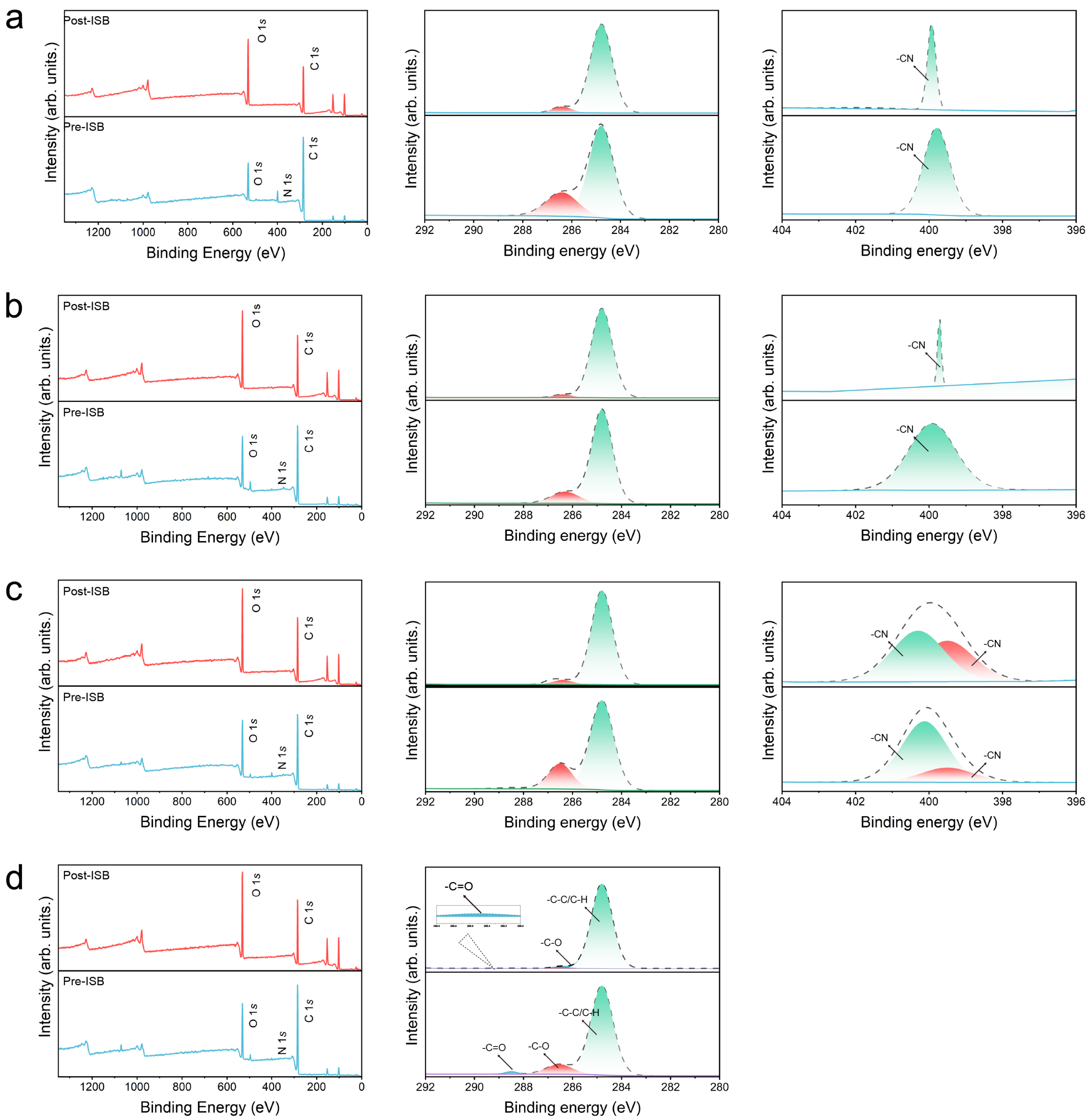

Supplementary Figure 36. Raw XPS spectra and corresponding deconvoluted C 1*s* and N 1*s* spectra. (**a**) *DPBN@PVA*, (**b**) *DFBN@PVA*, (**c**) *DCBN@PVA* and (**d**) *DPCA@PVA*.

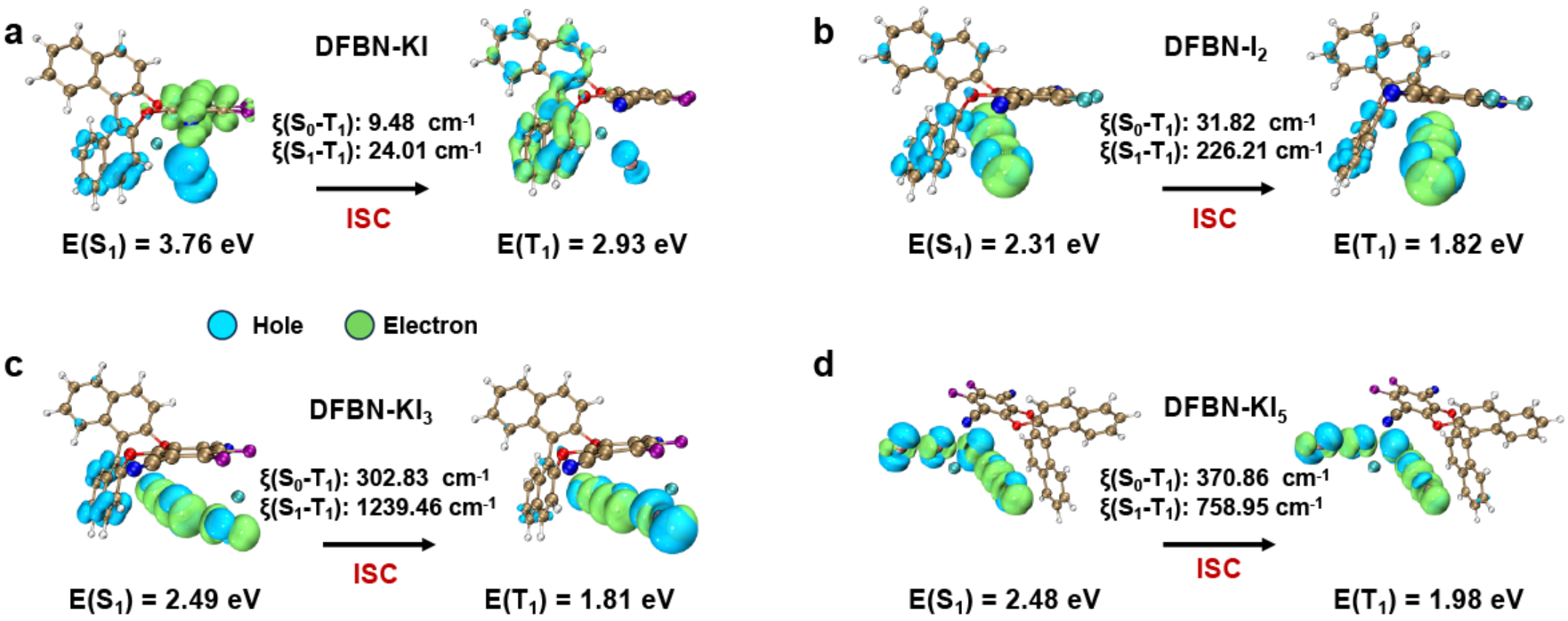

Supplementary Figure 37. **Electron-hole analysis, SOC constants, and energy levels of the S_1 and T_1 states**. (**a**) *DFBN-KI*, (**b**) *DFBN-I₂*, (**c**) *DFBN-KI₃*, and (**d**) *DFBN-KI₅* models based on their S_0 optimized geometry.

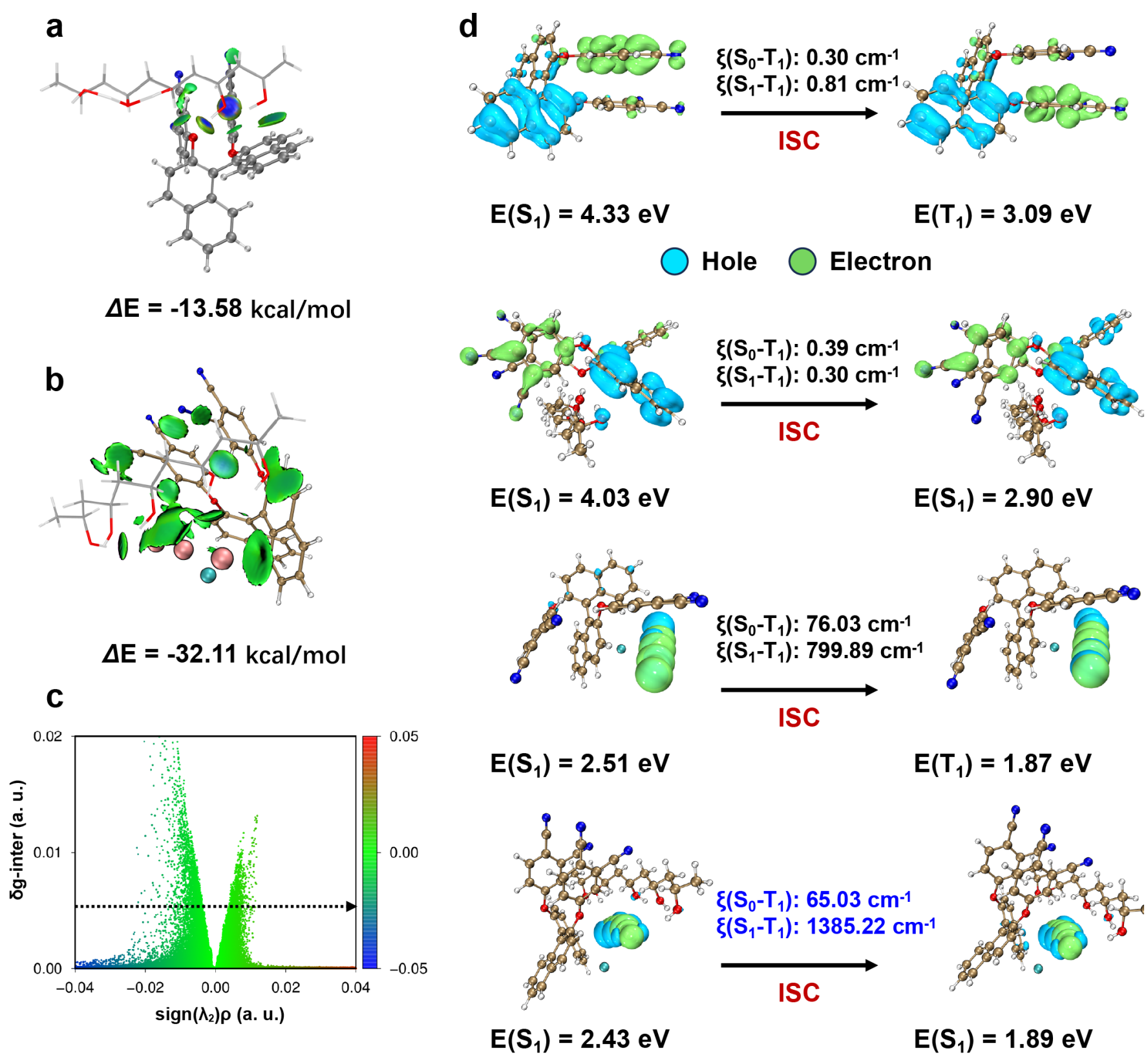

Supplementary Figure 38. Calculation results. Intermolecular interaction energies (considered on BSSE corrections) and IGMH analysis ($\delta_{g\text{-inter}} = 0.005$ a. u.) of (**a**) *DPBN@PVA* and (**b**) *DPBN-KI₃@PVA* model, respectively. (**c**) Distribution of different interactions between *PVA* and *DPBN-KI₃* by IGMH scatter map. (**d**) Electron-hole analysis, SOC constants, and energy levels of the S_1 and T_1 states for *DPBN*, *DPBN@PVA*, *DPBN-KI₃*, and *DPBN-KI₃@PVA* models based on their S_0 optimized geometry.

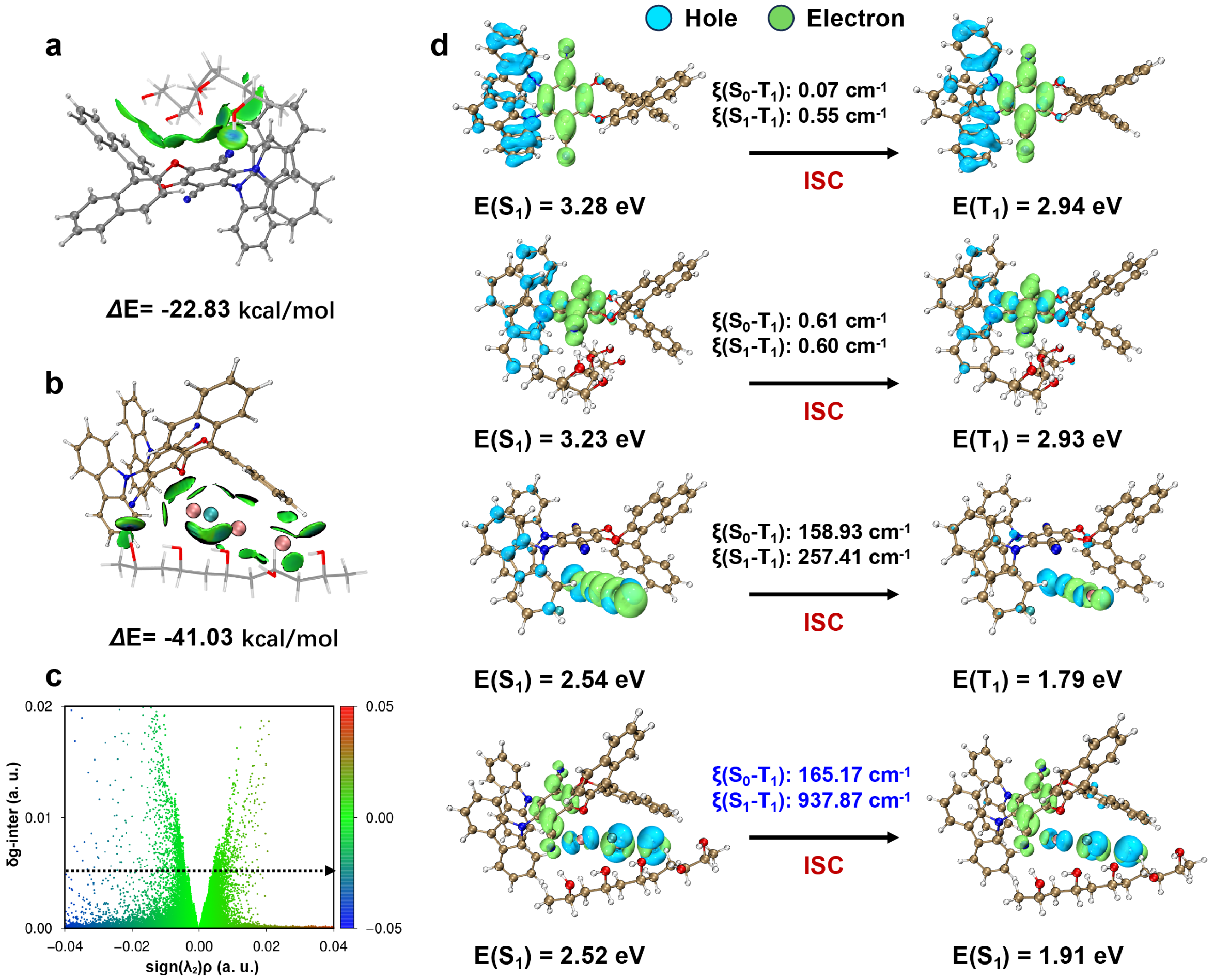

Supplementary Figure 39. Calculation results. Intermolecular interaction energies (considered on BSSE corrections) and IGMH analysis ($\delta_{g\text{-inter}} = 0.005$ a. u.) of (**a**) *DCBN@PVA* and (**b**) *DCBN-KI₃@PVA* model, respectively. (**c**) Distribution of different interactions between *PVA* and *DCBN-KI₃* by IGMH scatter map. (**d**) Electron-hole analysis, SOC constants, and energy levels of the S_1 and T_1 states for *DCBN*, *DCBN@PVA*, *DCBN-KI₃*, and *DCBN-KI₃@PVA* models based on their S_0 optimized geometry.

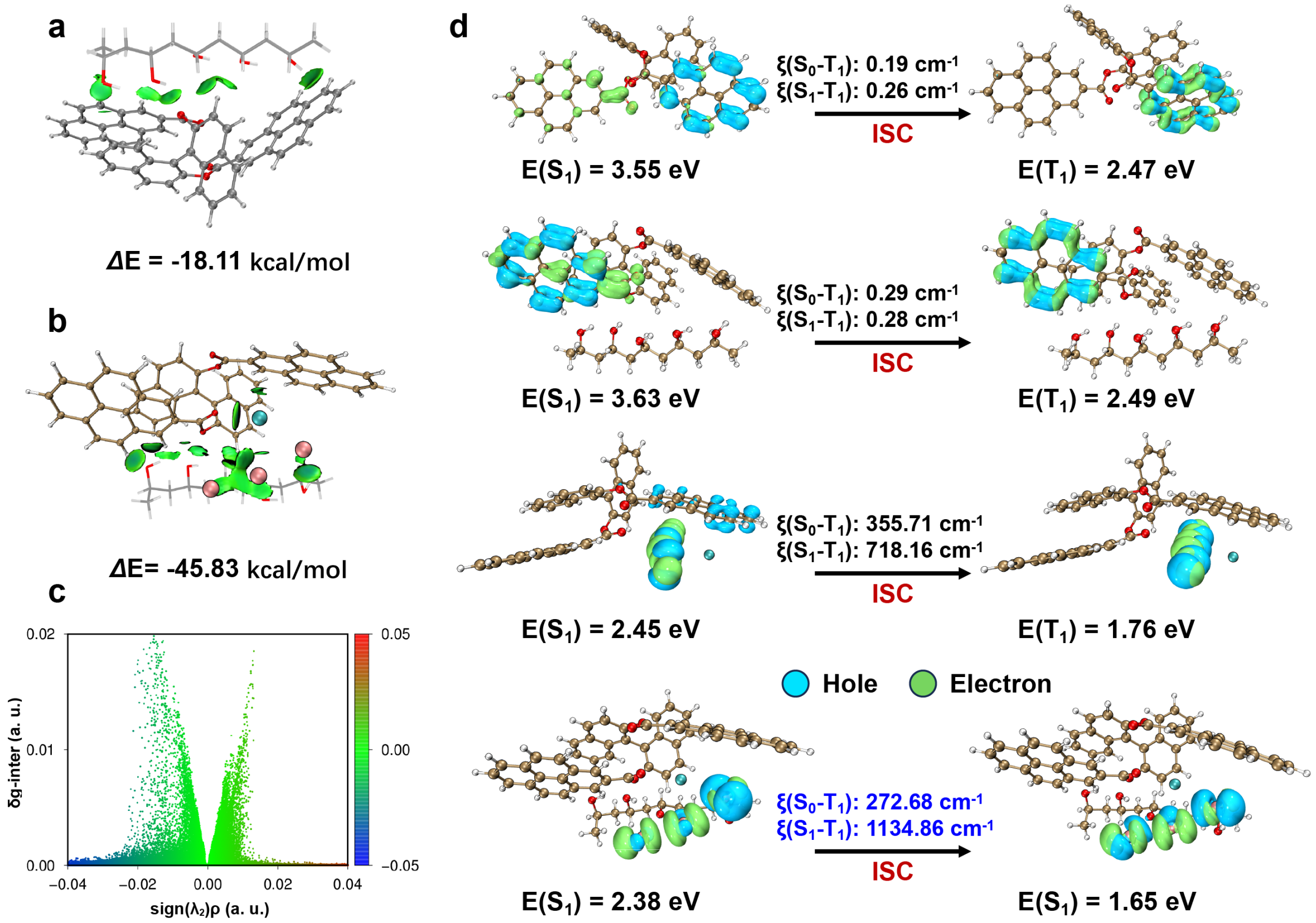

Supplementary Figure 40. Calculation results. Intermolecular interaction energies (considered on BSSE corrections) and IGMH analysis ($\delta_{g\text{-inter}}$ = 0.005 a. u.) of (**a**) *DPCA@PVA* and (**b**) *DPCA-KI₃@PVA* model, respectively. (**c**) Distribution of different interactions between *PVA* and *DPCA-KI₃* by IGMH scatter map. (**d**) Electron-hole analysis, SOC constants, and energy levels of the S_1 and T_1 states for *DPCA*, *DPCA@PVA*, *DPCA-KI₃*, and *DPCA-KI₃@PVA* models based on their S_0 optimized geometry.

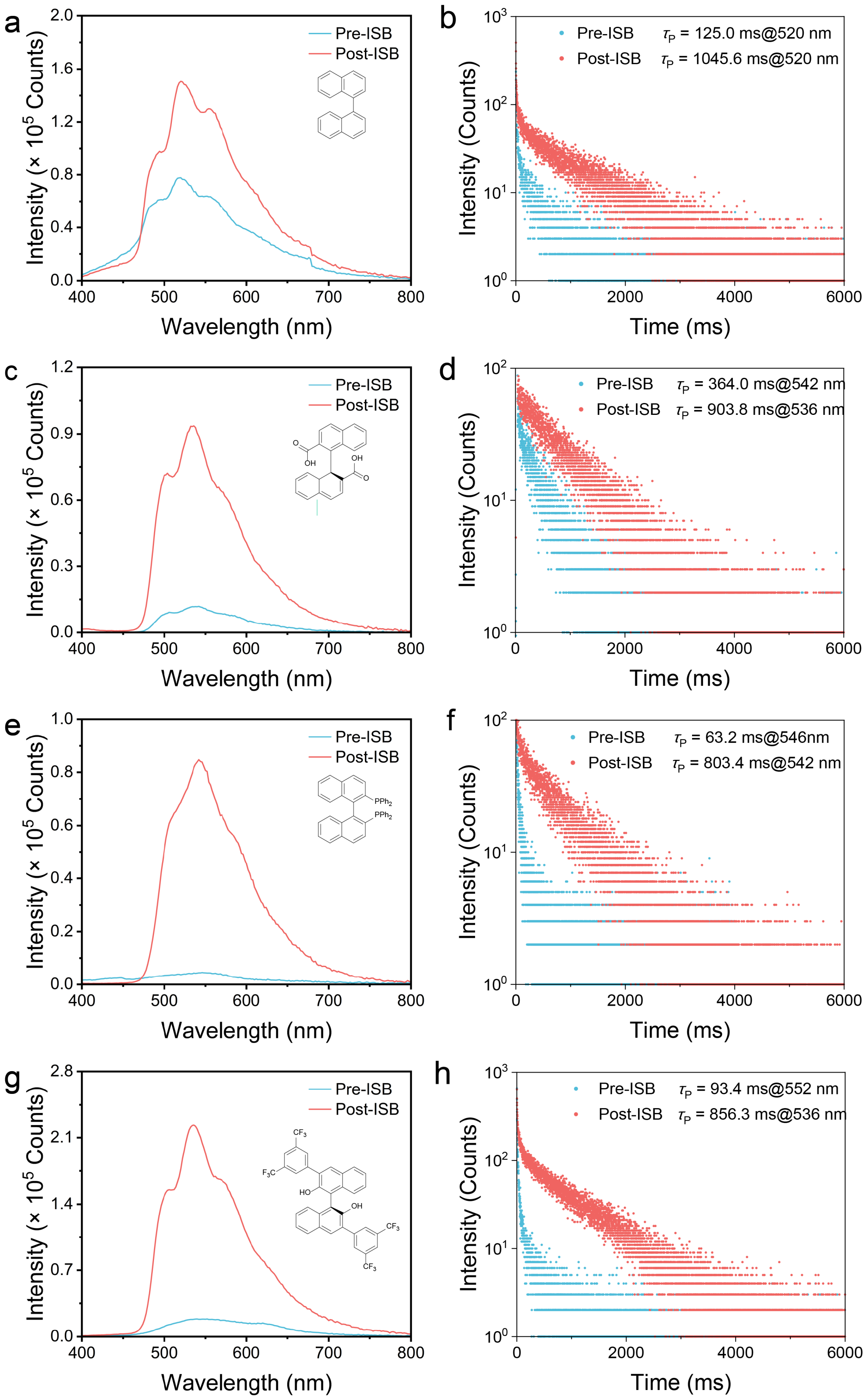

Supplementary Figure 41. RTP changes. (**a-h**) Versatility of ISB treatment in enhancing RTP, the excitation wavelength is 254 nm.

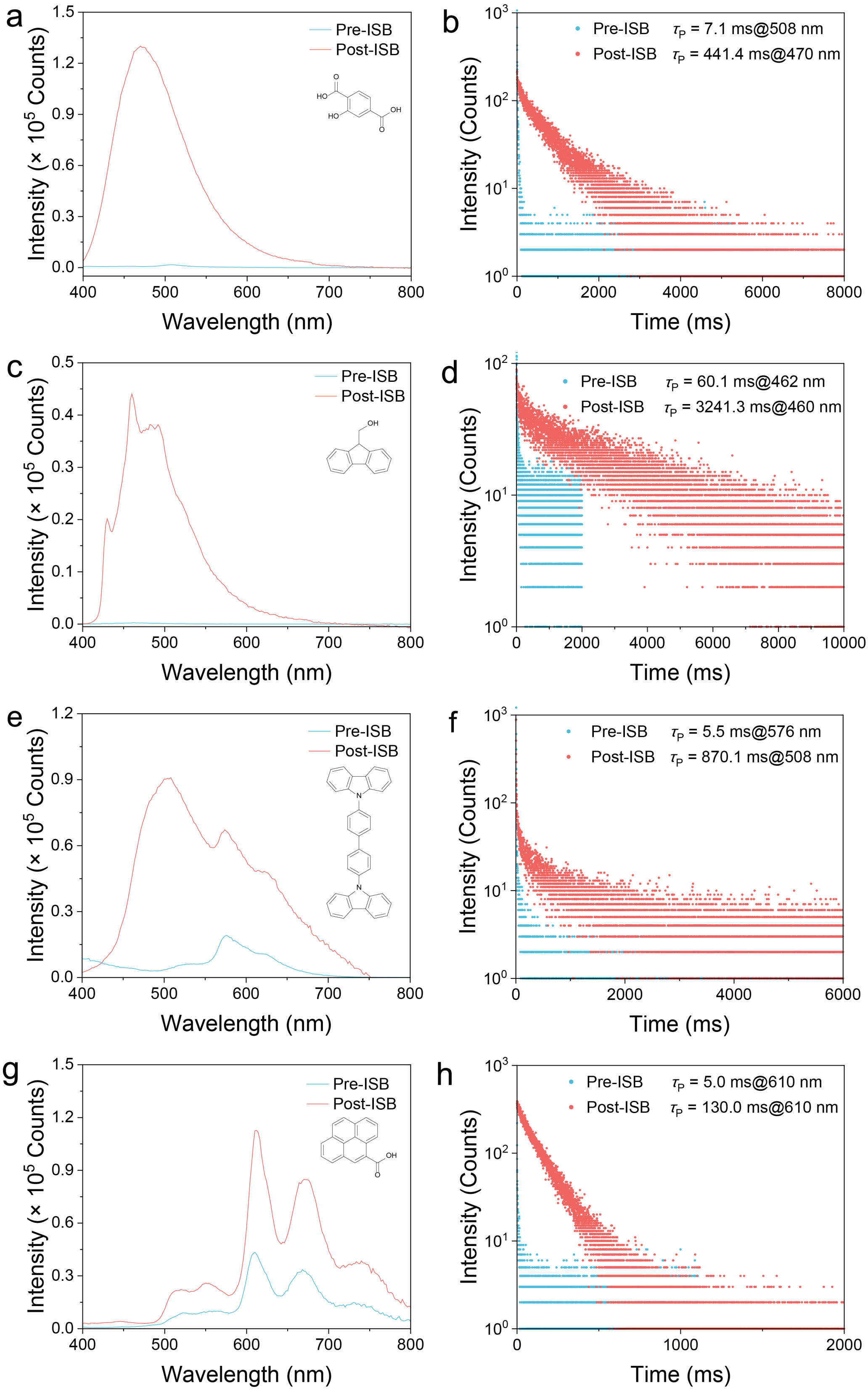

Supplementary Figure 42. RTP changes. (**a-h**) Versatility of ISB treatment in enhancing RTP, the excitation wavelength is 254 nm.

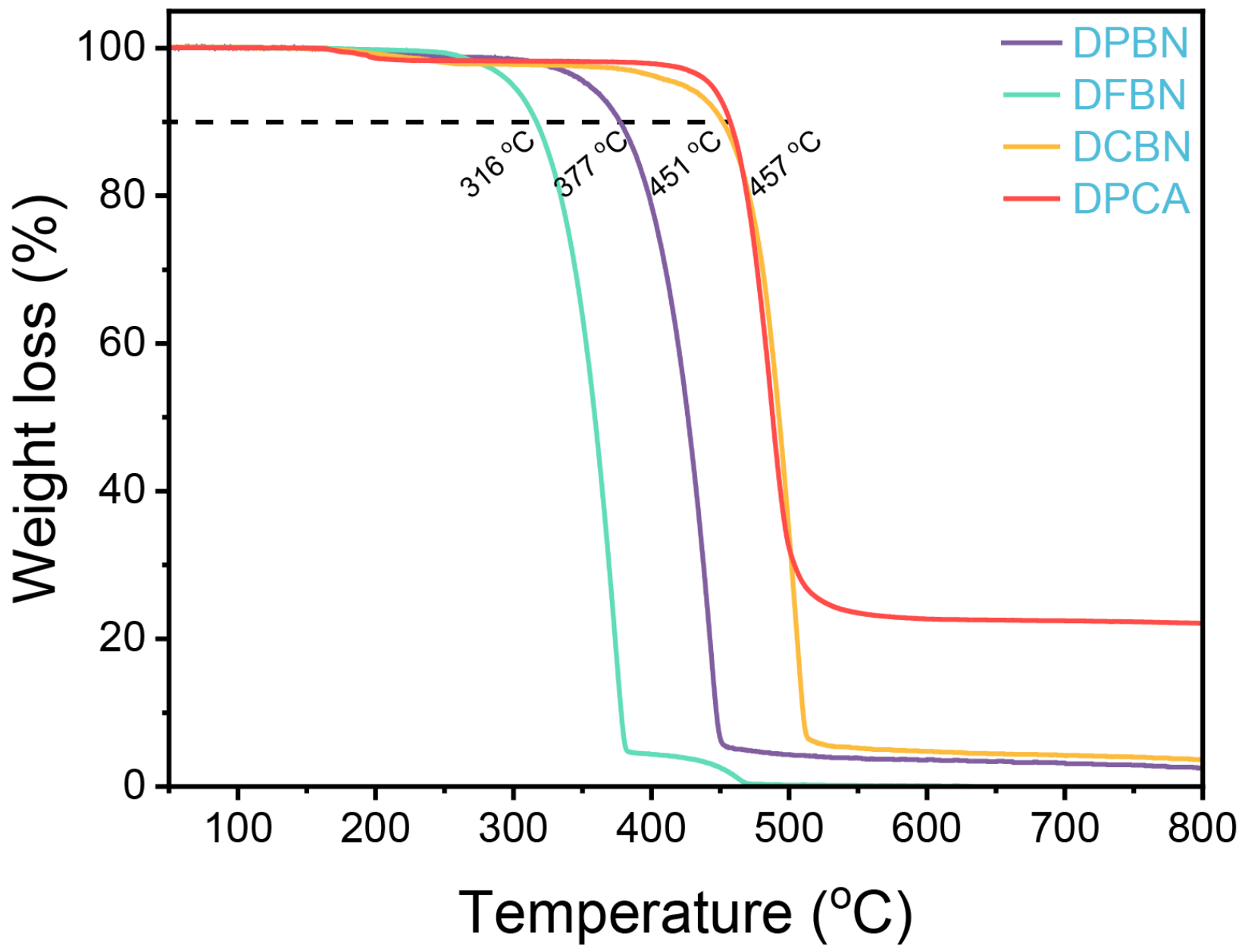

Supplementary Figure 43. TGA curves of the four *BINs* molecules.

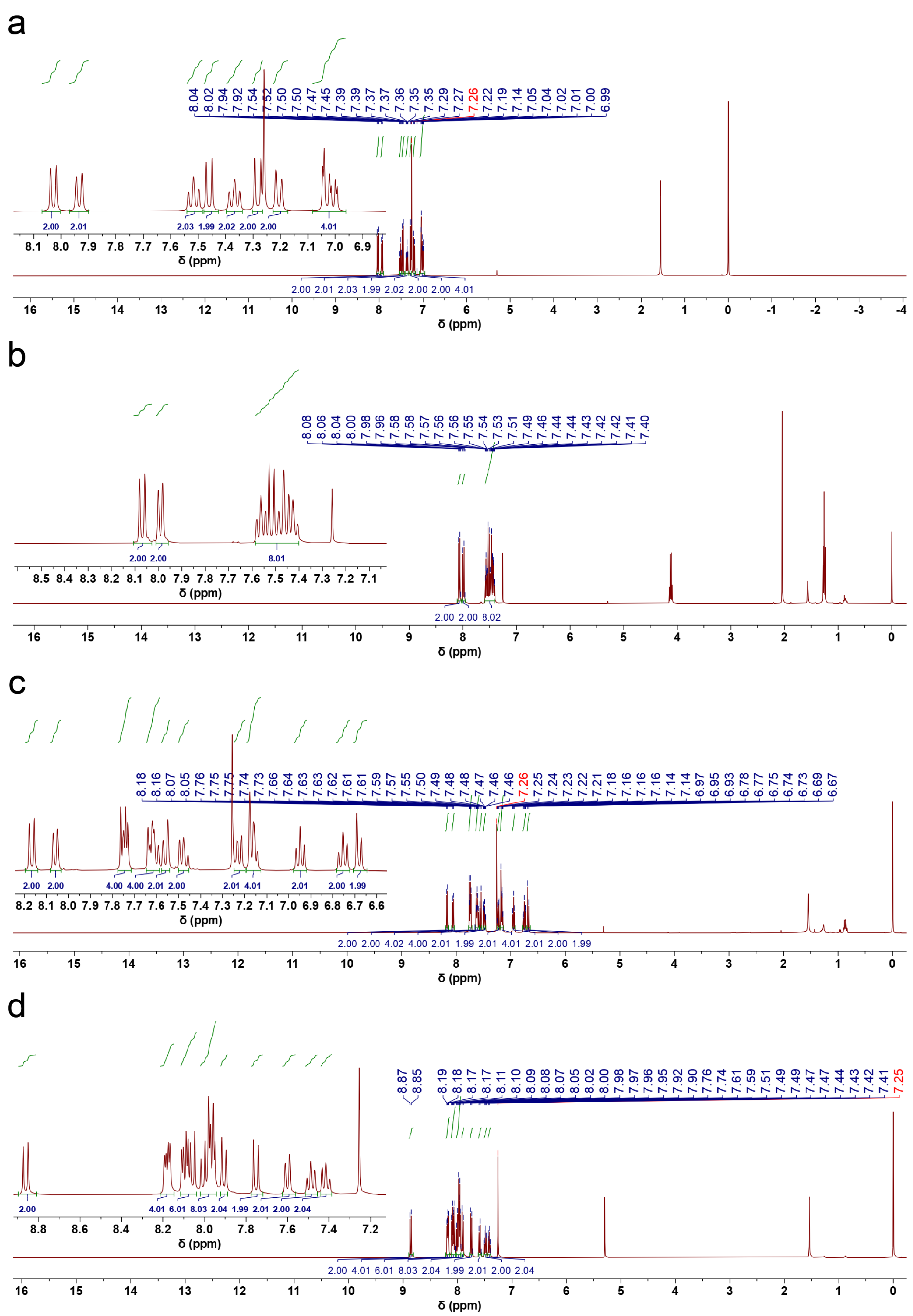

Supplementary Figure 44. ^{1}**H NMR spectra. (a)** *DPBN*, **(b)** *DFBN*, **(c)** *DCBN* and **(d)** DPCA. Note: According to the ICP-MS results (Supplementary Table 2 and 3), *boric acid*, *BINs*, and iodine were dissolved in deuterated chloroform at a molar ratio of 50:1:0.1, and subsequently heated at 70 °C for 2 min prior to ^{1}H NMR characterization in order to simulate the ISB treatment conditions.

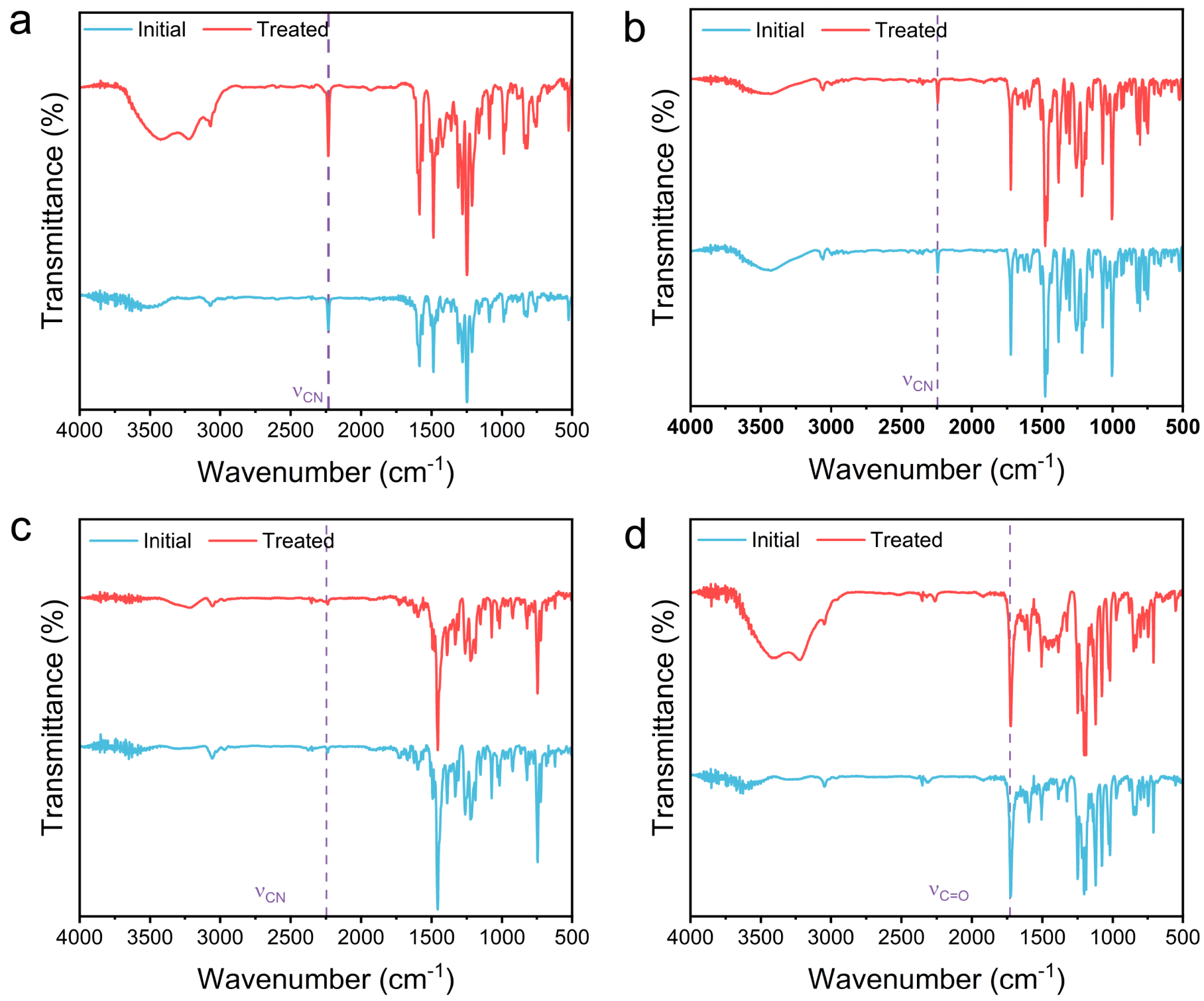

Supplementary Figure 45. FT-IR spectra. (**a**) *DPBN*, (**b**) *DFBN*, (**c**) *DCBN*, and (**d**) *DPCA* powder in the initial state and after treatment. Note: According to the ICP-MS results (Supplementary Table 2 and 3), *boric acid*, *BINs*, and *iodine* were mixed at a molar ratio of 50:1:0.1, thoroughly ground to ensure homogeneity before FT-IR tests. There are characteristic absorption bands at ~2230 cm^{-1}, attributable to the C≡N stretching vibrations of *DPBN*, *DFBN* and *DCBN*, together with a distinct band at ~1720 cm^{-1}, arising from the C=O stretching vibrations. Collectively, these results confirm that the weak oxidative nature of *iodine* does not lead to detectable structural damage to the *BINs*.

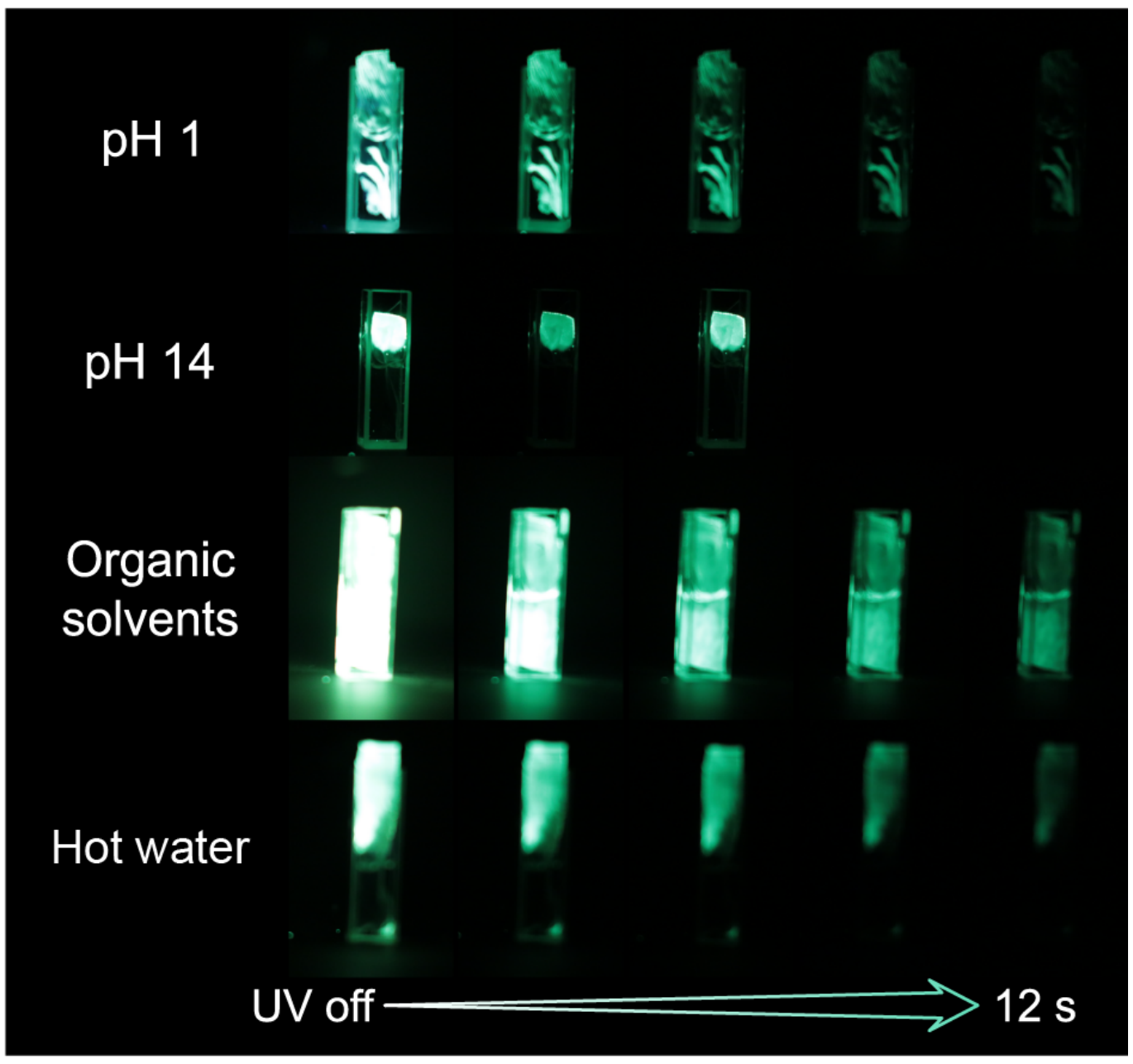

Supplementary Figure 46. Afterglow photos of *DFBN@PVA* after immersed into harsh solvents after 12 hours.

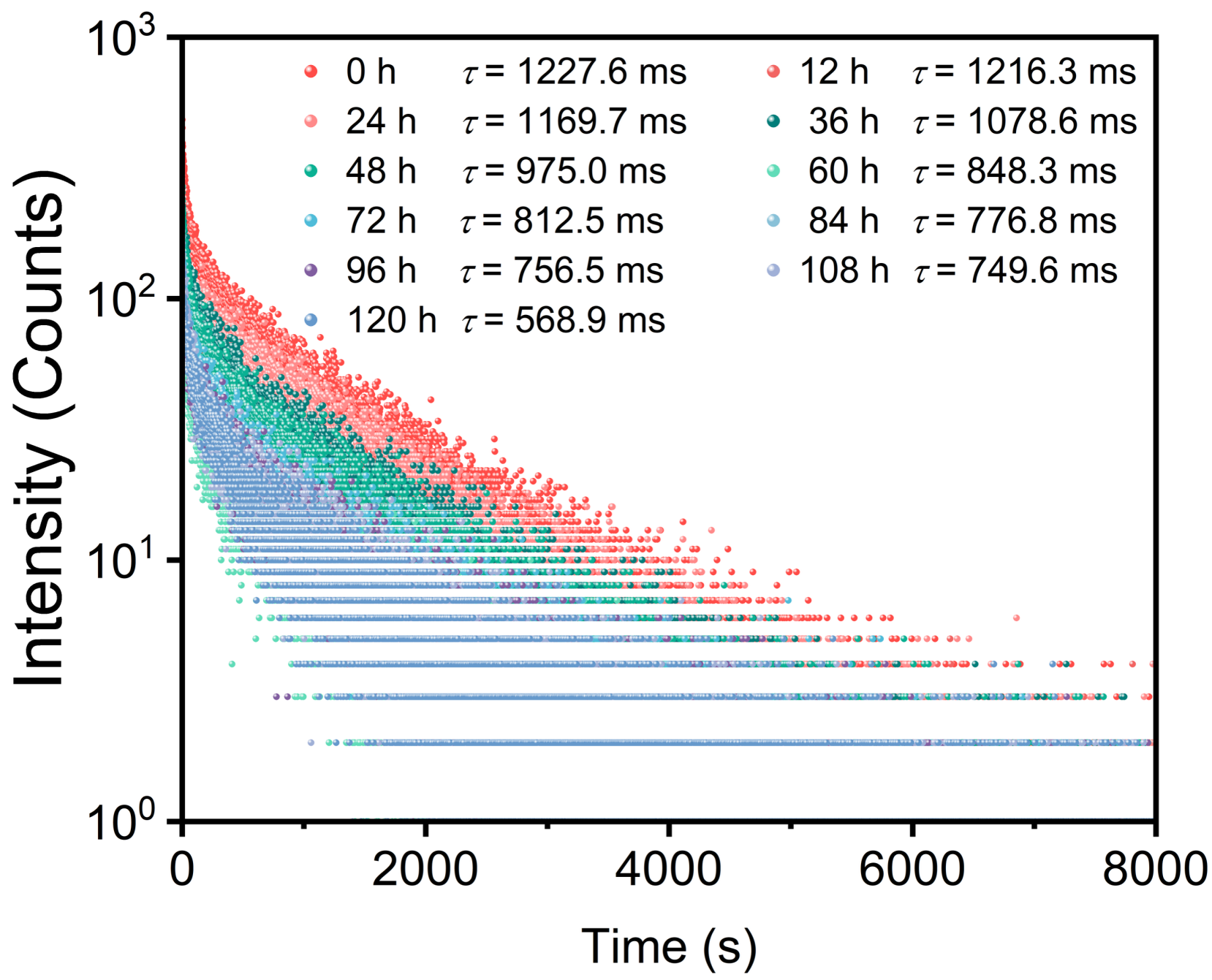

Supplementary Figure 47. Lifetime decay curves (λ_{em} = 528 nm) of the post-ISB *DFBN@PVA* film immersed underwater.

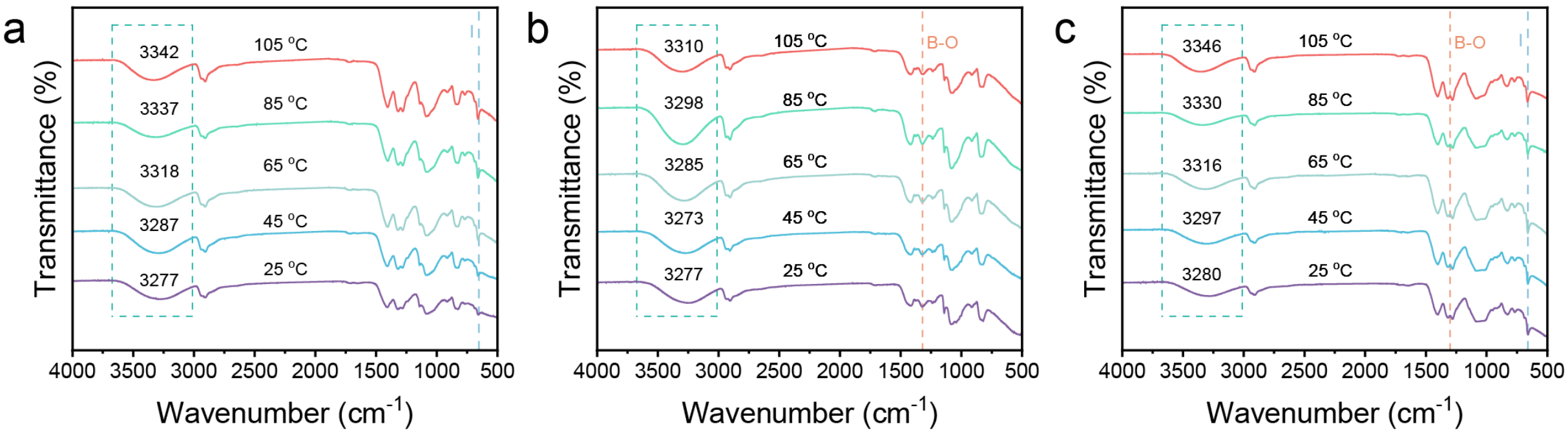

Supplementary Figure 48. **Temperature-dependent in-situ FT-IR spectra.** (**a**) iodine-treated *DFBN@PVA* film, (**b**) boron-treated *DFBN@PVA* film, and (**c**) ISB-treated *DFBN@PVA* film with the heating rate of 2 °C min^{-1}.

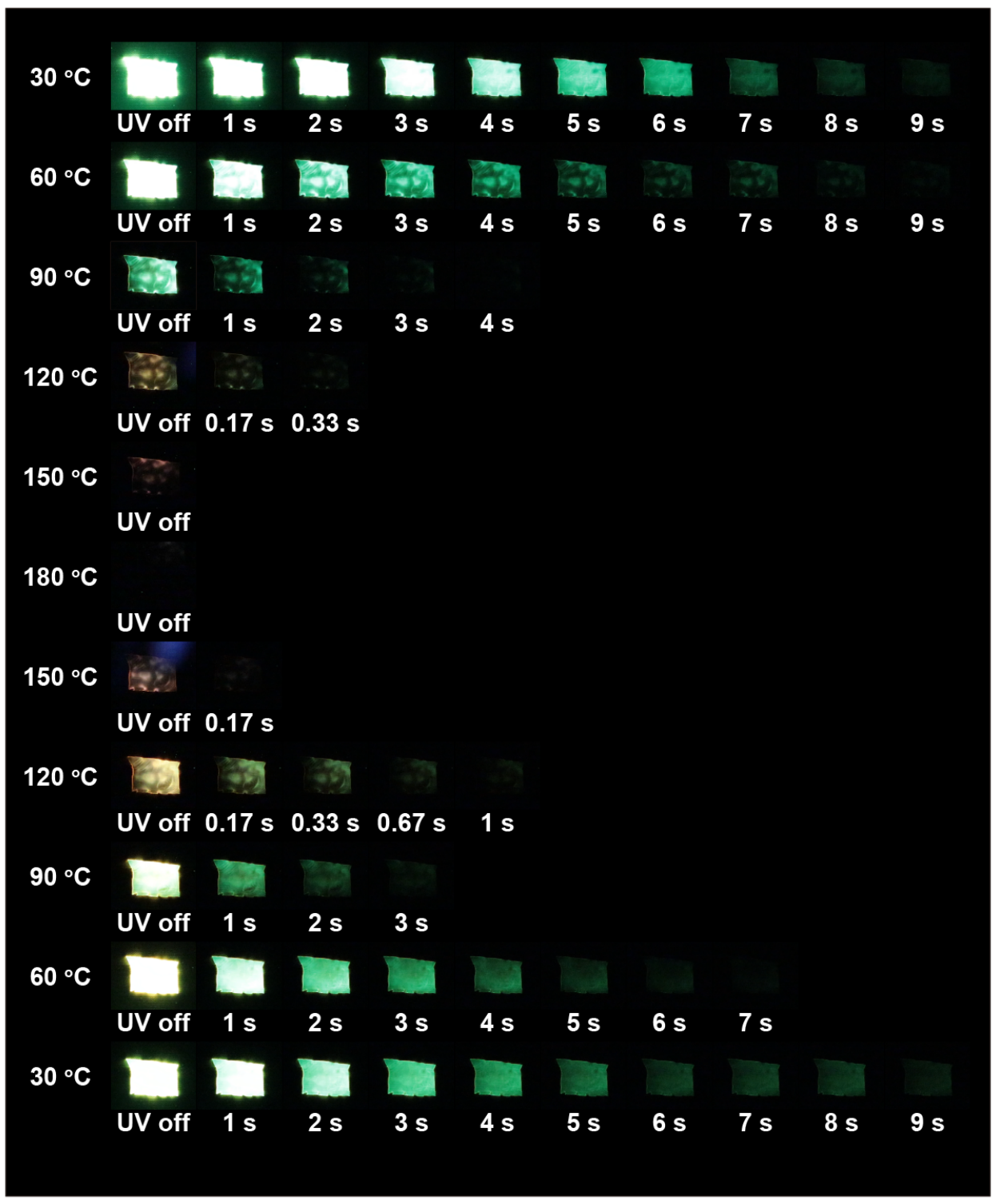

Supplementary Figure 49. Afterglow photographs of post-ISB *DFBN@PVA* film subjected to a complete heating-cooling cycle. Notes: The post-ISB *DFBN@PVA* film was placed on a hot plate and heated from 30 °C to 180 °C in 30 °C increments. At each temperature, the sample was equilibrated for 2 min before its afterglow image was acquired under 365 nm UV excitation. During cooling, the heating was turned off. Once the film reached a target temperature, it was reheated and allowed to stabilize for 2 min prior to capturing afterglow images under the same excitation conditions.

Supplementary Table 1. Kinetic calculations and photophysical rate constants of pre-ISB and post-ISB treated *BINs@PVA* films.

Sample		Φ_{PL} (%)	Prompt photoluminescence τ_F ns	Φ_F (%)	k_r^F (s^{-1})	k_{ISC} (s^{-1})	Delayed photoluminescence τ_P (ms)	Φ_P (%)	k_r^P (s^{-1})	k_{nr}^P (s^{-1})
DPBN@PVA	Pre-ISB	26.5	4.3	39.3	9.14×10^7	1.41×10^8	944.4	4.7	0.08	0.98
	Post-ISB	26.5	5.2	12.3	2.37×10^7	1.69×10^8	1690.3	14.2	0.1	0.50
DFBN@PVA	Pre-ISB	11.6	7.6	10.2	1.34×10^7	1.18×10^8	43.7	1.4	0.37	22.53
	Post-ISB	34.5	8.0	0.7	8.75×10^5	1.24×10^8	1228.4	33.8	0.28	0.54
DCBN@PVA	Pre-ISB	2.1	3.8	1.2	3.16×10^6	2.60×10^8	47	0.9	0.19	21.09
	Post-ISB	7.0	10.4	1.9	1.83×10^6	9.43×10^7	293.6	5.1	0.18	3.23
DPCA@PVA	Pre-ISB	23.1	27.2	22.6	8.31×10^6	2.85×10^7	30	0.5	0.22	33.12
	Post-ISB	22.9	29.7	18.5	6.23×10^6	2.74×10^7	205.6	4.4	0.26	4.60

Note: τ_F and τ_P data are originated from Figure 2f and Supplementary Figure 19, absolute Φ_F and Φ_P data are from Supplementary Figure 22.

Supplementary Table 2. Boron content in the *BINs@PVA* films.

Sample	Sample weight (g)	Final volume (ml)	Dilution factor	B Concentration (μmol/g)	Molar ratio ($n_{Phosphor}$:n_B)
DCBN	0.0237	15	1500	1647767229	0.02
DFBN	0.0404	15	1500	1232466997	0.02
DPBN	0.0323	15	1500	842621259	0.02
DPCA	0.0419	15	1500	914777247	0.03

Supplementary Table 3. Iodine content in the *BINs@PVA* films.

Sample	Sample weight (g)	Final volume (ml)	Dilution factor	I Concentration (μmol/g)	Molar ratio ($n_{Phosphor}$:n_I)
DCBN	0.0258	10	100	361634	82.82
DFBN	0.0431	10	100	942152	26.44
DPBN	0.0319	10	100	4698982	4.39
DPCA	0.0527	10	100	2260292	13.25

Note: The concentrations (c_ω) of iodine and boron in *BINs@PVA* films were quantified by ICP-MS. The *BIN* concentration in the precursor solution was 0.5 or 1.0 mg mL^{-1} (Supplementary Figure 17), while the *PVA* concentration was fixed at 45 mg mL^{-1}. Accordingly, the *BIN* loading in the resulting films (c_ω) was estimated:

For *DPBN* and *DFBN*: $$c_\omega = \frac{1000}{90}\ g/kg = \frac{10^9}{90}\ \mu g/kg$$

For *DCBN* and *DPCA*: $$c_\omega = \frac{1000}{45}\ g/kg = \frac{10^9}{45}\ \mu g/kg$$

ICP-MS analysis indicates that the molecular weights of *DPBN*, *DFBN*, *DCBN* and *DPCA* are 539.1 g mol^{-1}, 446.1 g mol^{-1}, 741.2 g mol^{-1} and 742.2 g mol^{-1}, respectively. Based on these values, the molar ratio can be calculated.

Supplementary Table 4. Test parameters of ICP-MS.

Parameter	Value
Sample depth	5 mm
RF power	1550 W
Integration	0.1 s
Nebulizer chamber temperature	2.5 °C
Integrating time	0.1 s
Plasma gas flow	15 L/min
Auxiliary gas flow	0.78 L/min
Detection mode	KED

Note: Owing to the manually operated nature of the ISB process and the intrinsic variation in the loading capacities of different *BINs@PVA* films toward boron and iodine species, discrepancies in elemental contents were observed among the four films. Nevertheless, it can be reliably concluded that variations within this range exert a negligible influence on the phosphorescence performance of the *BINs@PVA* films. This parameter set was consistently identified through dozens of repeated trials during both the exploratory and characterization stages, demonstrating satisfactory reproducibility and reliability.
