# Supplementary material for: Coordinatively improving polymeric phosphorescence lifetime and quantum yield via triplet exciton modulation
Source: Nat Commun. 2026 Jun 11;17:8890. doi: 10.1038/s41467-026-74266-8 (PMC13500660; doi:10.1038/s41467-026-74266-8)
Supplement: Supplementary file 2 [file 41467_2026_74266_MOESM2_ESM.pdf]

Description of Additional Supplementary Files

Supplementary Data 1. Atomic coordinates of the optimized computational models.

Supplementary Movie 1. Afterglow of *BINs* in CH_2Cl_2 (10^{-4} mol/L) at 77 K.

Supplementary Movie 2. Afterglow of *BINs@PVA* at room temperature. *DPBN* film was excited with a 254 nm UV lamp, while *DFBN*, *DCBN*, and *DPCA* were excited with a 365 nm UV lamp.
