# Supplementary material for: Coordinatively improving polymeric phosphorescence lifetime and quantum yield via triplet exciton modulation
Source: Nat Commun. 2026 Jun 11;17:8890. doi: 10.1038/s41467-026-74266-8 (PMC13500660; doi:10.1038/s41467-026-74266-8)
Supplement: Supplementary file 3 [file 41467_2026_74266_MOESM6_ESM.pdf]

nature portfolio

Peer Review File

Coordinatively Improving Polymeric Phosphorescence Lifetime and Quantum Yield via Triplet Excitons Modulation

Corresponding Author: Professor Yanli Zhao

Parts of this Peer Review File have been redacted as indicated to remove third-party material.

This file contains all reviewer reports in order by version, followed by all author rebuttals in order by version.

Version 0:

Reviewer comments:

Reviewer #1

(Remarks to the Author)
The manuscript presents a synergistic strategy in which the authors ingeniously harness the strong complexation ability of PVA with iodine ions to form polyiodide species (I_3^- and I_5^-). This clever design effectively introduces a heavy-atom effect, leading to a pronounced enhancement in the RTP performance of the DFBN@PVA film. Through this composite structure, the authors successfully achieve simultaneous optimization of the non-radiative rate constant and the intersystem crossing rate constant for phosphorescence emission. The phosphorescence of the film remains remarkably stable after immersion in water for five days or in hot water for ten minutes overnight. Moreover, the outstanding thermal and water stability of the PVA-based RTP materials is impressive and rarely reported. Overall, this work is highly innovative and provides a valuable contribution to the design of robust and efficient RTP materials.
In this sense, the reviewer suggests that this work can be accepted after minor revisions addressing the following concerns.
1. The authors report that the DFBN@PVA films maintain favorable phosphorescence emission even in 100 °C water and provide in-situ FT-IR results confirming that the film structure remains intact at elevated temperatures. The reviewer suggests that the authors supply additional data on the high-temperature phosphorescence, such as determining the maximum temperature at which afterglow emission is observed, and include intuitive photographic evidence to illustrate this phenomenon.
2. The ISB strategy is proposed to induce both PVA crystal transformation and the coordination of I_3^- and I_5^-. Which structural change primarily governs the exceptional phosphorescent properties of the DFBN@PVA films? Is it the coordination effect of iodine, the PVA crystal transformation, the cross-linking by boric acid, or a synergistic effect among them?
3. Based on Question 2, the reviewer recommends that the authors provide a more detailed discussion in the manuscript to clarify and highlight the superiority of the synergistic ISB strategy.
4. Figure 4d indicates that the post-ISB samples exhibit higher crystallinity. However, the authors also state that “the incorporation of iodine disrupts the PVA crystalline order” in these samples. The authors are encouraged to discuss this apparent contradiction.
5. Figures 4e and 4f show that the 2D scattering patterns of the post-ISB samples are circular, indicative of isotropic crystal planes and crystal orientations in the DFBN@PVA films. This observation seems contrary to the stretching-induced orientation of PVA chains illustrated in Figure 1 and the SEM result in Figure 4g. The authors should clarify this inconsistency.
6. If high crystallinity indeed facilitates the formation of I_3^- and I_5^- coordination structures, thereby enhancing both τ_p and Φ_p, it is suggested that the schematic in Figure 1 incorporate the roles of PVA crystallinity and polyiodide coordination. However, the reviewer leaves it to the authors’ discretion to decide whether this addition is necessary.

Reviewer #2

(Remarks to the Author)
In this work, the authors prepared a series of PVA-based afterglow materials using an Iodine-Stretch-Boric Acid (ISB) strategy and systematically characterized their fundamental properties and photophysical performance. They claim that the ISB strategy not only enhances the rigidity of PVA but also significantly boosts phosphorescence through the external

heavy-atom effect. While it is well established that both matrix rigidification and the external heavy-atom effect can enhance phosphorescence, strategies that synergistically combine these two effects to achieve substantial performance improvements are relatively rare. Therefore, I acknowledge that this work possesses a certain degree of novelty. However, regrettably, the data presented do not adequately support the authors' conclusions. In particular, the reported phosphorescence quantum yields are provided only as numerical values without supporting evidence or detailed methodology, severely undermining their credibility. The same issue applies to the non-radiative decay rates. Considering the concerns outlined below, I believe the overall quality of the manuscript does not meet the standards expected for Nature Communications. Therefore, I recommend rejection.

Specific Comments:
1. Steady-state emission spectra should be provided. These spectra are essential for understanding the luminescent behavior of the system, particularly the relative contributions of fluorescence and phosphorescence.
2. To the best of our knowledge, absolute photoluminescence quantum yield instruments measure steady-state emission and lack time-gating capability. Thus, accurate determination of phosphorescence quantum yield remains a significant challenge. Since phosphorescence quantum yield is a key metric for evaluating the effectiveness of the ISB strategy, the authors must disclose the exact methodology used. Given the severe spectral overlap between fluorescence and phosphorescence in this system (Fig. S13), the authors should record full decay curves and calculate fluorescence and phosphorescence quantum yields based on the integrated areas of the fast (fluorescent) and slow (phosphorescent) decay components.
3. In Table S1, why does the fluorescence lifetime increase after ISB treatment? Take DPBN as an example: its fluorescence quantum yield drops sharply from 39.3% to 12.3%, while its phosphorescence quantum yield rises from 4.7% to 14.2%. This strongly suggests that the heavy atom effect significantly enhances ISC from S_1 to T_1. Under such circumstances, the fluorescence lifetime should decrease due to accelerated S_1 decay.
4. How were the rate constants Kisc, KnrP, KnrF calculated? Were they derived from Φ/τ? If so, please provide the calculation procedures and raw data for key parameters such as Φisc，ΦnrF，ΦnrP. Moreover, we recommend using transient absorption spectroscopy to directly determine KnrF and KISC; otherwise, the analysis risks circular reasoning.
5. Why were 77 K photophysical measurements performed in DCM? Standard practice uses glass-forming solvents such as 2-methyltetrahydrofuran.
6. The TA data (Fig. S24) fail to explain why BINs show no afterglow at 77 K but exhibit dramatically enhanced afterglow after BA treatment. The authors should focus on tracking S_1 decay dynamics and triplet formation before and after ISB treatment. Additionally, the TA data presentation is extremely poor. Fig. S24 is nearly unreadable. Notably, Fig. S24e reports a fitted lifetime of 127.2 ns, which is clearly unreasonable given the instrument's time window of only 7 ns.
7. What are the actual iodine and boron contents in the films after ISB treatment? Elemental analysis would help clarify this.
8. TGA results suggest that both untreated and ISB-treated films contain significant amounts of water. Optimizing the preparation protocol to reduce moisture content could further enhance material performance.
9. In Fig. 5d, please describe in detail how the computational model was constructed, especially how the position of I_3^- was determined.
10. All reported 1H NMR spectra are inadequate. Full spectra, not just downfield regions, must be shown.
11. Did BINs undergo degradation during ISB treatment? Please provide stability or decomposition tests to address this concern.

Reviewer #3

(Remarks to the Author)
In this manuscript, the authors report that the phosphorescence properties of organic molecules are significantly improved by "ISB" treatment, defined as the stretching of PVA films followed by impregnation with aqueous iodine and boric acid solutions. Experimental and theoretical analyses revealed that the ISB treatment enhances the RTP properties of the dyes through the external heavy atom effect and the orientation of PVA chains. Since ISB treatment is a simple method widely used in the production of polarizing plates, the discovery that such treatment leads to improved phosphorescence properties is highly interesting. Therefore, I recommend the acceptance of this manuscript after minor revisions.
Specific Comments
1. The authors label PL spectra as "fluorescence spectra" or "phosphorescence spectra." However, in several instances, both components are clearly observed in the data, making these labels technically inaccurate. The authors should adopt more precise terminology, such as "steady-state PL spectra" or "time-gated PL spectra," to accurately reflect the measurement conditions.
2. Please specify the monitored wavelengths for the PL decay curves presented in the manuscript.
3. Please provide a detailed description in the Experimental section regarding how the fluorescence and phosphorescence quantum yields (Φ_F and Φ_P) were determined. Additionally, please clarify the calculation methods used to derive the rate constants.

Version 1:

Reviewer comments:

Reviewer #1

(Remarks to the Author)

The authors have addressed all concerns. The manuscript is now recommended for publication in Nature Communications.

Reviewer #3

(Remarks to the Author)
Although the authors have generally addressed the comments, several points require further clarification or action:
1. The authors refer to the steady-state PL spectra in Figure S18 as "prompt spectra"; however, this term does not accurately signify steady-state conditions. It must be clarified whether the spectra in Figure S18 represent time-gated PL spectra (where long-lived components have been excluded) or steady-state spectra obtained under continuous excitation.
2. Experimental Section: In calculating the rate constants, the authors derived the subsequent equations under the assumption that kPnr is sufficiently smaller than kPr. However, as shown in Supplementary Table 1, the calculated kPnr is significantly larger than kPr, directly contradicting this premise. Furthermore, as shown in Figure 23, both the phosphorescence intensity and lifetime exhibit clear temperature dependence. This indicates that kPnr is substantial even at room temperature; therefore, neglecting it in the derivation of the equations is not justifiable. Also, rate constants should be denoted by a lowercase "k" rather than an uppercase "K".

In addition to the points above, I have reviewed the authors' responses to Reviewer 2. Overall, I believe the authors have addressed Reviewer 2's comments appropriately. However, a persistent concern remains regarding whether the authors are correctly distinguishing between steady-state and time-resolved data. The following points must be addressed in a revised version:
1. As noted in my previous comments, the "Prompt spectra" shown in Figure S18 do not generally represent steady-state spectra. The manuscript must clearly state in the Experimental Section which specific values were measured and provide the exact methodology used for those measurements. Furthermore, for all time-gated spectra, the specific gate width (time window) must be clearly defined.
2. While the revised Experimental Section suggests that ΦF and ΦP were determined through direct measurement, it remains unclear how ΦF was specifically isolated. Figure S21 appears to show ΦPL, which is fundamentally different from ΦF. The authors must clarify this distinction and the calculation process.
3. Measuring the absolute ΦP using a time-gating method is technically demanding. Was the calibration for the excitation pulse intensity performed correctly to ensure absolute values? The authors should provide the detailed calculation formulas in the Experimental Section, explaining how photons emitted outside the gate window were integrated and normalized to obtain the absolute ΦP.
4. Please clarify whether the Φ values used to calculate the photophysical rate constants (kISC, kr, knr) in Table S1 and throughout the text refer to ΦPL or ΦF. It is essential to re-verify the mathematical consistency of these calculations based on the correct definitions of each quantum yield.

Version 2:

Reviewer comments:

Reviewer #3

(Remarks to the Author)
The authors have appropriately addressed the reviewers' concerns. However, one correction is required prior to acceptance. While the authors calculate ΦISC = 1 – ΦF on the grounds that the S0–S1 energy gaps are sufficiently large, the cited literature specifically targets simple aromatic hydrocarbons, and this theory is not universally applicable to all types of luminophores. Therefore, the description in the Experimental Section must be revised to clarify that this theory was originally proposed for aromatic hydrocarbons and that the authors are assuming its validity for their specific system in order to derive these values.

Open Access This Peer Review File is licensed under a Creative Commons Attribution 4.0 International License, which permits use, sharing, adaptation, distribution and reproduction in any medium or format, as long as you give appropriate credit to the original author(s) and the source, provide a link to the Creative Commons license, and indicate if changes were made.
In cases where reviewers are anonymous, credit should be given to 'Anonymous Referee' and the source.
The images or other third party material in this Peer Review File are included in the article's Creative Commons license, unless indicated otherwise in a credit line to the material. If material is not included in the article's Creative Commons license and your intended use is not permitted by statutory regulation or exceeds the permitted use, you will need to obtain permission directly from the copyright holder.
To view a copy of this license, visit https://creativecommons.org/licenses/by/4.0/

Response Letter

Reviewer #1 (Remarks to the Author):

The manuscript presents a synergistic strategy in which the authors ingeniously harness the strong complexation ability of PVA with iodine ions to form polyiodide species (I_3^- and I_5^-). This clever design effectively introduces a heavy-atom effect, leading to a pronounced enhancement in the RTP performance of the DFBN@PVA film. Through this composite structure, the authors successfully achieve simultaneous optimization of the non-radiative rate constant and the intersystem crossing rate constant for phosphorescence emission. The phosphorescence of the film remains remarkably stable after immersion in water for five days or in hot water for ten minutes overnight. Moreover, the outstanding thermal and water stability of the PVA-based RTP materials is impressive and rarely reported. Overall, this work is highly innovative and provides a valuable contribution to the design of robust and efficient RTP materials.

In this sense, the reviewer suggests that this work can be accepted after minor revisions addressing the following concerns.

Response: We cordially appreciate the reviewer's suggestions. We have carefully revised the manuscript according to the comments.

1. The authors report that the DFBN@PVA films maintain favorable phosphorescence emission even in 100 °C water and provide in-situ FT-IR results confirming that the film structure remains intact at elevated temperatures. The reviewer suggests that the authors supply additional data on the high-temperature phosphorescence, such as determining the maximum temperature at which afterglow emission is observed, and include intuitive photographic evidence to illustrate this phenomenon.

Response: Many thanks for the useful suggestion. According to your advice, we have provided additional afterglow photographs of post-ISB DFBN@PVA film to investigate the maximum temperature at which they emit phosphorescence. The relevant experiments are shown below and have been incorporated into the "Applications to harsh environmental conditions" section of the revised Manuscript: "Throughout a full heating-cooling cycle, the post-ISB DFBN@PVA film retained discernible afterglow even at temperatures exceeding 150 °C (Fig. R1), and its emission performance was almost fully restored to the initial state after the cycle, representing a rare example among polymer-based RTP materials."

We note that the reviewers may perceive an inconsistency between the afterglow images obtained in hot water (Fig. S45) and those recorded at 90 °C (Fig. S48). This discrepancy was not resulted from any data error, but from differences in the camera acquisition settings. Specifically, the exposure parameters in Fig. S45 were adjusted so that the image appeared slightly overexposed at 90 °C, whereas those in Fig. S48 were calibrated with reference to slight overexposure observed at 30 °C.

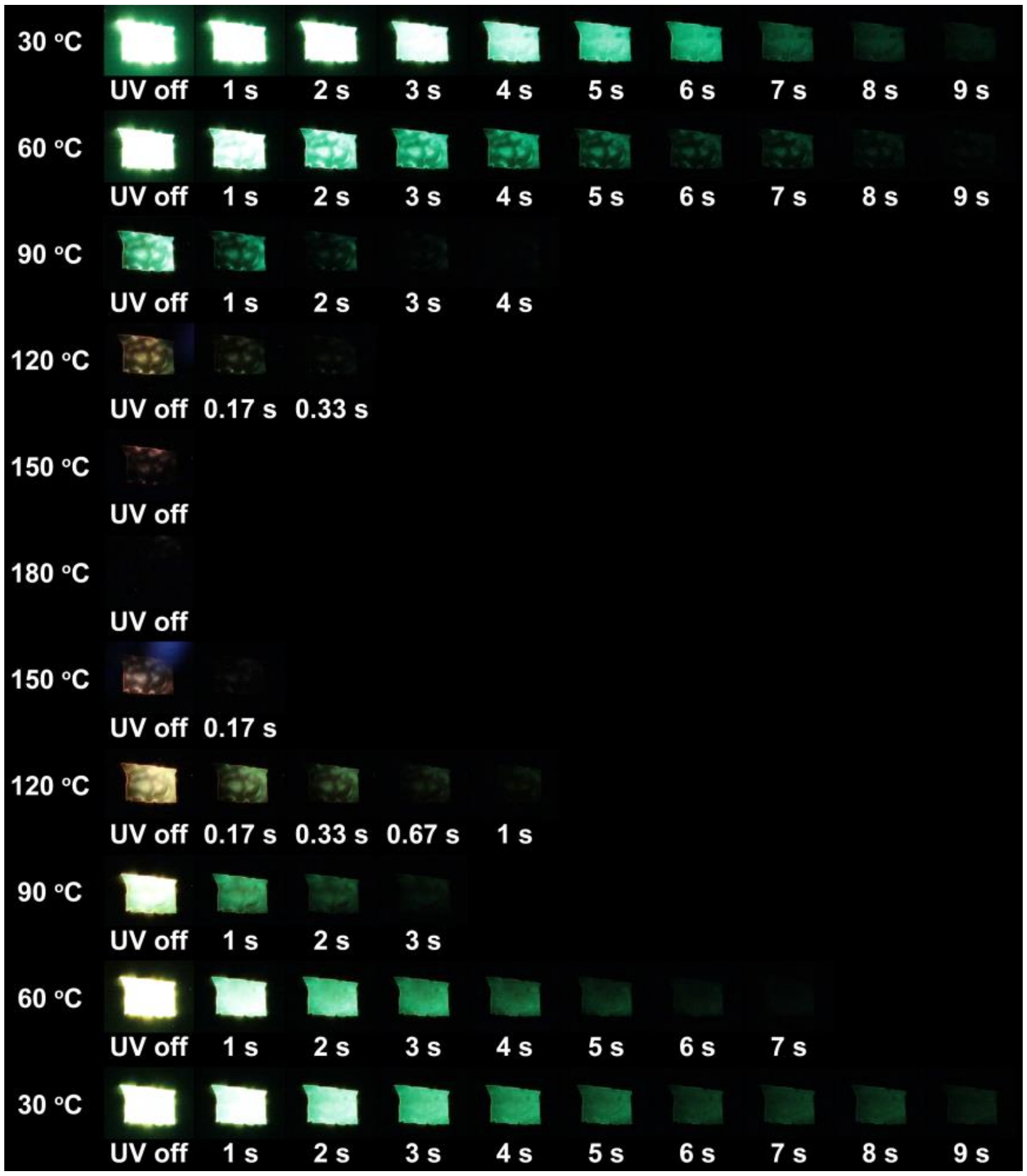

Fig. R1 (Fig. S48). Afterglow photographs of post-ISB DFBN@PVA film subjected to a complete heating-cooling cycle. Notes: The post-ISB DFBN@PVA film was placed on a hot plate and heated from 30 °C to 180 °C in 30 °C increments. At each temperature, the sample was equilibrated for 2 min before its afterglow image was acquired under 365 nm UV excitation. During cooling, the heating was turned off. Once the film reached a target temperature, it was reheated and allowed to stabilize for 2 min prior to capturing afterglow images under the same excitation conditions.

2. The ISB strategy is proposed to induce both PVA crystal transformation and the coordination of I_3^- and I_5^-. Which structural change primarily governs the exceptional phosphorescent properties of the DFBN@PVA films? Is it the coordination effect of iodine, the PVA crystal transformation, the cross-linking by boric acid, or a synergistic effect among them?

Response: Thanks for your meticulous review and the valuable guidance. After establishing the optimal ISB treatment conditions (Fig. S17), we have first verified the successful incorporation of iodine using FT-IR spectroscopy (Fig. 4a and S27). Raman spectroscopy further confirmed that iodine predominantly exists in the forms of I_5^- and I_3^- ions (Fig. 4b). As described in the preparation of post-ISB BINs@PVA films, the iodide concentration was estimated to be approximately 0.05 M. This value is consistent with the Raman spectral features reported for PVA films soaked in low-

concentration I_2/KI solutions in the literature (Fig. R2). Accordingly, we hypothesize that both I_5^- and I_3^- ions are efficient in enhancing the RTP performance of BINs films, and the relevant conclusions have been refined in the revised manuscript.

In addition, we have calculated the SOC constants between S_1 and T_1 states by TD-DFT methods based on different iodine model (I^-, I_2, I_3^-, and I_5^-) in DFBN (Fig. R3). Although the incorporation of iodine enhanced the ISC process, DFBN-KI_3 showed the largest SOC constants rather than DFBN-KI_5. It is possible that an increase concentration of I_5^- ions would improve RTP performance because of a stronger heavy-atom effect (HAE). In practice, however, higher iodide concentrations or extended iodine treatment times led to completely black films, caused by excessive polyiodide formation and broad visible absorption across the spectra.

According to Figs. 3a, 3b and 3d, we have confirmed that iodine soaking, stretch-induced crystallization, and boric acid treatment are all beneficial to the RTP performance. Furthermore, we have concluded that it is the synergistic effects of the three factors that endow BIN films with excellent RTP performance and the potential for applications under harsh environmental conditions. Specifically, iodine facilitates the ISC process via the HAE (Figs. 5e and 5f). The stretching treatment plays a dual role: it promotes iodine uptake into the PVA matrix (Fig. S28) and induces crystallization to create a more rigid environment (Fig. 4d). Meanwhile, boric acid further suppresses the phosphorescent non-radiative rate constant (K_{nr}^{P}) by forming cross-linked networks with the PVA chains (Fig. 4a). Together, these synergistic effects account for the enhanced RTP performance.

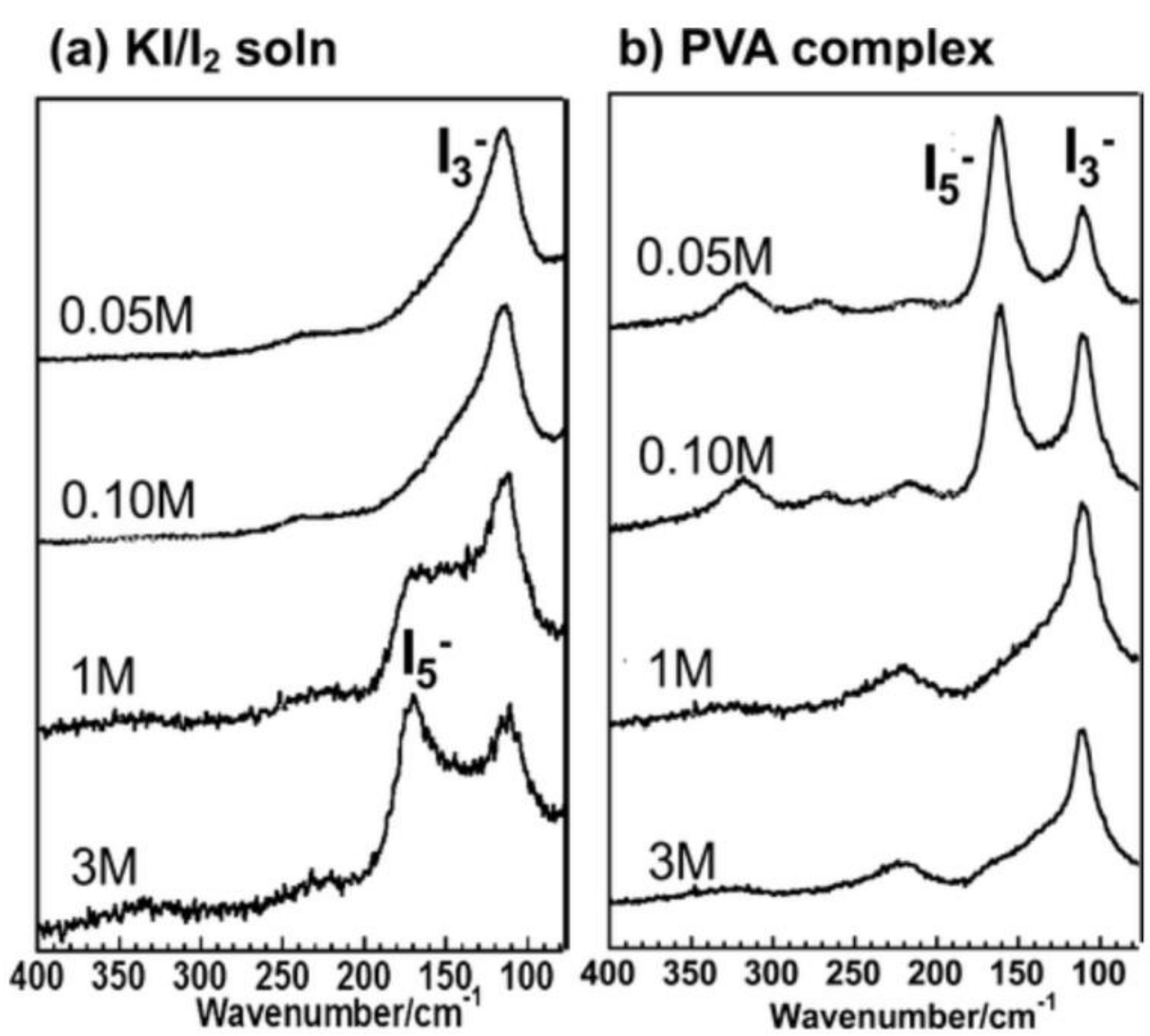

Fig. R2. Iodine concentration dependence of Raman spectra measured for (a) KI/I_2 aqueous solutions and (b) the PVA samples dipped in the KI/I_2 aqueous solutions with the various iodine concentrations. Cited from *Macromolecules* 2015, 48: 2138-2148.

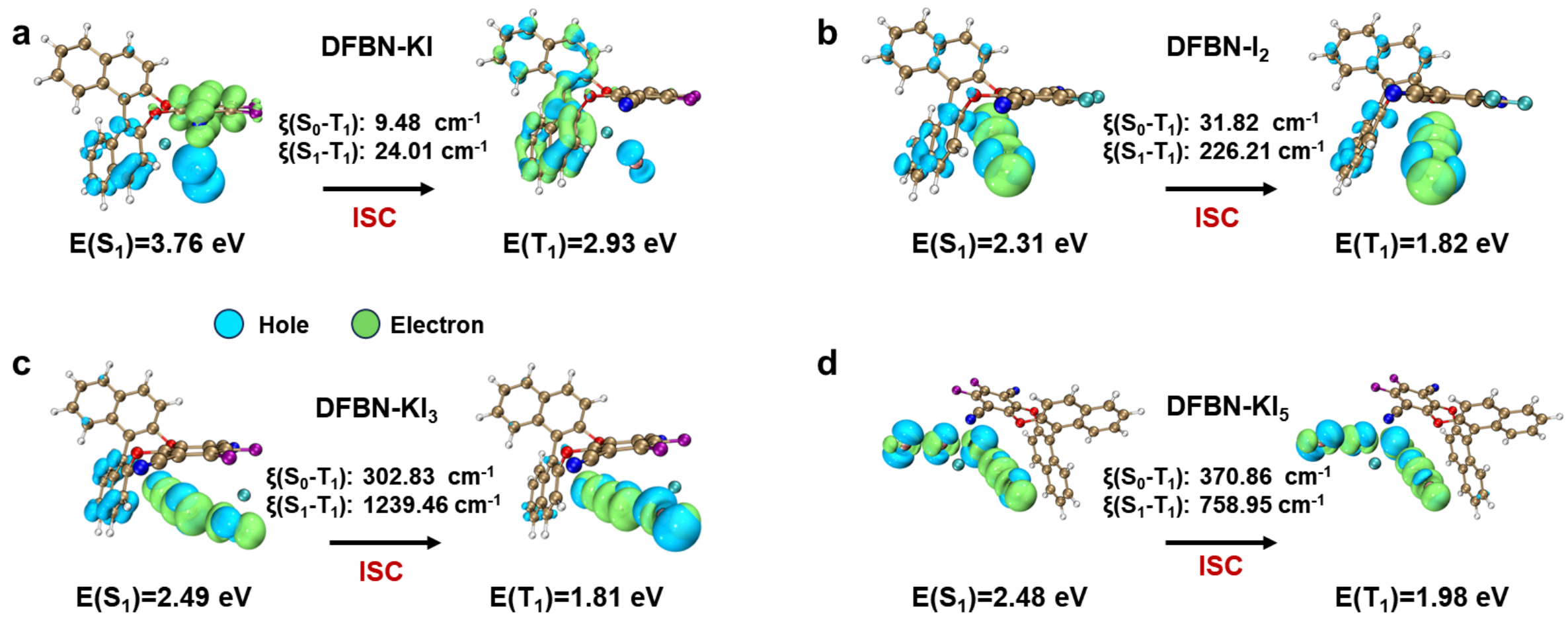

Fig. R3 (Fig. S36). Electron-hole analysis, SOC constants, and energy levels of the S_1 and T_1 states of (a) DFBN-KI, (b) DFBN-I_2, (c) DFBN-KI_3, and (d) DFBN-KI_5 models based on their S_0 optimized geometry.

3. Based on Question 2, the reviewer recommends that the authors provide a more detailed discussion in the manuscript to clarify and highlight the superiority of the synergistic ISB strategy.

Response: Thank you very much for your professional suggestion. Since this issue requires a consolidated summary of the revised manuscript, we have addressed it by integrating our responses to the other reviewers' comments. Initially, the temperature-dependent in-situ FT-IR spectra of DFBN@PVA were refined, and data analysis showed that both iodine-treated and boric-acid-treated DFBN@PVA films exhibited markedly enhanced thermal stability (Fig. S47), comparable to that achieved by the ISB-treated counterpart. In addition, we have reanalyzed the fs-TA data to clarify how the ISB strategy transforms the afterglow emission of DCBN from TADF into RTP and further enhances the RTP process. Detailed discussion can be found in the revised manuscript and in our response to Question #6, Reviewer 2. Quantitative analysis of iodine and boron contents in the BIN films reveals that a relatively high iodine concentration (N_I:$N_{Phosphors}$ = 1:10) is a key factor enabling the accelerated ISC process in the BIN systems. Relative description can be found to Question #7, Reviewer 2. Furthermore, a comparative study of BIN films subjected to different ISB treatments and thermal annealing at 120 °C under vacuum demonstrates that the ISB strategy achieves RTP enhancement comparable to that of thermal annealing, while offering clear advantages in terms of processing conditions (Fig. R16).

4. Fig. 4d indicates that the post-ISB samples exhibit higher crystallinity. However, the authors also state that "the incorporation of iodine disrupts the PVA crystalline order" in these samples. The authors are encouraged to discuss this apparent contradiction.

Response: Thank you for your valuable suggestion. As shown in Fig. 4d, powder XRD analysis reveals that the crystallinity of DFBN@PVA film initially increases (from $I_{10}S_2B_2$ to $I_{30}S_2B_2$) but

subsequently declines with prolonged ISB treatment ($I_{40}S_2B_2$), implying that moderate iodine incorporation facilitates crystallization, whereas excessive iodine is detrimental. Thus, we discussed in the revised manuscript that "Furthermore, the reduction in 2D SAXS diffraction rings and the position of the scattering peaks at q ~ 0.06 Å shifting to higher q values in 1D SAXS indicate a decline in both the long-range periodicity and crystal thickness of BINs@PVA films (Figs. 4f and S31b), likely due to the remolding of PVA crystalline order by the incorporation of iodine."

The incorporation of iodine ions bridges PVA microfibrils through PVA-iodine complexation, acting as junction points within the PVA network. Therefore, crystallization behavior of PVA is inevitably influenced for which the structural change from the lamellar structure to the microfibrillar structure is associated with the lamellar breakup upon the film drawing. These conclusions are in line with the literature reports (*Polymer* **2005**, 46, 7436-7442 and *J. Appl. Polym. Sci*. **1985**, 30, 1617).

In addition, temperature-dependent in-situ FT-IR spectra of DFBN@PVA films were refined (Fig. R4), in which iodine-treated DFBN films exhibit thermal stability comparable to that of the boron-treated counterparts, an outcome that is highly unexpected and further substantiates the role of iodine species as effective junctions within the PVA matrix. This finding also indirectly indicates the role of iodine in remodeling the crystalline order of PVA.

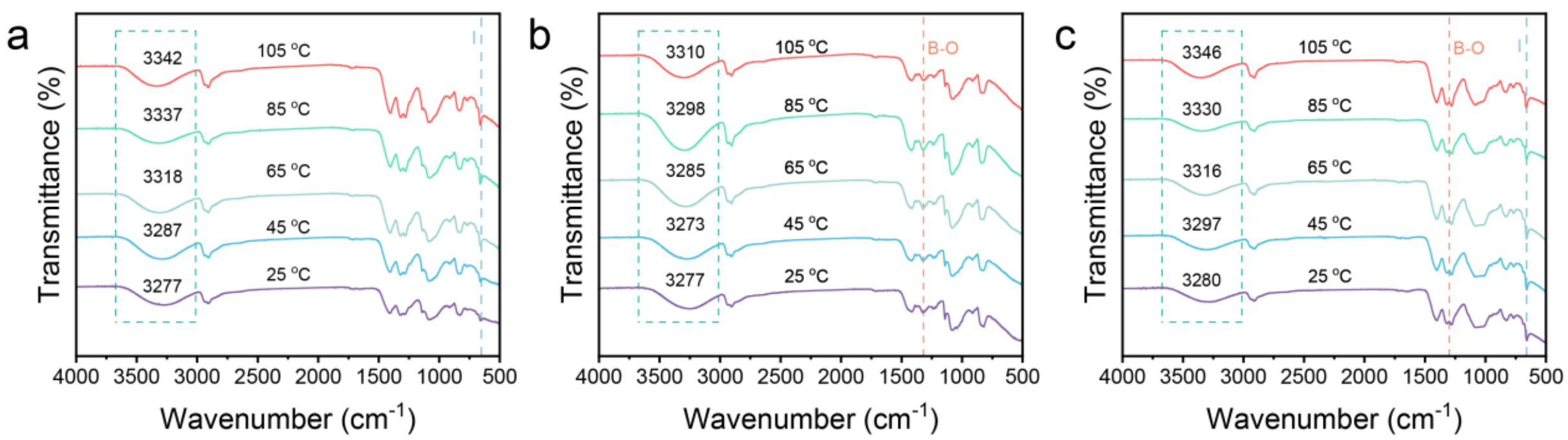

Fig. R4 (Fig. S47). Temperature-dependent in-situ FT-IR spectra of (a) iodine-treated DFBN@PVA film, (b) boron-treated DFBN@PVA film, and (c) ISB-treated DFBN@PVA film with the heating rate of 2 °C min^{-1}.

5. Figs. 4e and 4f show that the 2D scattering patterns of the post-ISB samples are circular, indicative of isotropic crystal planes and crystal orientations in the DFBN@PVA films. This observation seems contrary to the stretching-induced orientation of PVA chains illustrated in Fig. 1 and the SEM result in Fig. 4g. The authors should clarify this inconsistency.

Response: Many thanks for your constructive comments. The seemingly inconsistent results between the 2D WAXS/SAXS patterns (Figs. 4e and 4f) and the SEM images (Figs. 4g and S34) actually arise from the different structural scales probed by these instruments. The circular rings observed in the 2D WAXS and SAXS patterns indicate that the crystalline domains at the nanometer scale remain isotopically oriented after ISB treatment. This conclusion is further

confirmed by the azimuthally integrated 1D profiles (Fig. S31), which show enhanced crystallinity but no anisotropic features that would indicate crystal alignment. In contrast, the SEM images capture micrometer-scale morphological features of the PVA matrix. The fibrillar textures observed after ISB reflect macroscopic chain-segment alignment or surface stress-induced patterns, but they do not correspond to the orientation of crystalline lattices. Therefore, the SEM morphology and the isotropic WAXS/SAXS patterns represent different structural hierarchy levels and are not contradictory.

A similar phenomenon has also been reported previously by 2D SAXS and was attributed to the relatively low iodine content in the polymer film (Fig. R5).

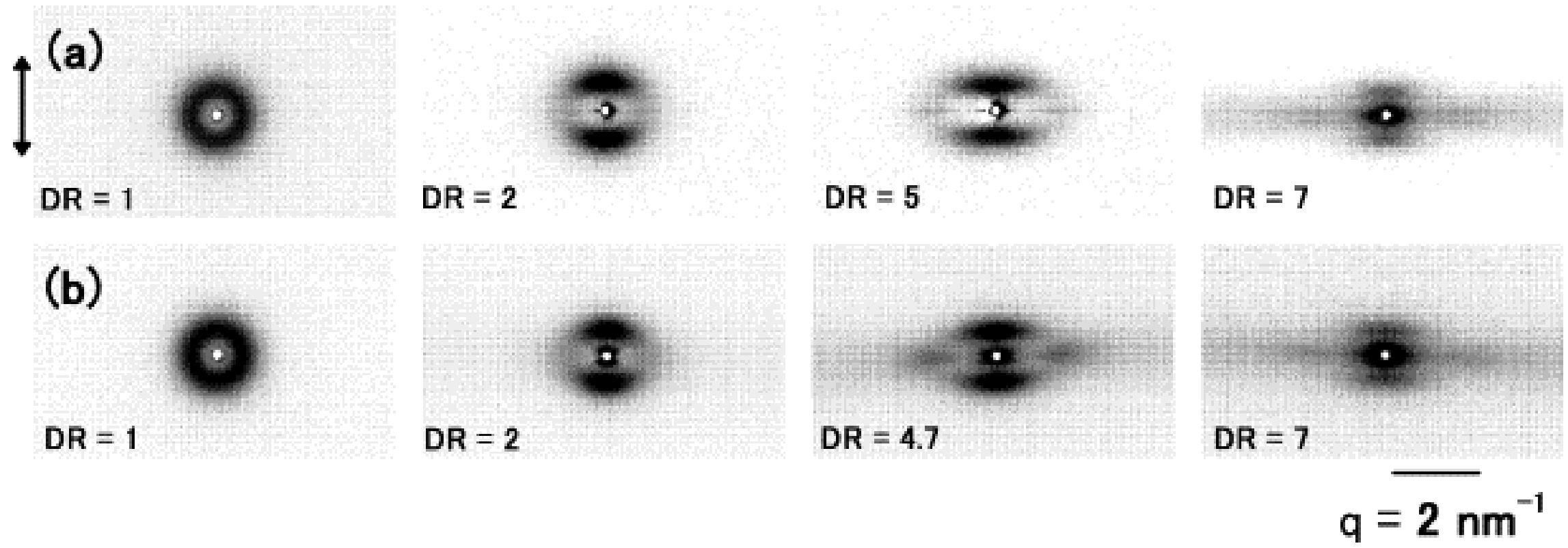

Fig. R5. Two-dimensional small-angle X-ray scattering patterns for the films drawn in pure water (a) and in a KI/I_2 solution (b) with the draw ratio (DR) indicated in the figures. The arrow indicates the draw direction. Cited from *Polymer* **2005**, 46, 7436-7442.

6. If high crystallinity indeed facilitates the formation of I_3^- and I_5^- coordination structures, thereby enhancing both τ_p and Φ_p, it is suggested that the schematic in Fig. 1 incorporate the roles of PVA crystallinity and polyiodide coordination. However, the reviewer leaves it to the authors' discretion to decide whether this addition is necessary.

Response: We thank the valuable advice. In this work, the stretching process plays a significantly dominant role in promoting the formation of coordinated I_3^- and I_5^- species, as evidenced by the Raman spectra (Figs. 4b and S28). Notably, the enhanced crystallization originates from the incorporation of iodine, rather than crystallization facilitating iodine uptake. In addition, powder XRD spectra indicate that the films are mainly amorphous (Fig. 4d). Nevertheless, we fully respect reviewer's suggestion, and accordingly have added a textual description in Fig. R6 (Fig. 1) to clarify the RTP performance changes induced by the ISB treatment.

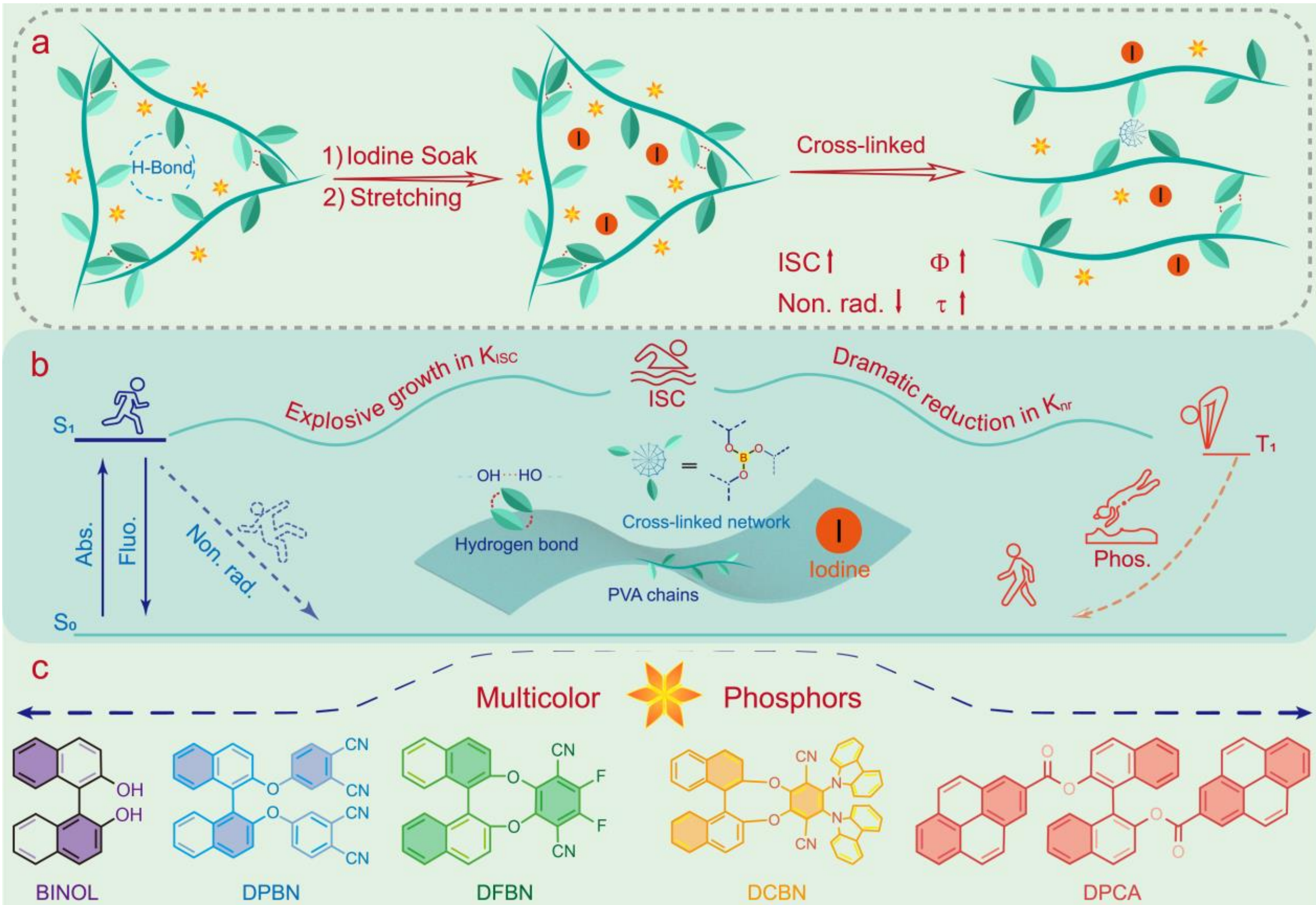

Fig. R6 (Fig. 1). **Schematic illustration of ISB enhancing RTP. (a)** Strategy of ISB treatment for enhancing RTP performance. **(b)** Jablonski illustration of efficient and long-lived RTP emission via ISB and schematic diagram of hydrogen bond, iodine, crosslink network and PVA chains. Abs. stands for absorption, Fluo. for fluorescence, Non. rad. for non-radiative, and Phos. for phosphorescence. **(c)** Chemical structure of BINOL, DPBN, DFBN, DCBN and DPCA molecules.

Reviewer #2 (Remarks to the Author):

In this work, the authors prepared a series of PVA-based afterglow materials using an Iodine-Stretch-Boric Acid (ISB) strategy and systematically characterized their fundamental properties and photophysical performance. They claim that the ISB strategy not only enhances the rigidity of PVA but also significantly boosts phosphorescence through the external heavy-atom effect. While it is well established that both matrix rigidification and the external heavy-atom effect can enhance phosphorescence, strategies that synergistically combine these two effects to achieve substantial performance improvements are relatively rare. Therefore, I acknowledge that this work possesses a certain degree of novelty. However, regrettably, the data presented do not adequately support the authors' conclusions. In particular, the reported phosphorescence quantum yields are provided only as numerical values without supporting evidence or detailed methodology, severely undermining their credibility. The same issue applies to the non-radiative decay rates. Considering the concerns outlined below, I believe the overall quality of the manuscript does not meet the standards expected for Nature Communications. Therefore, I recommend rejection.

Response: We appreciate the valuable comments from the reviewer on our work. Developing polymeric materials for efficient phosphorescence generation is a highly promising field in the lighting/imaging industries. However, the advancement of polymeric materials with exceptional room temperature phosphorescence (RTP) performance is hindered by the competing relationship between long phosphorescence lifetime (τ_P) and high phosphorescence quantum yield (Φ_P). Introducing heavy atom effects (HAE) into RTP systems, such as incorporating halogen bonds into phosphors or embedding them into a polymer matrix through molecular design, often facilitates the intersystem crossing (ISC) of triplet excitons, leading to efficient RTP emission. Nevertheless, this method often comes with sacrificing phosphorescence lifetime, along with simultaneous acceleration of both ISC rate constant (K_{ISC}) and phosphorescence rate constant (KP). In this work, we present a strategy via Iodine-Stretch-Boric Acid (ISB) treatment that synergistically fine-tunes the generation and quenching of triplet excitons, thereby improving both τ_P and Φ_P. In this strategy, ISB harnesses the external HAE to facilitate spin-orbit coupling of phosphors, while simultaneously forming an ultra-dense cross-linked network in polymeric matrix. This dual modulation leads to a significant optimization of the non-radiative rate constant (K_{nr}^{P}) and K_{ISC} of phosphorescence emission. Notably, the RTP performance of post-ISB treated films attain a record-high advancement. Furthermore, ISB treatment not only provides a general strategy for designing high-performance RTP materials, but also improves the durability of the RTP films. The ISB treatment can preserve bright RTP emission after five days of water immersion and 12 hours in acidic, alkaline and high temperature conditions, exhibiting substantial potential for multicolor secure information, antibacterial, and water resistant RTP applications.

Therefore, this work presents an efficient strategy for developing polymeric RTP materials with long τ_P, high Φ_P and excellent durability. We sincerely hope that our revisions, along with optimized details, can clear the issues raised by the reviewer.

1. Steady-state emission spectra should be provided. These spectra are essential for understanding the luminescent behavior of the system, particularly the relative contributions of fluorescence and phosphorescence.

Response: We apologize for any confusion that may have led you to believe that the steady-state emission spectra were not provided. In fact, the relevant spectral data are presented in Fig. S18. Nevertheless, we fully respect the reviewer's suggestion and have revised the y-axis label and the figure caption to make the information clearer for both the reviewers and readers.

2. To the best of our knowledge, absolute photoluminescence quantum yield instruments measure steady-state emission and lack time-gating capability. Thus, accurate determination of phosphorescence quantum yield remains a significant challenge. Since phosphorescence quantum yield is a key metric for evaluating the effectiveness of the ISB strategy, the authors must disclose the exact methodology used. Given the severe spectral overlap between fluorescence and phosphorescence in this system (Fig. S13), the authors should record full decay curves and calculate fluorescence and phosphorescence quantum yields based on the integrated areas of the fast (fluorescent) and slow (phosphorescent) decay components.

Response: We sincerely appreciate your valuable comments to use absolute quantum yield measurements with gated detection for BIN films on our work. We apologize for the lack of methodological details in the original manuscript. We have now added the corresponding description in the revised version and emphasized that the quantum yield reported is the absolute quantum yield in the revised manuscript.

To address the reviewer's concern, we have provided the corresponding raw data for the absolute quantum yield measurements in Figs. R7 and R8, which represent the absolute photoluminescence quantum yield (Φ_{PL}) and phosphorescence quantum yield (Φ_P) of DPBN@PVA, DFBN@PVA, DCBN@PVA, and DPCA@PVA films before and after ISB treatment. The Φ_{PL} and Φ_P were measured by FLS1000 spectrometer equipped with an integrating sphere and time-gated detection. The samples were excited at a designated wavelength, and the total emitted photons were collected over all solid angles. The quantum yield was calculated by comparing the integrated emission intensity with the number of absorbed excitation photons.

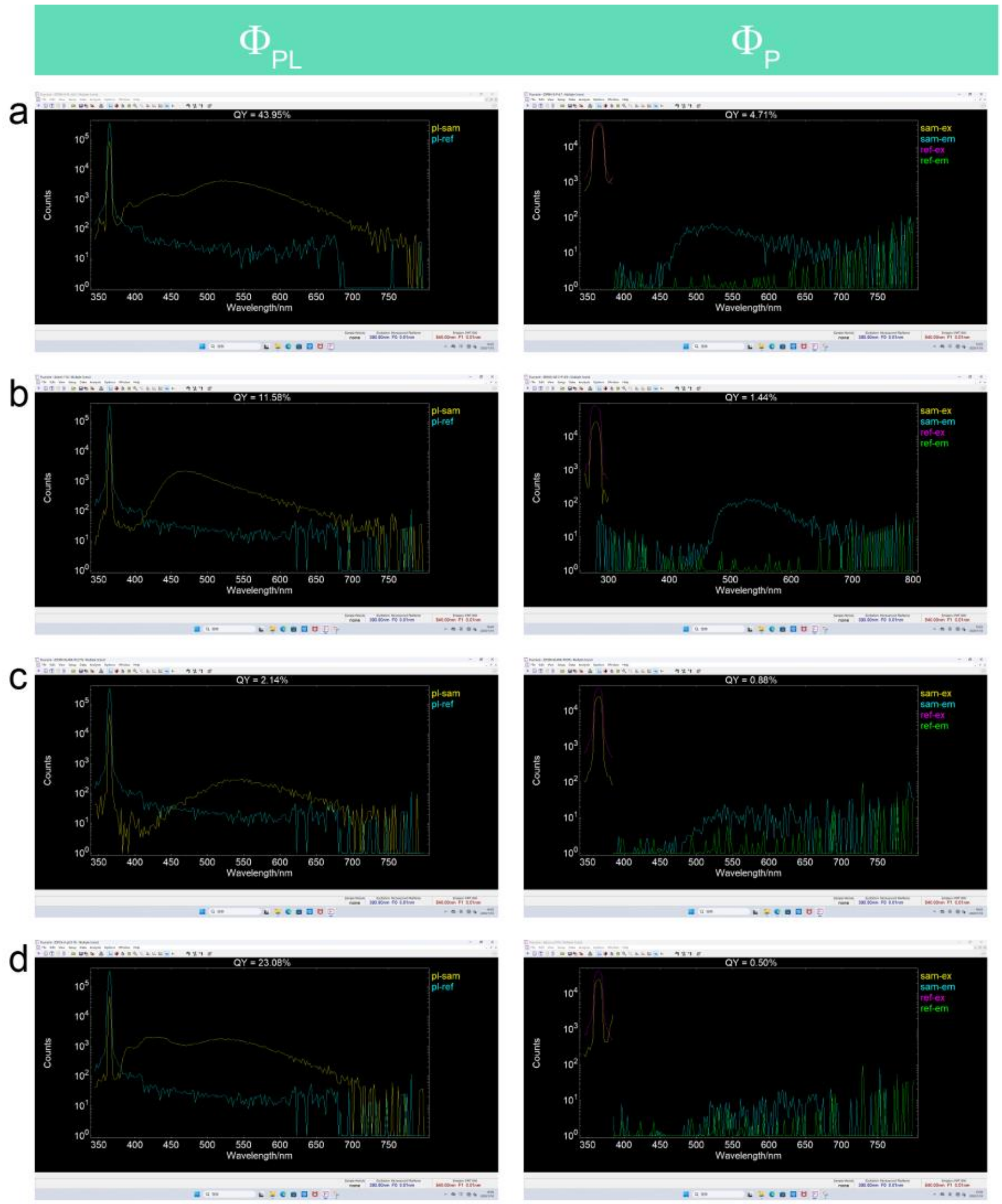

Fig. R7 (Fig. S21). Raw data for Φ_{PL} and Φ_P of (a) DPBN@PVA, (b) DFBN@PVA, (c) DCBN@PVA, and (d) DPCA@PVA films before ISB treatment.

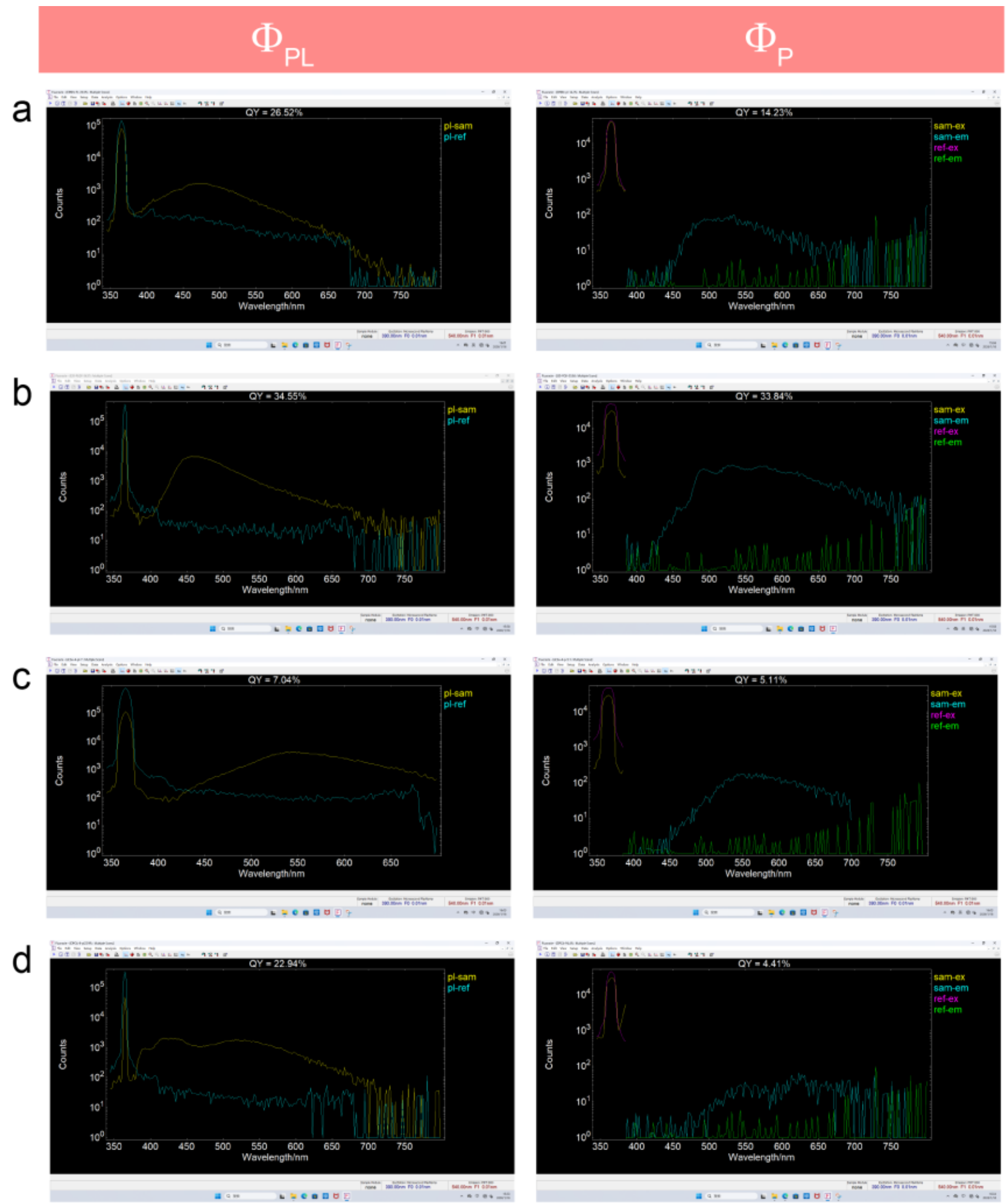

Fig. R8 (Fig. S22). Raw data for Φ_{PL} and Φ_P of (a) DPBN@PVA, (b) DFBN@PVA, (c) DCBN@PVA, and (d) DPCA@PVA films after ISB treatment.

3. In Table S1, why does the fluorescence lifetime increase after ISB treatment? Take DPBN as an example: its fluorescence quantum yield drops sharply from 39.3% to 12.3%, while its phosphorescence quantum yield rises from 4.7% to 14.2%. This strongly suggests that the heavy atom effect significantly enhances ISC from S_1 to T_1. Under such circumstances, the fluorescence lifetime should decrease due to accelerated S_1 decay.

Response: Many thanks for the question. Your careful and detailed review has been invaluable in enhancing the quality of our work. First, we have remeasured the fluorescence lifetime (τ_F) of the BINs@PVA films to exclude any potential experimental or data-processing errors (Fig. R9). The results demonstrate that the relative variation trends of the τ_F for BINs@PVA films before and after ISB treatment remain consistent with those obtained in the initial measurements, thereby ruling out the possibility of measurement artifacts.

Second, it is well established that a rigid environment predominantly suppresses non-radiative decay, leading to prolonged excited-state lifetimes, whereas the most direct

consequence of the heavy-atom effect is the enhancing SOC, which facilitates ISC and significantly modulates the QY. However, this does not imply that the heavy-atom effect has no influence on lifetime, nor that a rigid environment is irrelevant to quantum yield. As stated in our manuscript, “Generally, the quantum yield and lifetime of phosphorescence are inversely correlated due to the trade-off between competing radiative and non-radiative decay processes.” Analogously, an inverse relationship between fluorescence quantum yield and fluorescence lifetime is also theoretically reasonable.

Third, according to the kinetic expressions (1), the fluorescence lifetime is jointly governed by the radiative decay rate, non-radiative decay rate, and ISC rate. Although the heavy-atom effect introduced by ISB can markedly enhance K_{ISC}, ISB represents a composite modulation strategy. The concomitant changes in crystallinity, molecular orientation, heavy-atom incorporation, and cross-linking may simultaneously and competitively influence these rate constants. Consequently, when the reduction in radiative and non-radiative decay rates outweighs the increase in ISC rate, a negative correlation between τ_F and QY becomes theoretically plausible.

$$\tau = \frac{1}{K_r^F + K_{nr}^F + K_{ISC}} \qquad (1)$$

Meanwhile, we found that similar opposite trends have been reported in literature, indicating that this observation in Table S1 is not an isolated case (Fig. R10).

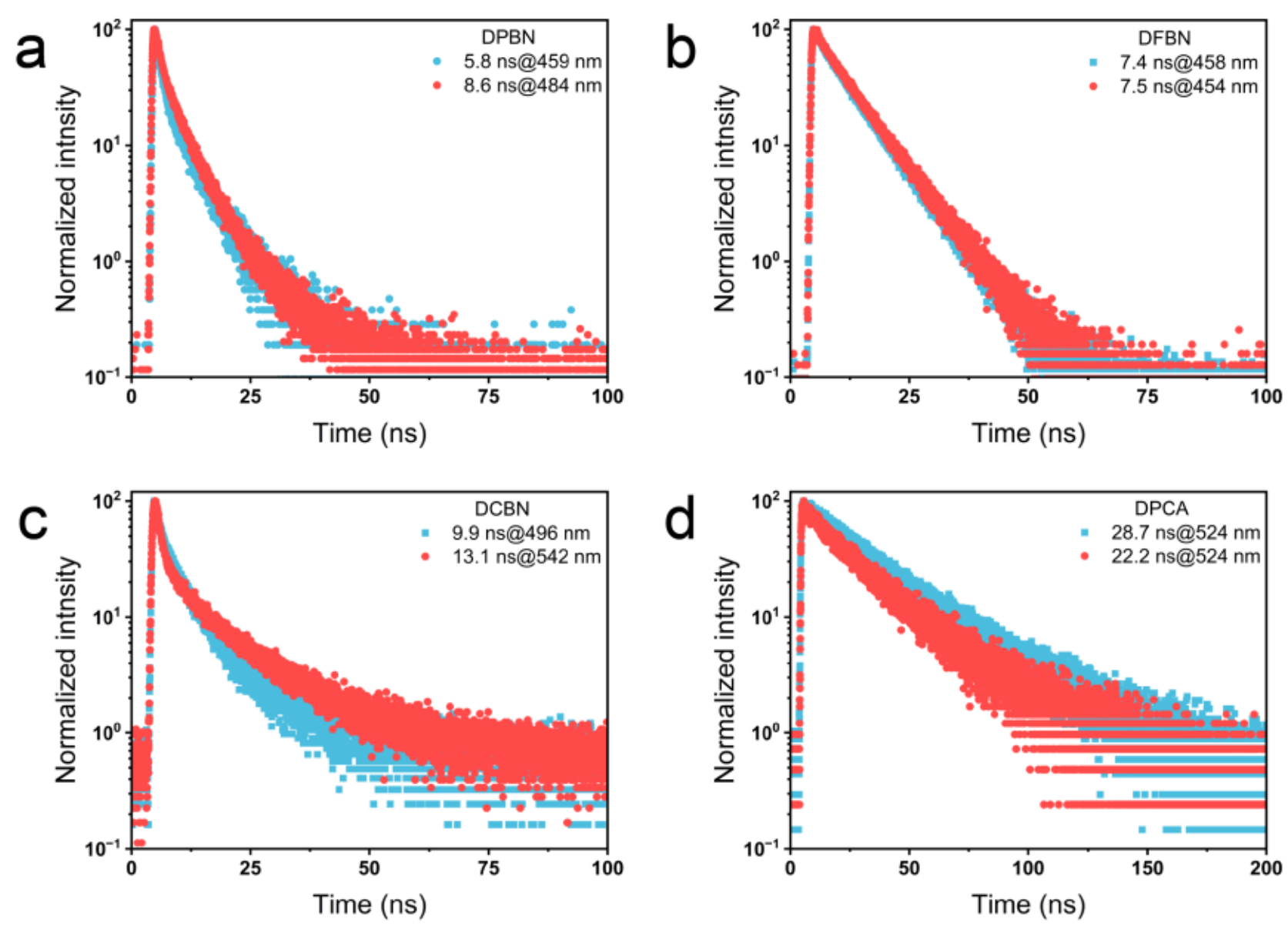

Fig. R9. Remeasured fluorescence lifetime profiles of (a) DPBN@PVA, (b) DFBN@PVA, (c) DCBN@PVA, and (d) DPCA@PVA films.

a

Isomers	$\lambda_{em,F}$ [nm]	τ_F [ns]	Φ_F [%]	k_r^F [s^{-1}][a]	k_{nr}^F [s^{-1}][b]	k_{ISC} [s^{-1}][c]	$\lambda_{em,P}$ [nm]	τ_P [ms]	Φ_P [%]	k_r^P [s^{-1}][d]	k_{nr}^P [s^{-1}][e]
**26CIM**	451	5.43	9.70	1.78×10^7	7.62×10^7	9.00×10^7	486	32.42	48.90	1.50×10^4	1.58×10^4
**246CIM**	450	7.43	5.50	7.40×10^6	1.04×10^8	2.30×10^7	504	65.52	17.10	2.61×10^3	1.27×10^4
**25CIM**	453	1.91	3.30	1.72×10^7	4.76×10^8	2.93×10^7	528	129.62	5.60	4.32×10^2	7.28×10^3
**24CIM**	450	6.23	3.40	5.45×10^6	1.50×10^8	1.01×10^7	530	21.0	2.90	1.38×10^3	4.62×10^4

[a] $k_r^F = \Phi_F/\tau_F$; [b] $k_{nr}^F = (1-\Phi_F-\Phi_P)/\tau_F$; [c] $k_{ISC} = \Phi_P/\tau_F$; [d] $k_r^P = \Phi_P/\tau_P$; [e] $k_{nr}^P = (1-\Phi_P)/\tau_P$

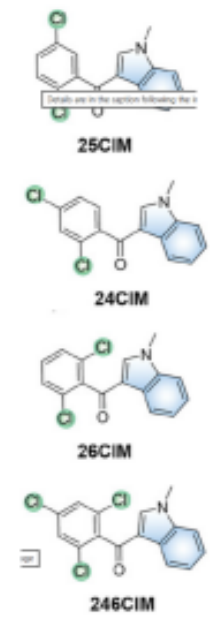

b

Compound	Wavelength (nm)	Fluorescence		Phosphorescence			
		ϕ_F (%)	τ_F (ns)	ϕ_P (%)	τ_P (ms)	%	$\langle\tau\rangle_P$ (ms)
T3-*m*-PyCzBr	370-450[a)] 540/589/640	2.0	0.45	9	108/524	58/49	319
T2-*p*-PyCzBr-C1	370-450[a)] 540/589/640	1.5	0.77	20	97/432	69/31	200

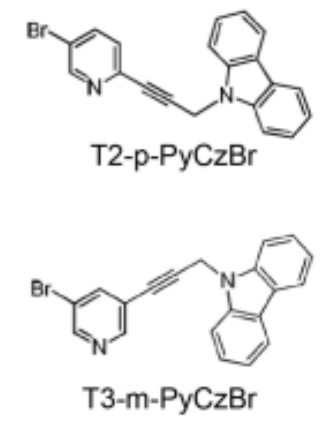

c

Compounds	$\lambda_{em,F}$ (nm)	τ_F (ns)	ϕ_F (%)[a]	$\lambda_{em,P}$ (nm)	τ_P (ms)	ϕ_P (%)[a]
DOPTZ-C3	336/352	1.26	2.3	443/518	31/58	11.4
DOPTZ-C2Cl	332/370	0.80	0.9	446/521	345/244	3.0
DOPTZ-C3Cl	339	0.78	1.4	504/525	1257/1351	10.1
DOPTZ-C4Cl	372	0.84	1.7	446/508	264/664	13.7
DOPTZ-C3Br	346	1.59	1.7	446/510	146/974	16.6
DOPTZ-C3I	340	0.57	0.2	463	74	0.8

[a] The absolute quantum yield was measured in solid state at room temperature.

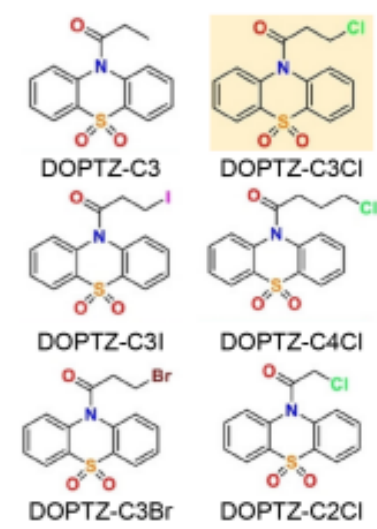

Fig. R10. Reverse trends between τ_F and Φ_F in reported literature, *Adv. Opt. Mater*. 2023, **11**, 2201641; *Angew. Chem. Int. Ed*. 2021, **60**, 2446-2454; *Angew. Chem. Int. Ed*. 2025, **64**, e202419366.

4. How were the rate constants K_{ISC}, $K_{P\,nr}$, K_{nr}^F calculated? Were they derived from Φ/τ? If so, please provide the calculation procedures and raw data for key parameters such as Φ_{ISC}, Φ_{nr}^F, $\Phi_{P\,nr}$. Moreover, we recommend using transient absorption spectroscopy to directly determine K_{nr}^F and K_{ISC}; otherwise, the analysis risks circular reasoning.

Response: Many thanks for the constructive comment. As you anticipated, these data were indeed obtained from kinetic calculations. During our initial fs-TA investigations aimed at elucidating the evolution of afterglow emission in DCBN from TADF to RTP, fs-TA measurements were also performed on the other three BINs films. Owing to the poor optical transparency of the DCBN films, reliable excited-state signals associated with DPCA could not be fully resolved. In addition, the fluorescence emission of DFBN films is centered at approximately 455 nm (Figure S18), which has a spectral overlap with the DFBN films having ESA band located around 450 nm. As a result, the SE and ESA signals cannot be clearly disentangled in 3D fs-TA spectra, thereby complicating the reliable extraction of photophysical rate constants such as K_{ISC} and K_{nr}^F from the fs-TA data. Simultaneously, in Figure R12b, the SE signal also substantially interferes with the ESA features. In addition, it should be reasonable to calculate these constants via kinetic calculations

in RTP fields. Therefore, K_{nr}^{F} and K_{ISC} might not be calculated via TA spectra. Herein, kinetic calculations might represent a more suitable choice. The calculation procedures have been added to the Methods section of the manuscript under "Kinetic calculations and photophysical rate constants". The source of raw data has been indicated below Table R1 (Table S1).

Table R1 (Table S1). Kinetic calculations and photophysical rate constants of pre-ISB and post-ISB treated BINs@PVA films.

Sample		Prompt photoluminescence					Delayed photoluminescence			
		τ_F ns	Φ_F (%)	K_r^F (s^{-1})	K_{nr}^F (s^{-1})	K_{ISC} (s^{-1})	τ_P (ms)	Φ_P (%)	K_r^P (s^{-1})	K_{nr}^P (s^{-1})
DPBN@PVA	Pre-ISB	4.3	39.3	9.14×10^{7}	1.30×10^{8}	1.09×10^{7}	944.4	4.7	4.98×10^{-2}	1.01
	Post-ISB	5.2	12.3	2.37×10^{7}	1.41×10^{8}	2.73×10^{7}	1690.3	14.2	8.40×10^{-2}	0.51
DFBN@PVA	Pre-ISB	7.6	10.2	1.34×10^{7}	1.16×10^{8}	1.84×10^{6}	43.7	1.4	3.20×10^{-1}	22.56
	Post-ISB	8.0	0.7	8.75×10^{5}	8.19×10^{7}	4.23×10^{7}	1228.4	33.8	2.75×10^{-1}	0.54
DCBN@PVA	Pre-ISB	3.8	1.2	3.16×10^{6}	2.58×10^{8}	2.37×10^{6}	47	0.9	1.91×10^{-1}	21.09
	Post-ISB	10.4	1.9	1.83×10^{6}	8.94×10^{7}	4.90×10^{6}	293.6	5.1	1.74×10^{-1}	3.23
DPCA@PVA	Pre-ISB	27.2	22.6	8.31×10^{6}	2.83×10^{7}	1.84×10^{5}	30	0.5	1.67×10^{-1}	33.17
	Post-ISB	29.7	18.5	6.23×10^{6}	2.6×10^{7}	1.48×10^{6}	205.6	4.4	2.14×10^{-1}	4.65

Note: τ_F and τ_P originate from Fig. S18. and Fig. 2f, and absolute Φ_F and Φ_P originate from Fig. S21.

5. Why were 77 K photophysical measurements performed in DCM? Standard practice uses glass-forming solvents such as 2-methyltetrahydrofuran.

Response: Thank you for your meticulous review and the valuable guidance. We have re-investigated the photophysical behavior of BIN molecules at 77 K using 2-methyltetrahydrofuran as the solvent, and the corresponding experimental data are summarized in Fig. R11.

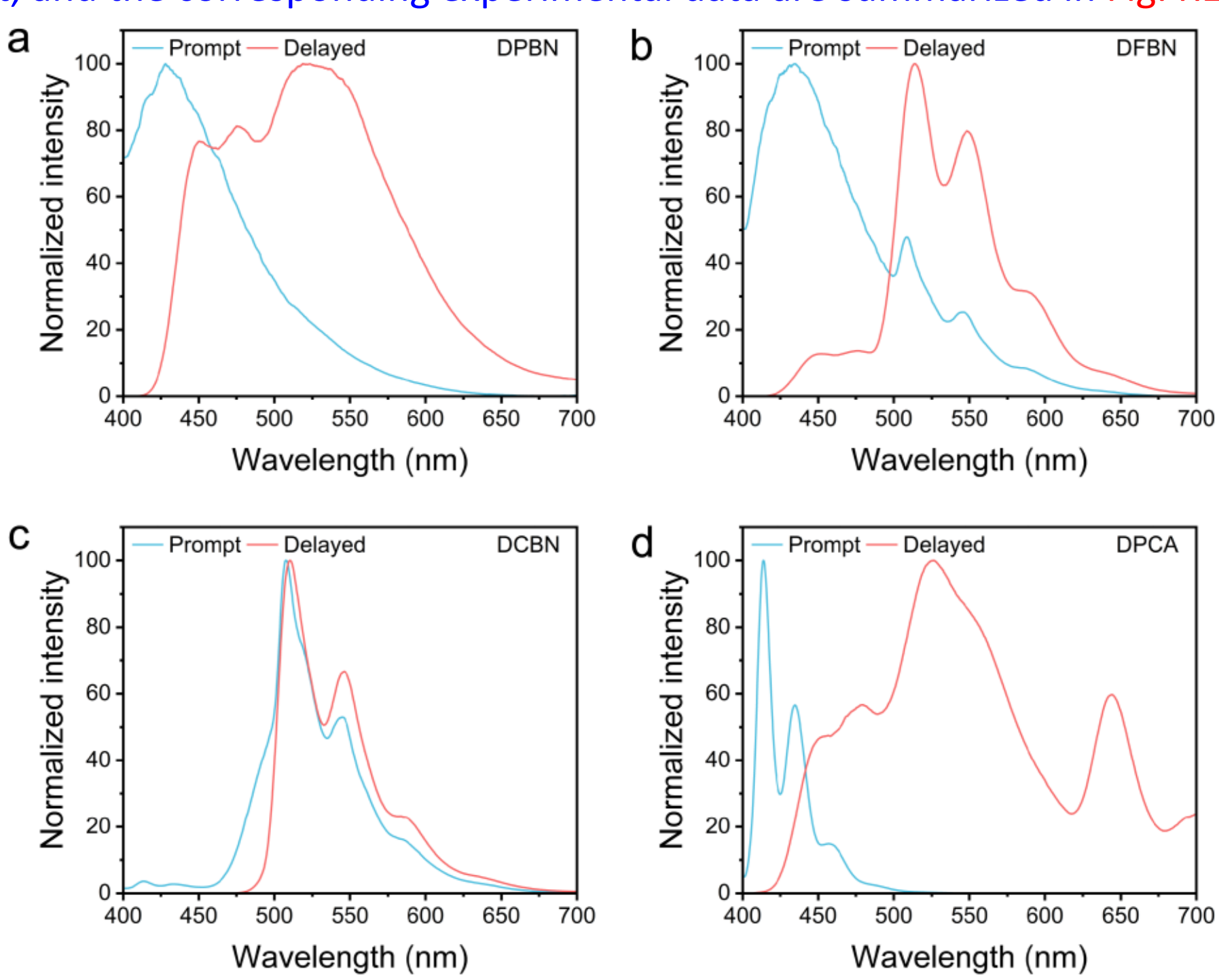

Fig. R11 (Fig. 3f and S13). Prompt and delayed spectra of (a) DPBN, (b) DFBN, (c) DCBN, and (d) DPCA in 2-methyltetrahydrofuran (10^{-5} mol/L) at 77 K. The measurements were carried out using an F-4700 fluorescence spectrophotometer.

6. The TA data (Fig. S24) fail to explain why BINs show no afterglow at 77 K but exhibit dramatically enhanced afterglow after BA treatment. The authors should focus on tracking S_1 decay dynamics and triplet formation before and after ISB treatment. Additionally, the TA data presentation is extremely poor. Fig. S24 is nearly unreadable. Notably, Fig. S24e reports a fitted lifetime of 127.2 ns, which is clearly unreasonable given the instrument's time window of only 7 ns.

Response: Many thanks for your question. We apologize for the inadequate treatment of the fs-TA data in the original manuscript. Following your suggestion, the fs-TA measurements were repeated and the corresponding analyses were performed carefully in the revised manuscript (Fig. R12) and Supporting Information (Fig. R13-R14). Related modifications in the manuscript are also shown below.

"Within the 1 ps to 5 ns time window, the femtosecond transient absorption (fs-TA) spectra of DCBN reveal two quasi-simultaneously generated excited-state absorption (ESA) bands centered at approximately 450 and 757 nm (Fig. 3i). The nearly synchronous temporal evolution of these two ESA features is characteristic of TADF, consistent with their assignment to a rapid reverse intersystem crossing (RISC) process. By contrast, the pristine DCBN@PVA film exhibits a gradual attenuation of the stimulated emission (SE) signal near 460 nm, accompanied by the emergence of a weak ESA feature (Fig. 3j), in agreement with its relatively inefficient RTP (Fig. 2h). The presence of well-defined isosbestic points in the fs-TA spectra of both pre-ISB and post-ISB treated films substantiates a clean transition between the lowest singlet and triplet excited-state manifolds. Upon ISB treatment, a pronounced ESA band rapidly develops at around 450 nm in the DCBN@PVA film (Fig. 3k). Compared with the untreated sample, this significant enhancement can reasonably be attributed to iodine-induced HAE, which effectively accelerates ISC process."

"Notably, the ESA signals at around 450 and 761 nm display opposite temporal evolution trends, associating with the characteristic triplet state population dynamics in phosphorescence. From a kinetic standpoint, the 450 nm feature is attributable to the lowest triplet excited state, whereas the 757 nm band originates from the singlet excited state. The pronounced shift in the SE signal of DCBN, transitioning from the isolated state to Pre-ISB and Post-ISB films, is ascribed to solid-state solvation effects, with the isosbestic points indicating that dielectric-controlled dynamics predominantly govern this process. Time-resolved absorption spectra provide additional support for this assignment (Figs. S25a-c). Quantitative analysis of the singlet state dynamics was performed by fitting the fs-TA kinetic traces extracted from the ESA regions using a biexponential decay model (Figs. S25d-f). Compared with the pre-ISB film, the post-ISB DCBN@PVA film exhibits a distinctly shortened singlet excited state lifetime concomitant with a prolonged triplet-state

persistence. This kinetic reconfiguration underscores the role of ISB as an efficient ISC accelerator, thereby favoring RTP emission."

"Further insight is gained spanning 1 fs to 1 ps. Isolated DCBN displays an ultrafast RISC process within this time window (Fig. S26a), whereas film-state DCBN is characterized by a pronounced SE signal. Given the close spectral proximity between the SE band and the lowest triplet ESA feature (Fig. S26b), this behavior indicates that RISC is substantially suppressed in the film state, with the weak triplet ESA signal being masked by SE. In post-ISB DCBN@PVA film, a distinct triplet ESA feature emerges within 1 ps. As evidenced by Fig. S18e, ISB treatment effectively modulates the ΔE_{ST} of DCBN. When combined with temperature-dependent delayed spectra (Fig. S26c), these observations unequivocally demonstrate that the afterglow emission of post-ISB DCBN@PVA originates from RTP rather than TADF. Consequently, the rapid emergence of the triplet ESA signal can be unambiguously attributed to ISB-promoted ISC, rather than RISC."

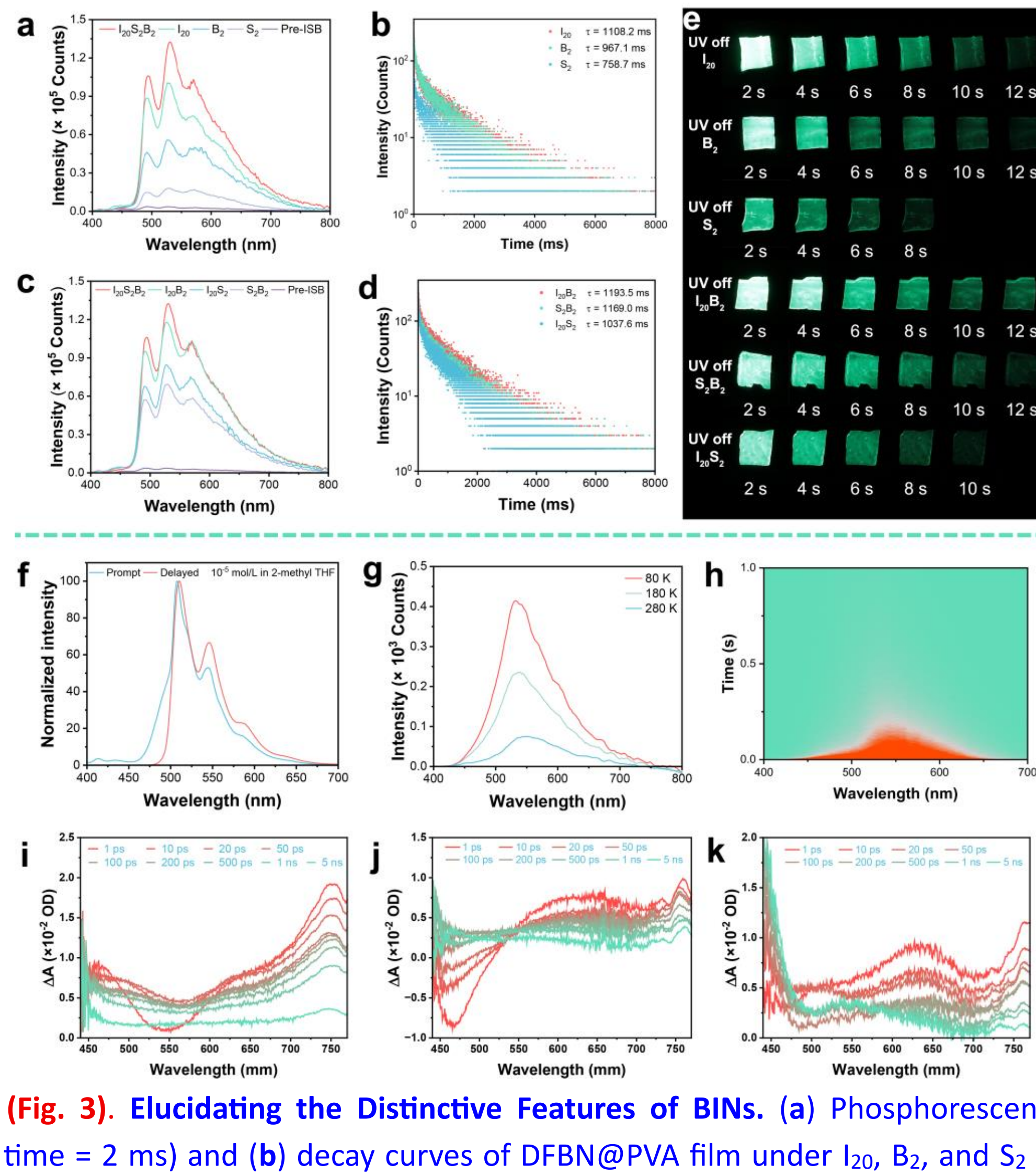

Fig. R12 (Fig. 3). **Elucidating the Distinctive Features of BINs.** (**a**) Phosphorescence spectra (delayed time = 2 ms) and (**b**) decay curves of DFBN@PVA film under I_{20}, B_2, and S_2 treatment, respectively. (**c**) Phosphorescence spectra (delayed time = 2 ms) and (**d**) decay curves of

DFBN@PVA film under $I_{20}B_2$, $I_{20}S_2$, and S_2B_2 treatment, respectively. (**e**) Afterglow photographs of DFBN@PVA films under various treatments. (**f**) Prompt spectra and delayed spectra of DCBN in 2-methyltetrahydrofuran (10 μM) at 77 K. (**g**) Temperature-dependent delayed spectra of DCBN@PVA film. (**h**) TRES of DCBN@PVA film. Transient absorption spectra of (**i**) isolated DCBN dispersed in DMSO, (**j**) pre-ISB DCBN@PVA film and (**k**) post-ISB DCBN@PVA film ranging from 1 ps to 5 ns.

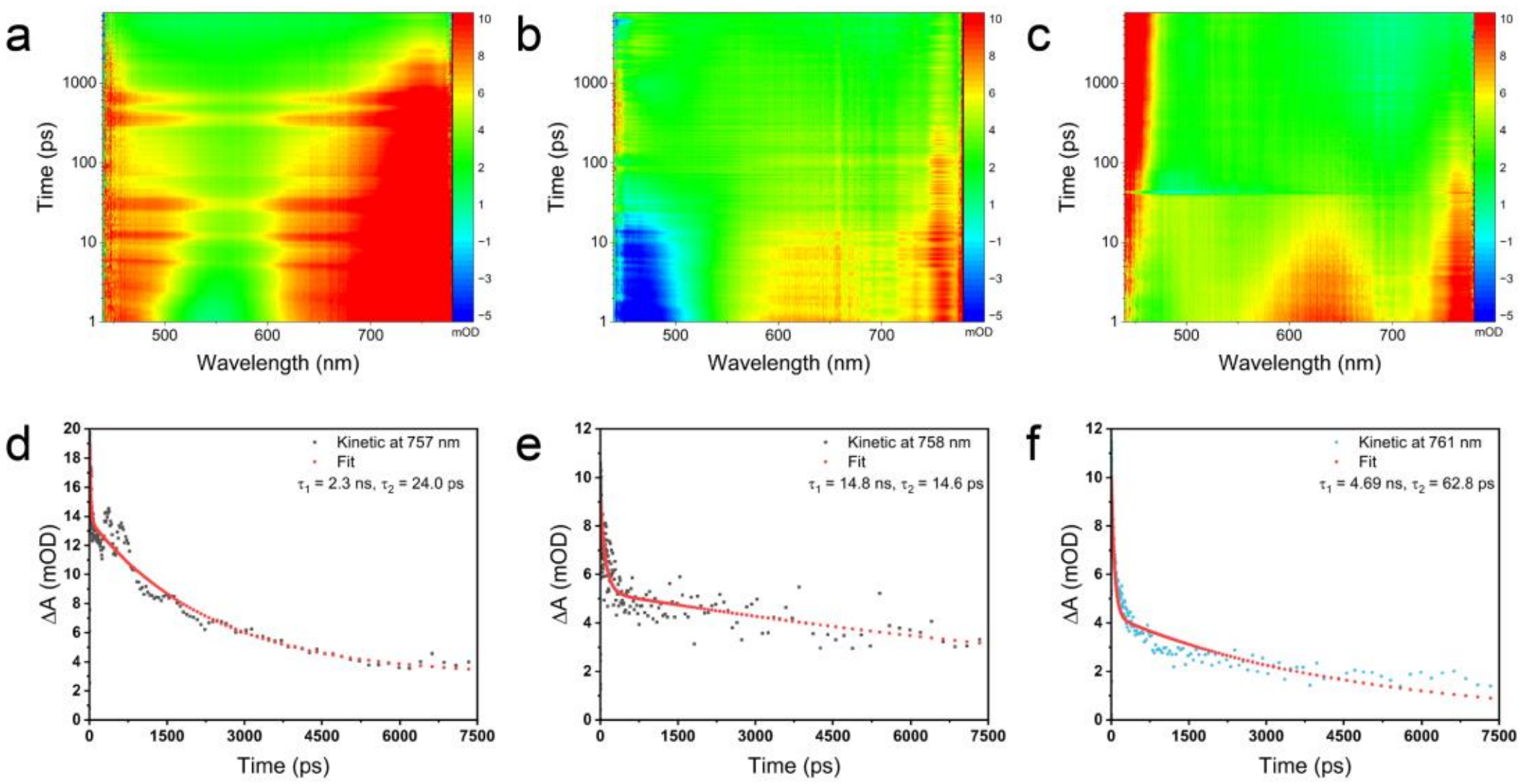

Fig. R13 (Fig. S25). Time-resolved absorption spectra of (a) isolated DCBN in DMSO, (b) pre-ISB DCBN@PVA film, and (c) post-ISB DCBN@PVA film. Kinetic curve of excited state absorption for (d) isolated DCBN in DMSO, (e) pre-ISB DCBN@PVA film, and (f) post-ISB DCBN@PVA film.

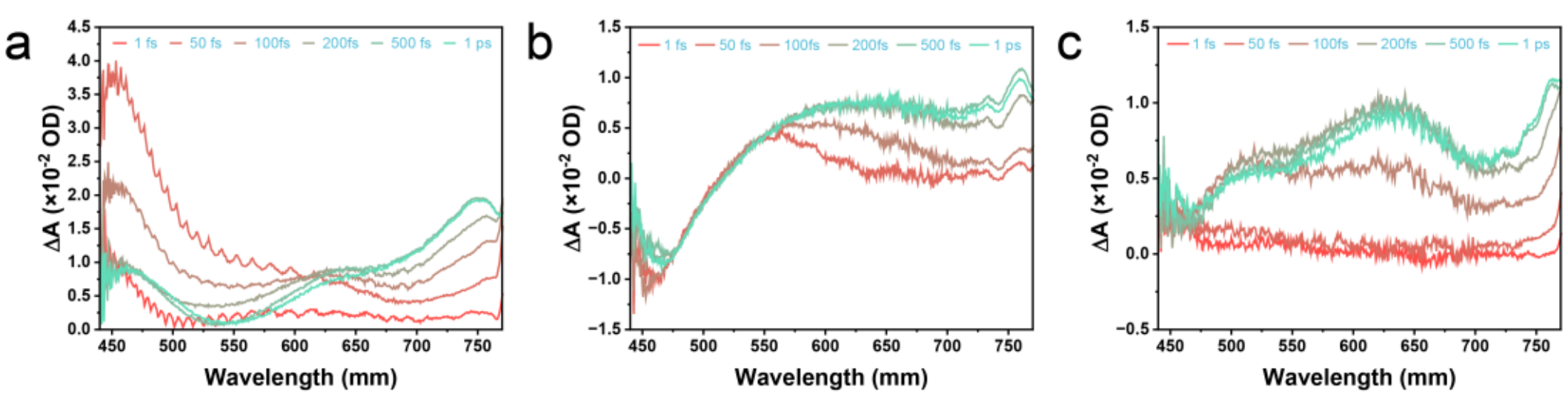

Fig. R14 (Fig. S26). Transient absorption spectra of (e) isolated DCBN dispersed in DMSO, (f) pre-ISB DCBN@PVA film and (g) post-ISB DCBN@PVA film ranging from 1 fs to 1 ps.

7. What are the actual iodine and boron contents in the films after ISB treatment? Elemental analysis would help clarify this.

Response: Tanks for your careful and detailed review. As shown in Table R2-R4, we have performed the inductively coupled plasma-mass spectrometry (ICP-MS) test to quantitatively determine the contents of I and B in the BINs films. Corresponding data analyses shown bellow have been added in revised manuscript and Supporting Information.

“As shown in Table S2 and S3, quantitative determination of iodine and boron in the BINs films was carried out using inductively coupled plasma-mass spectrometry (ICP-MS), and relative test

parameters are summarized in Table S4. A substantially higher phosphor-to-boron (approximately 0.02) molar ratio was observed compared with that of phosphor to iodine (approximately 10), consistent with the difference in processing time. Specifically, a relatively high iodine content was observed, which may constitute one of the key factors contributing to the markedly enhanced ISC process in the phosphorescent system."

Table R2 (Table S2). Boron content in the BINs@PVA films.

Sample	Sample weight (g)	Final volume (ml)	Dilution factor	B Concentration (μmol/g)	Molar ratio ($n_{Phosphor}$:n_B)
DCBN	0.0237	15	1500	1647767229	0.02
DFBN	0.0404	15	1500	1232466997	0.02
DPBN	0.0323	15	1500	842621259	0.02
DPCA	0.0419	15	1500	914777247	0.03

Table R3 (Table S3). Iodine content in the BINs@PVA films.

Sample	Sample weight (g)	Final volume (ml)	Dilution factor	I Concentration (μmol/g)	Molar ratio ($n_{Phosphor}$:n_I)
DCBN	0.0258	10	100	361634	82.82
DFBN	0.0431	10	100	942152	26.44
DPBN	0.0319	10	100	4698982	4.39
DPCA	0.0527	10	100	2260292	13.25

Note: The concentrations (c_ω) of iodine and boron in BINs@PVA films were quantified by ICP-MS. The BINs concentration in the precursor solution was 0.5 or 1.0 mg mL^{-1} (Fig. S16), while the PVA concentration was fixed at 45 mg mL^{-1}. Accordingly, the BINs loading in the resulting films (c_ω) was estimated as shown below.

For DPBN and DFBN: $c_\omega = \frac{1000}{90}\ g/kg = \frac{10^9}{90}\ \mu g/kg$

For DCBN and DPCA: $c_\omega = \frac{1000}{45}\ g/kg = \frac{10^9}{45}\ \mu g/kg$

ICP-MS analysis indicates the molecular weights of DPBN, DFBN, DCBN and DPCA are 539.1 g mol^{-1}, 446.1 g mol^{-1}, 741.2 g mol^{-1} and 742.2 g mol^{-1}, respectively. Based on these values, the molar ratio can be calculated.

Table R4 (Table S4). Test parameters of ICP-MS.

Parameter	Value
Sample depth	5 mm
RF power	1550 W
Integration time	0.1 s
Nebulizer chamber temperature	2.5 °C
Integrating time	0.1 s
Plasma gas flow	15 L/min
Auxiliary gass flow	0.78 L/min
Detection mode	KED

Note: Owing to the manually operated nature of the ISB process and the intrinsic variation in the loading capacities of different BINs films toward boron and iodine species, substantial discrepancies in elemental contents were observed among the four films. Nevertheless, it can be reliably concluded that variations within this range exert a negligible influence on the phosphorescence performance of the BINs films. This parameter set was consistently identified through dozens of repeated trials during both the exploratory and characterization stages, demonstrating satisfactory reproducibility and reliability. In future work, the processing protocol will be further optimized to ensure consistent and well-controlled fabrication parameters across all films.

8. TGA results suggest that both untreated and ISB-treated films contain significant amounts of water. Optimizing the preparation protocol to reduce moisture content could further enhance material performance.

Response: We thank the reviewer for this valuable suggestion. Prior to performing the TGA measurements, the BINs@PVA films were first dried in a vacuum oven at 60 °C for 12 hours to remove most of the free water. According to your suggestion, we have further dried the BINs@PVA films at 120 °C under vacuum for 12 hours before testing. The results indicated that the T_d values of the BINs@PVA films exhibited negligible differences under these two treated conditions (Fig. R15), indicating that the moisture content is crucially low.

In addition, our previous systematic investigation (*Angew. Chem. Int. Ed*. 2024, 63, e202411323) has elucidated the critical influence of thermal annealing on the phosphorescence performance of phosphors embedded in PVA matrices. Specifically, we demonstrated that even brief annealing at 120 °C for 30 minutes can induce significant dehydration-induced crosslinking within PVA, thereby markedly altering its phosphorescent behavior (Fig. R16). Subsequently, we compared the afterglow photographs of the films treated under these conditions, and the afterglow performance of the BINs@PVA films is indeed enhanced at some extent, indicating that the reviewer’s suggestion is effective (Fig. R17).

However, compared to the post-ISB DFBN@PVA film annealed at 120 °C, the ISB-treated DFBN@PVA film subjected to drying at 60 °C for 24 hours exhibits virtually no discernible changes in its afterglow characteristics. This compelling observation indicates that the ISB-induced cross-linked network is already sufficiently developed, rendering that further thermal annealing is unnecessary in this work.

It is important to emphasize that the ISB strategy constitutes the central innovation of the present work. Further thermal annealing would undermine a clear assessment of the ISB strategy as the key factor for the luminescence enhancement.

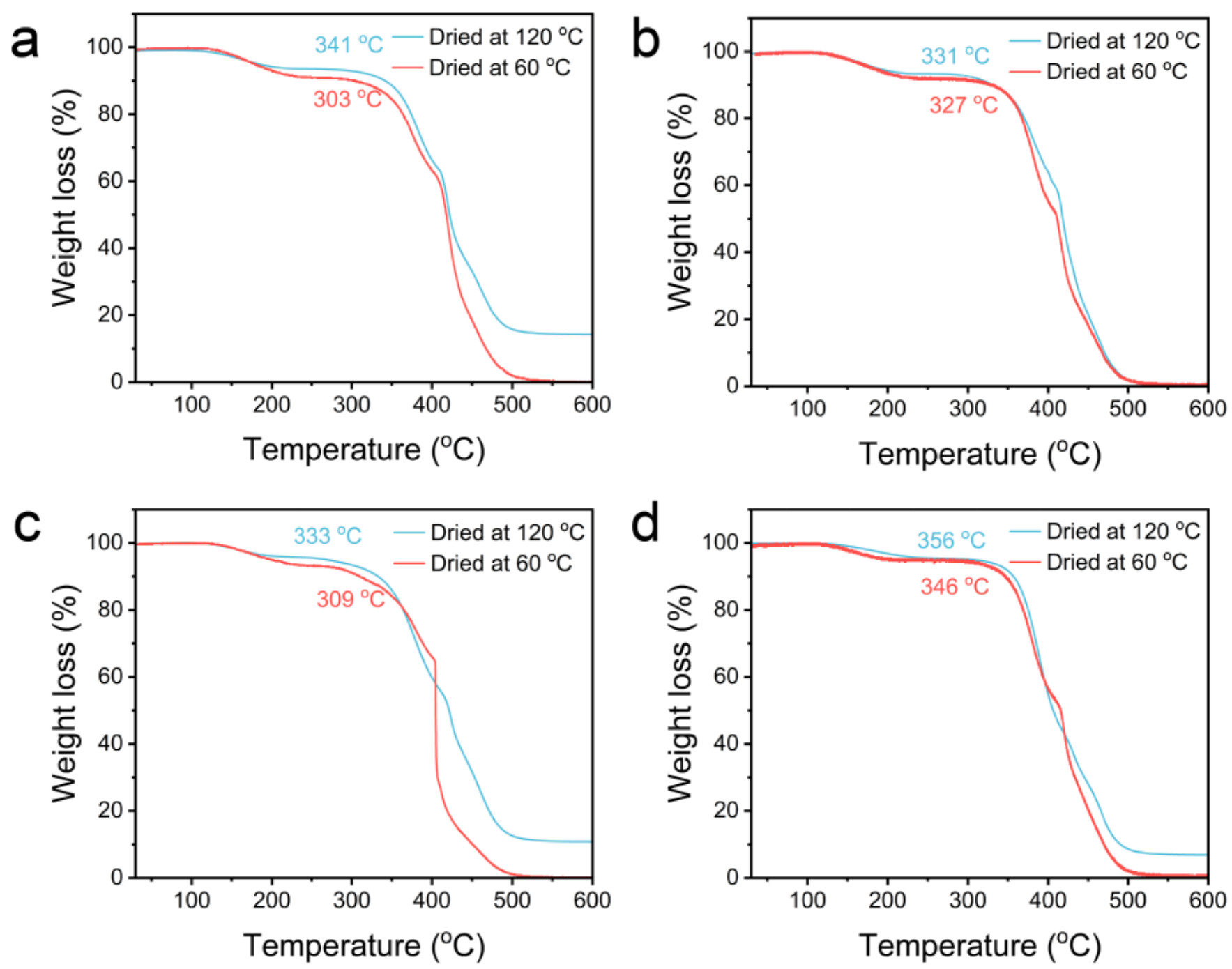

Fig. R15. TGA curves of (a) DPBN@PVA, (b) DFBN@PVA, (c) DCBN@PVA, and (d) DPCA@PVA films obtained under different drying conditions. Note: BINs@PVA films dried at 60 °C refer to the original data, whereas those dried at 120 °C denotes samples sequentially dried at 60 °C for 24 h and then at 120 °C for 12 h.

[*REDACTED*]

Fig. R16. (a) Phosphorescence spectra of 9HMF@PVA after thermal annealing for 30 minutes at various temperature. (b) Phosphorescence spectra and (c) Lifetime profiles of 9HMF@PVA with thermal activation. Cited from *Angew. Chem. Int. Ed*. 2024, 63, e202411323.

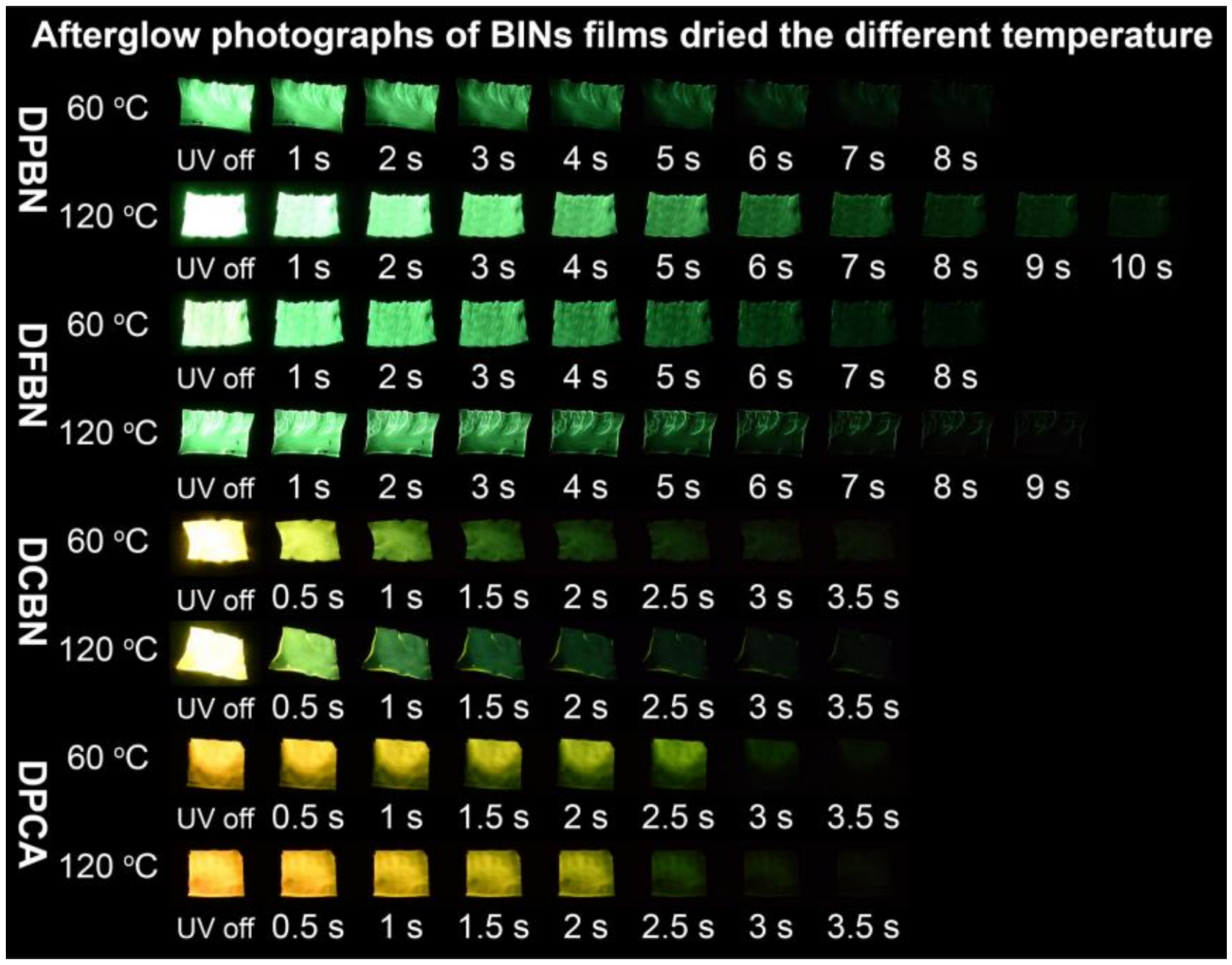

Fig. R17. Afterglow photographs of post-ISB BINs@PVA films under 365 nm UV excitation after thermal annealing at 60 °C and 120 °C for 30 min.

9. In Fig. 5d, please describe in detail how the computational model was constructed, especially how the position of I_3^- was determined.

Response: We sincerely appreciate your detailed review and thoughtful feedback. To validate our computational model, we first considered the literature reports (Refs 36 and 41 in Manuscript), which indicate that the I_3^- ions are arrayed randomly in the one-dimensional column parallel to the PVA chain axis (Fig. R18). The optimized structure of our isolated PVA-KI_3 model (Fig. 5a) closely resembles the reported complex II, and our calculated total interaction energy between PVA and KI_3 (-6.69 kcal/mol) is in good agreement with the literature value (-9.11 kcal/mol), supporting the suitability of our model.

Secondly, to elucidate the distinct roles of PVA and KI_3 in modulating electron distribution and enhancing intersystem crossing (ISC) in DFBN, we recalculated the spin-orbit coupling (SOC) constants and the energy levels of the S_1 and T_1 states. These calculations were performed for DFBN, DFBN@PVA, DFBN-KI_3, and DFBN-KI_3@PVA models based on their S_0 optimized geometries. As shown in Fig. R19 (Fig. 5), PVA primarily alters the electron distribution between the S_1 and T_1 states of DFBN. In contrast, KI_3 enhances the ISC efficiency through a dual mechanism: it significantly increases the SOC constants while concurrently reducing the energy gap between S_1 and T_1 state.

Based on our calculations, the specific position of the I_3^- ion does not alter the conclusion that the heavy-atom effect enhances the intersystem crossing (ISC) process. To ensure computational feasibility, we therefore employed isolated molecular models rather than computationally expensive aggregate models. To enhance visual appealing and improve the clarity of our

presentation, we have replotted the calculated results. These are now presented in revised manuscript and Supporting Information.

[*REDACTED*]

Fig. R18. (a) Crystal structure of PVA-I_3^- complex proposed by the X-ray analysis. (b) DFT optimized structure. Cited from *Polymer* 2016, 99, 566-579.

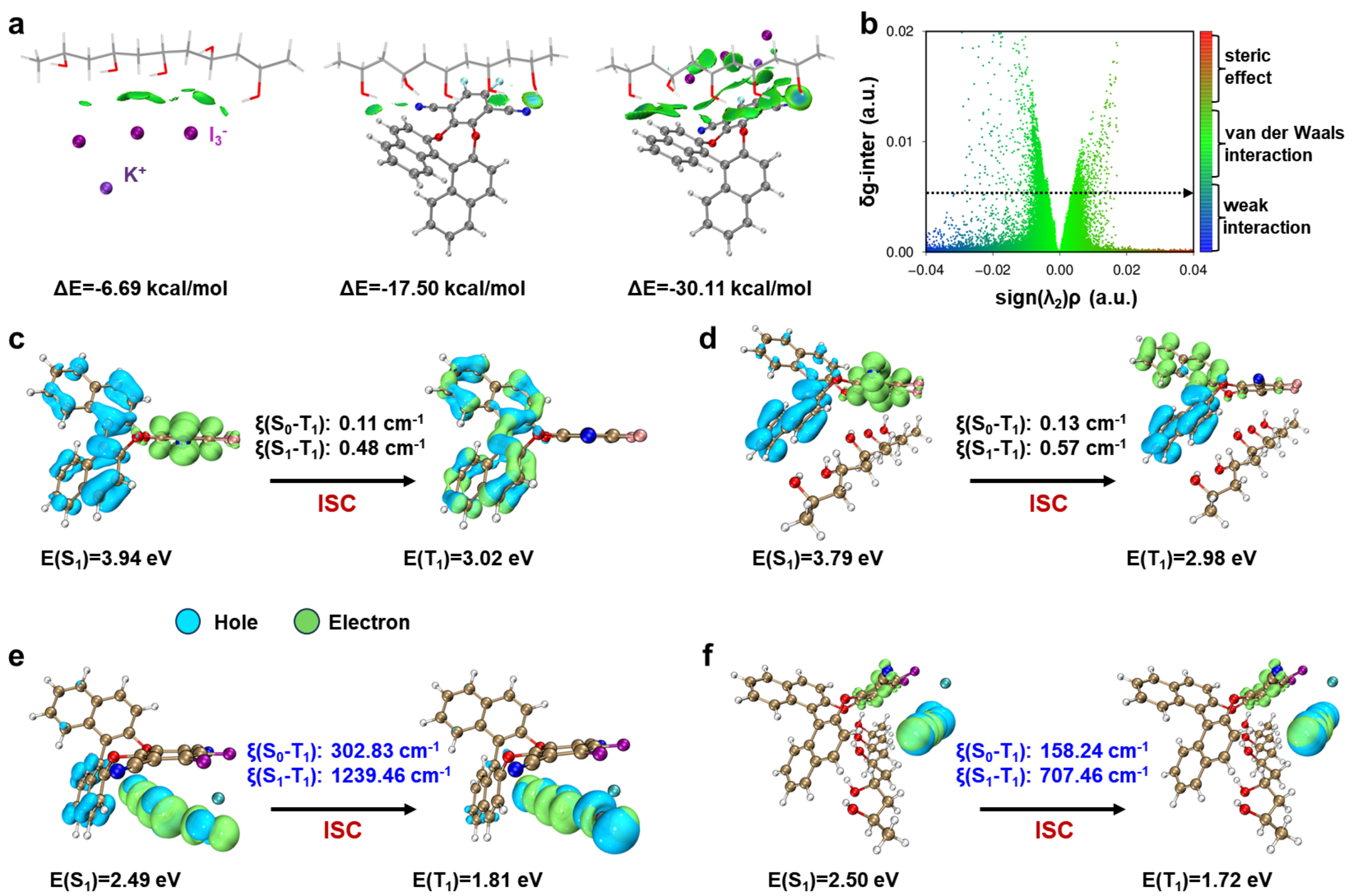

Fig. R19 (Fig. 5). Mechanism study of ISB enhancing RTP via theory calculations. (a) Intermolecular interaction energies (considered on BSSE corrections). **(b)** Distribution of different interactions between DFBN and KI_3@PVA by IGMH scatter map. Electron-hole analysis, SOC

constants, and energy levels of the S_1 and T_1 states of **(c)** DFBN, **(d)** DFBN@PVA, **(e)** DFBN-KI_3, and **(f)** DFBN-KI_3@PVA models based on their S_0 optimized geometry.

10. All reported ^{1}H NMR spectra are inadequate. Full spectra, not just downfield regions, must be shown.

Response: Thank you for your valuable suggestions. Accordingly, we reconducted ^{1}H NMR spectra and revised them in the Supporting Information, as shown in Fig. R20 to Fig. R23.

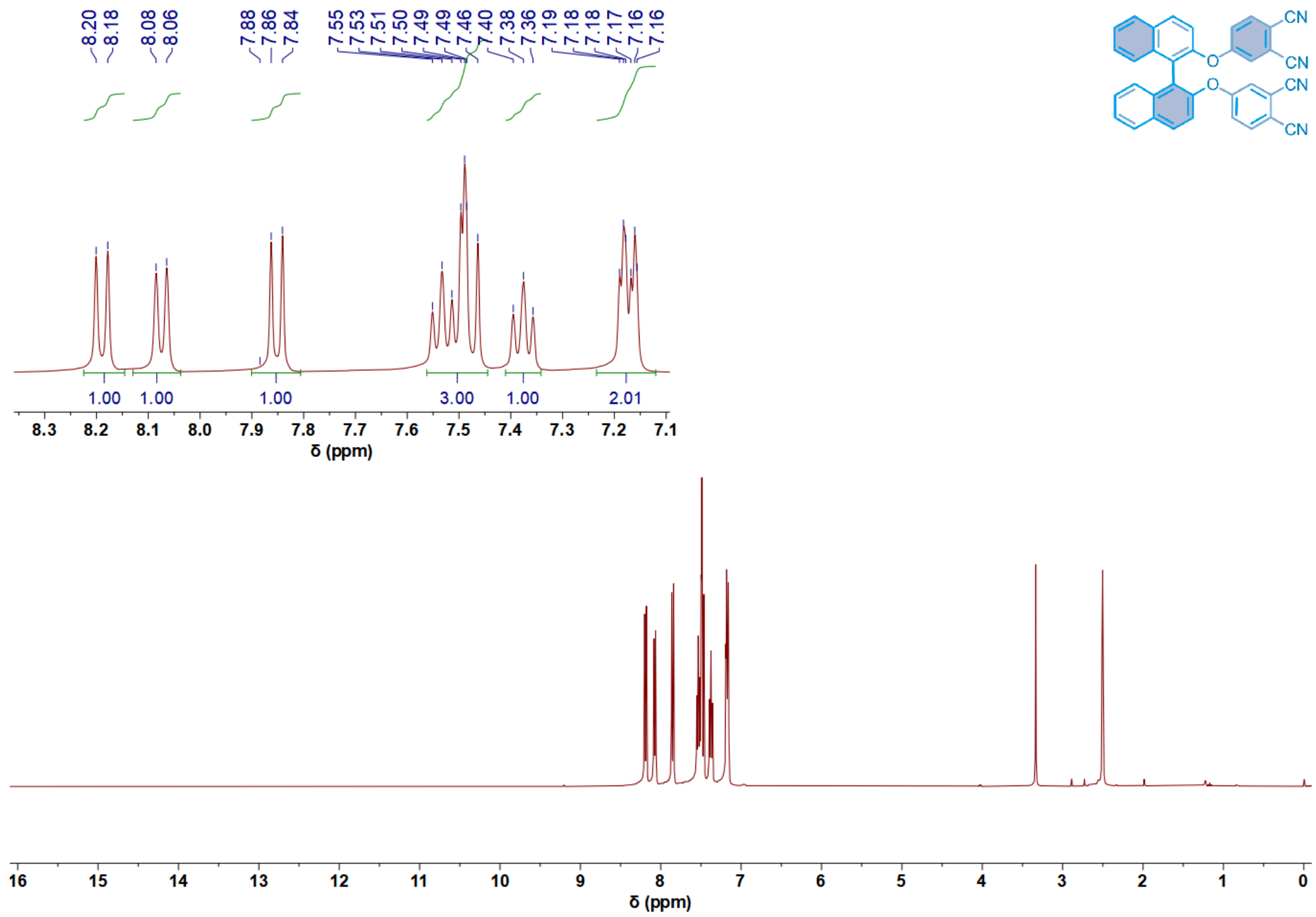

Fig. R20 (Fig. S1). ^{1}H NMR spectrum of DPBN molecule in DMSO-d_6.

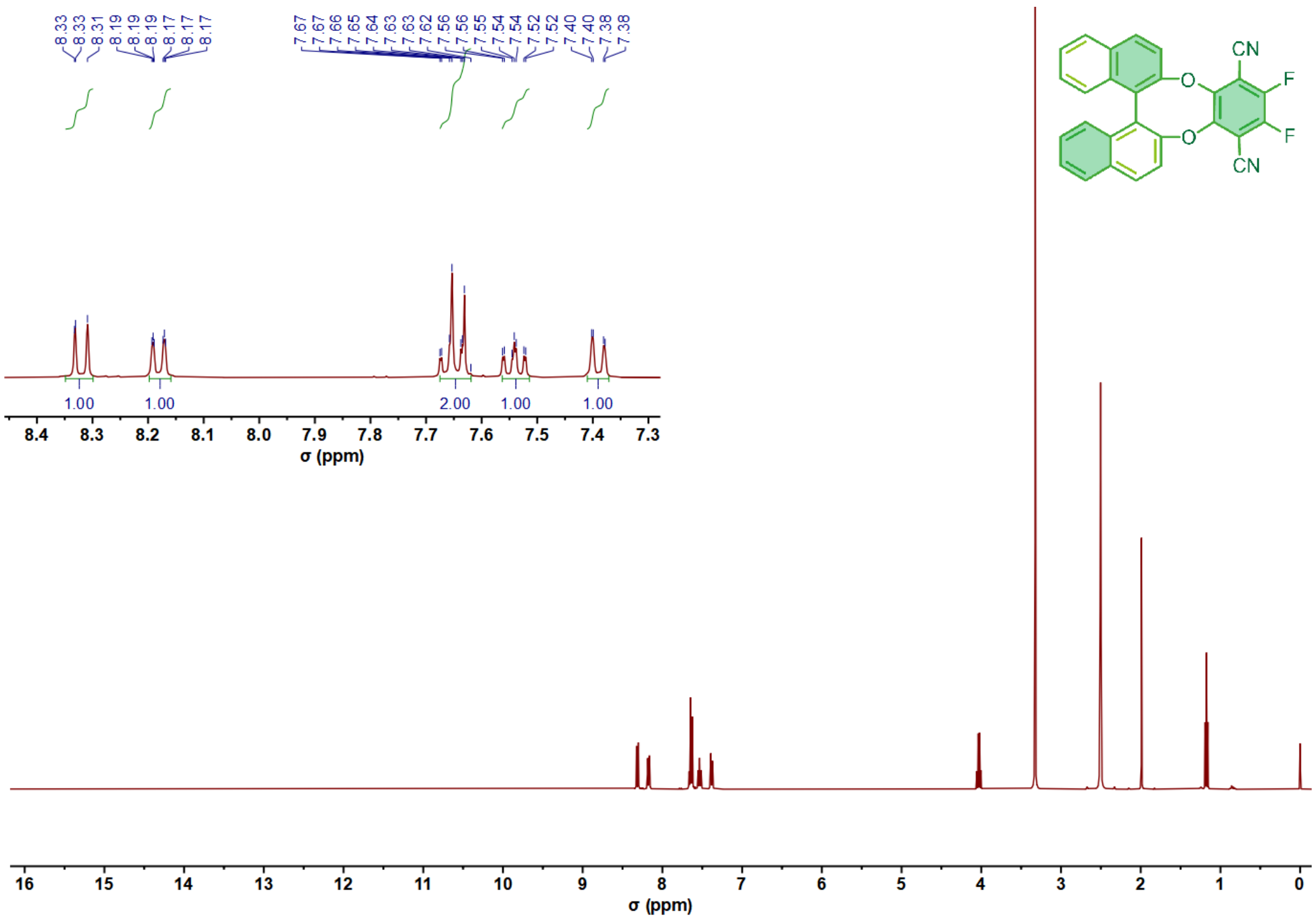

Fig. R21 (Fig. S2). ^{1}H NMR spectrum of DFBN molecule in DMSO-d_6.

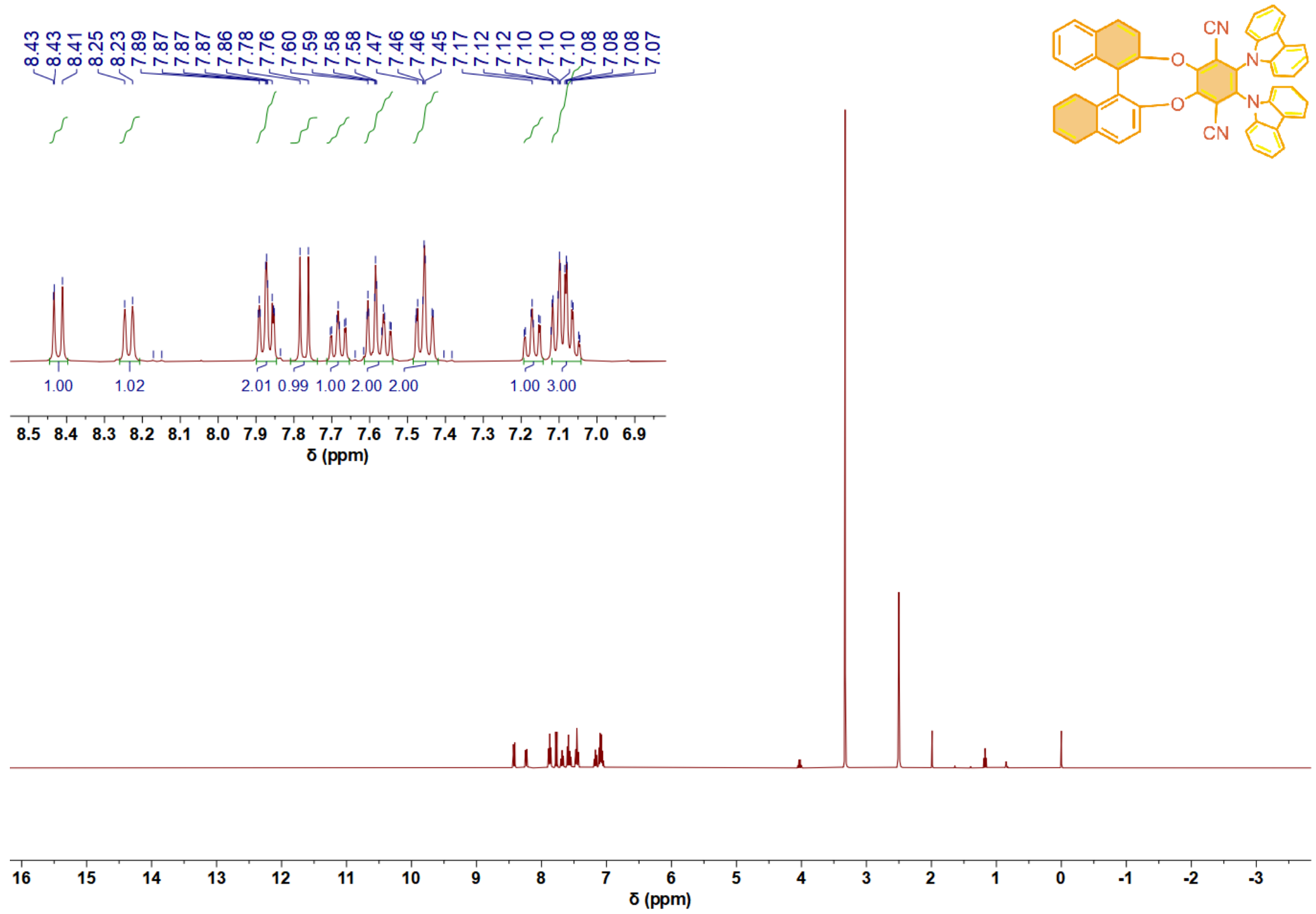

Fig. R22 (Fig. S3). ^{1}H NMR spectrum of DCBN molecule in DMSO-d_6.

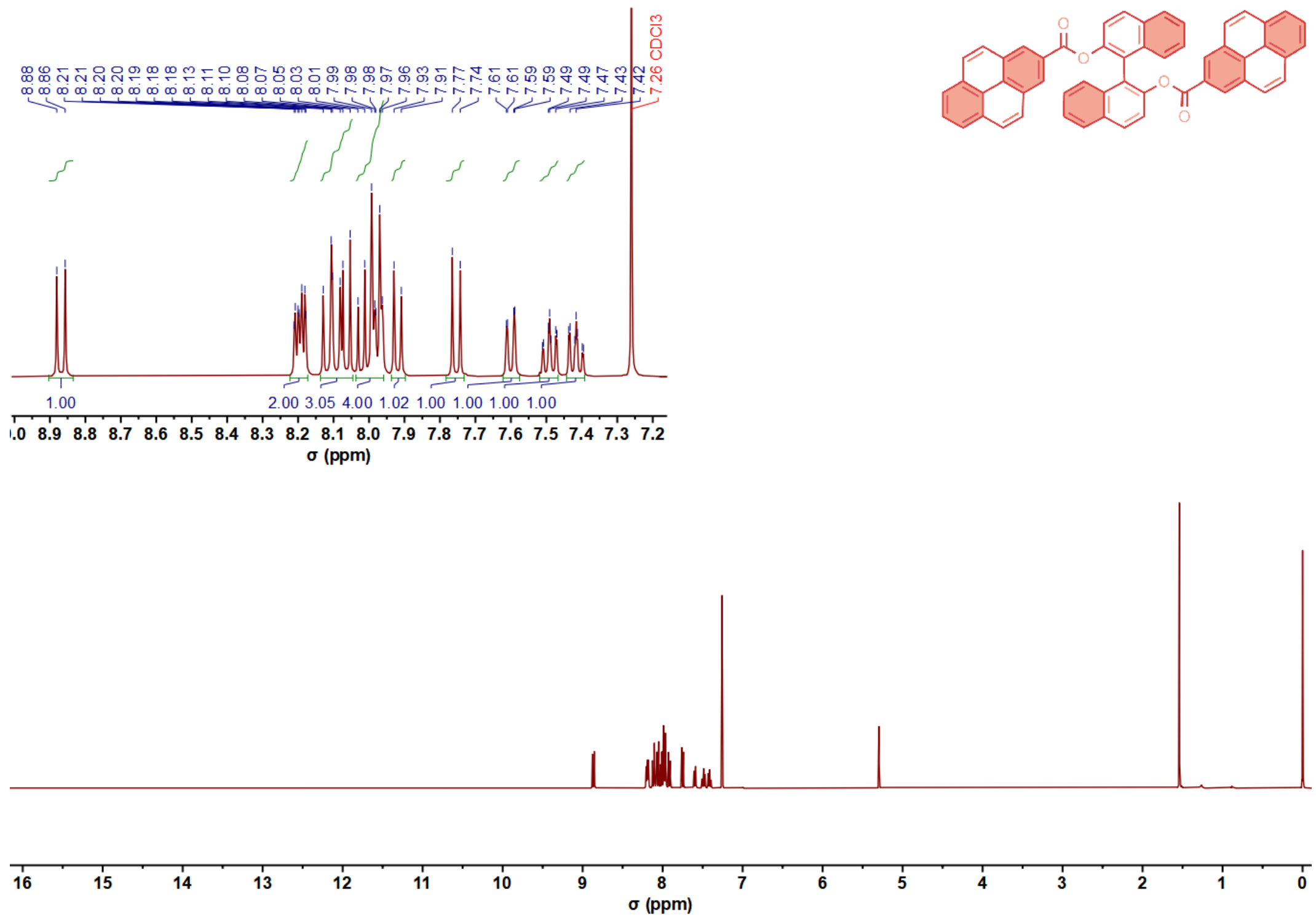

Fig. R23 (Fig. S4). ^{1}H NMR spectrum of DCBN molecule in $CDCl_3$.

11. Did BINs undergo degradation during ISB treatment? Please provide stability or decomposition tests to address this concern.

Response: We sincerely appreciate your detailed review and thoughtful feedback. To address your concern, we first evaluated the thermal stability of the four BIN molecules using TGA. As shown in Fig. R24, the decomposition temperature exceeds 300 °C, indicating that the materials are thermally stable and unlikely to degrade during the ISB treatment where the maximum processing temperature is 70 °C.

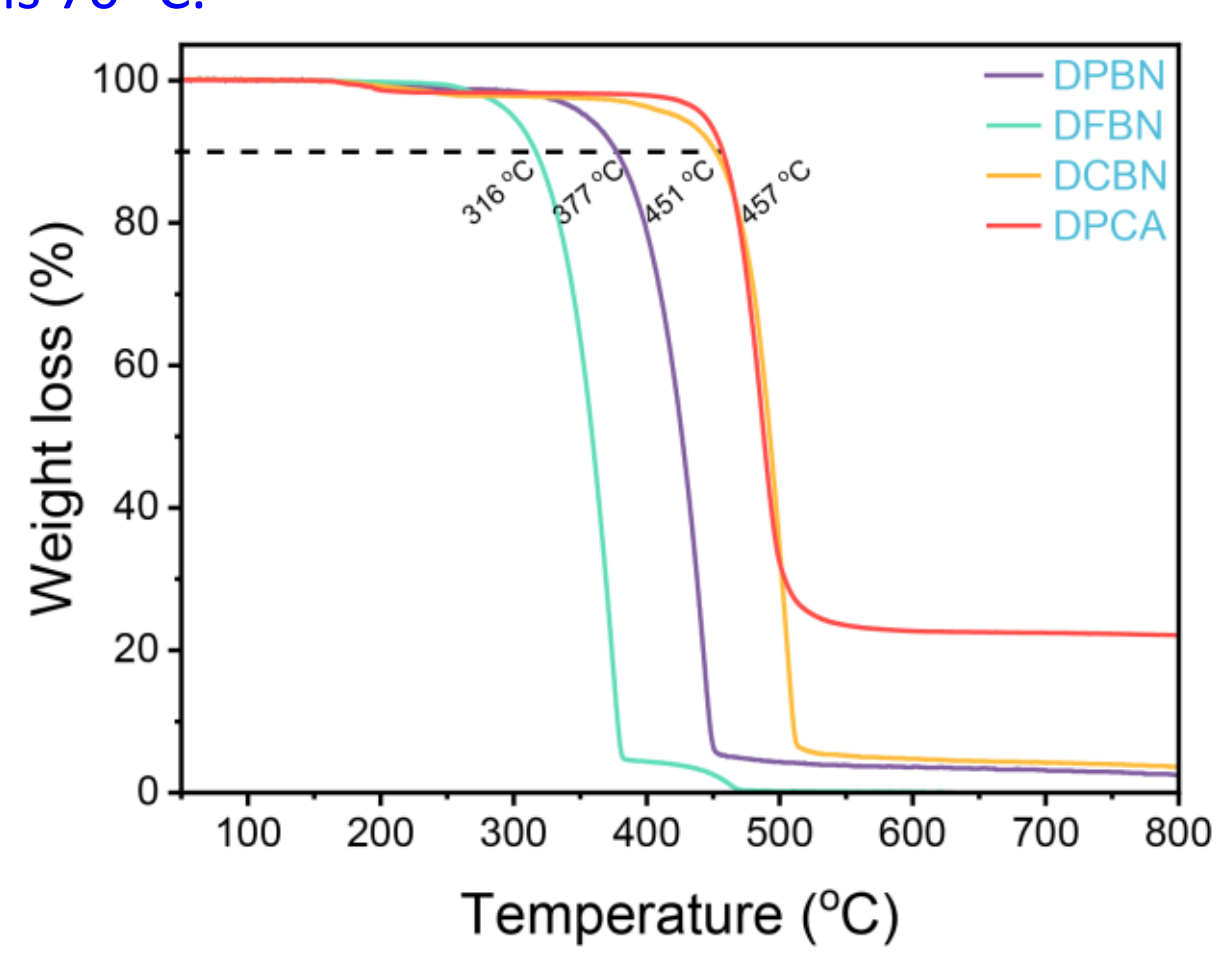

Fig. R24 (Fig. S42). TGA curves of the four BIN molecules.

Secondly, based on the ICP-MS results (Table R2-R3), boric acid, BIN molecules, and iodine were mixed at a molar ratio of 50:1:0.1. In order to simulate the ISB treatment conditions, BINs were subsequently heated at 70 °C for 2 min prior to ^{1}H NMR characterization or thoroughly ground to ensure homogeneity before FT-IR tests. As shown in Fig. R25 and Fig. R26, the ^{1}H NMR spectra exhibit well-matched integral ratios for all proton signals, and the FT-IR spectra preserve the characteristic absorption bands at about 2230 cm^{-1}, attributable to the C≡N stretching vibrations of DPBN, DFBN and DCBN, together with a distinct band at about 1720 cm^{-1}, arising from the C=O stretching vibrations. Collectively, these results confirm that the weak oxidative nature of iodine does not lead to any detectable structural damage to the BIN molecules.

In addition, XPS was employed to further assess the structural stability of the BINs@PVA films. As shown in Fig. R27, the N 1s signal of the ISB-treated films is significantly attenuated. This attenuation is attributed to enhanced electrostatic interactions between the PVA matrix and the -CN groups, which are induced by the ISB treatment rather than any structural degradation of the phosphors. This interpretation is fully consistent with the IGMH analysis results presented in Figs. 5a-5b and Figs. S37-S39.

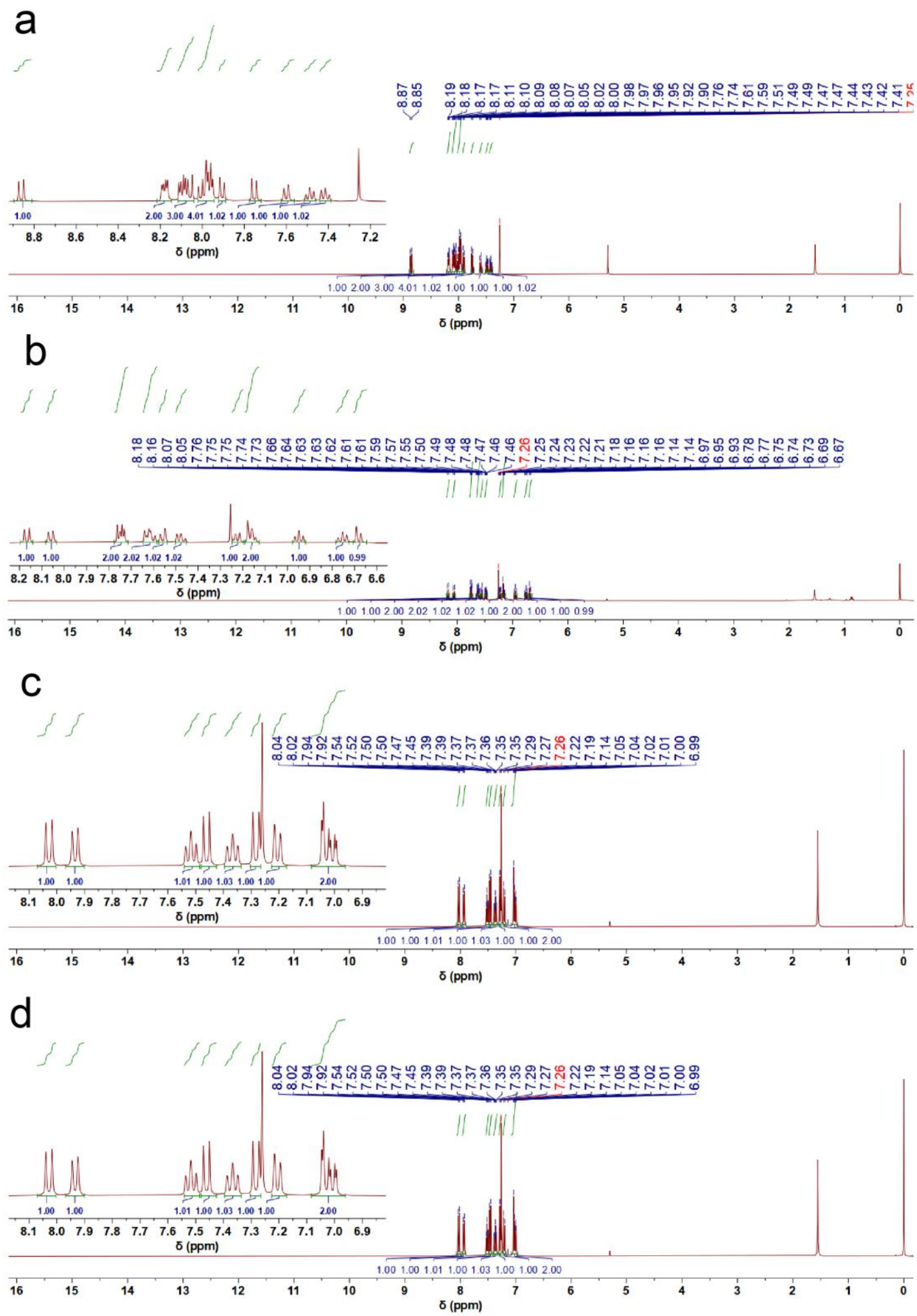

Fig. R25 (Fig. S43). ^{1}H NMR spectra of (a) DPBN, (b) DFBN, (c) DCBN, and (d) DPCA in $CDCl_3$. Each sample was heated at 70 °C for 2 min prior to measurement.

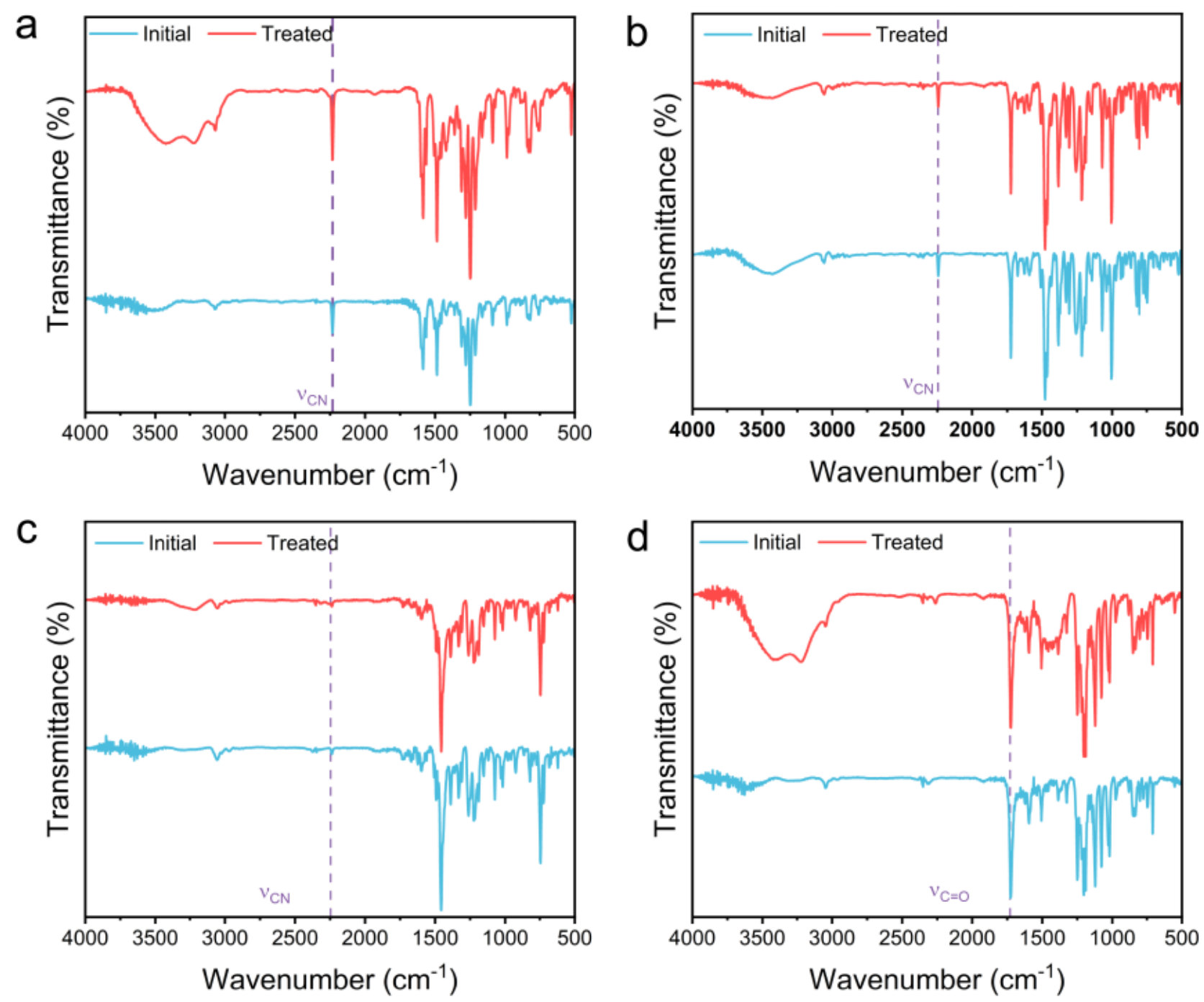

Fig. R26 (Fig. S44). FT-IR spectra of (a) DPBN, (b) DFBN, (c) DCBN, and (d) DPCA powder in the initial state and after treatment.

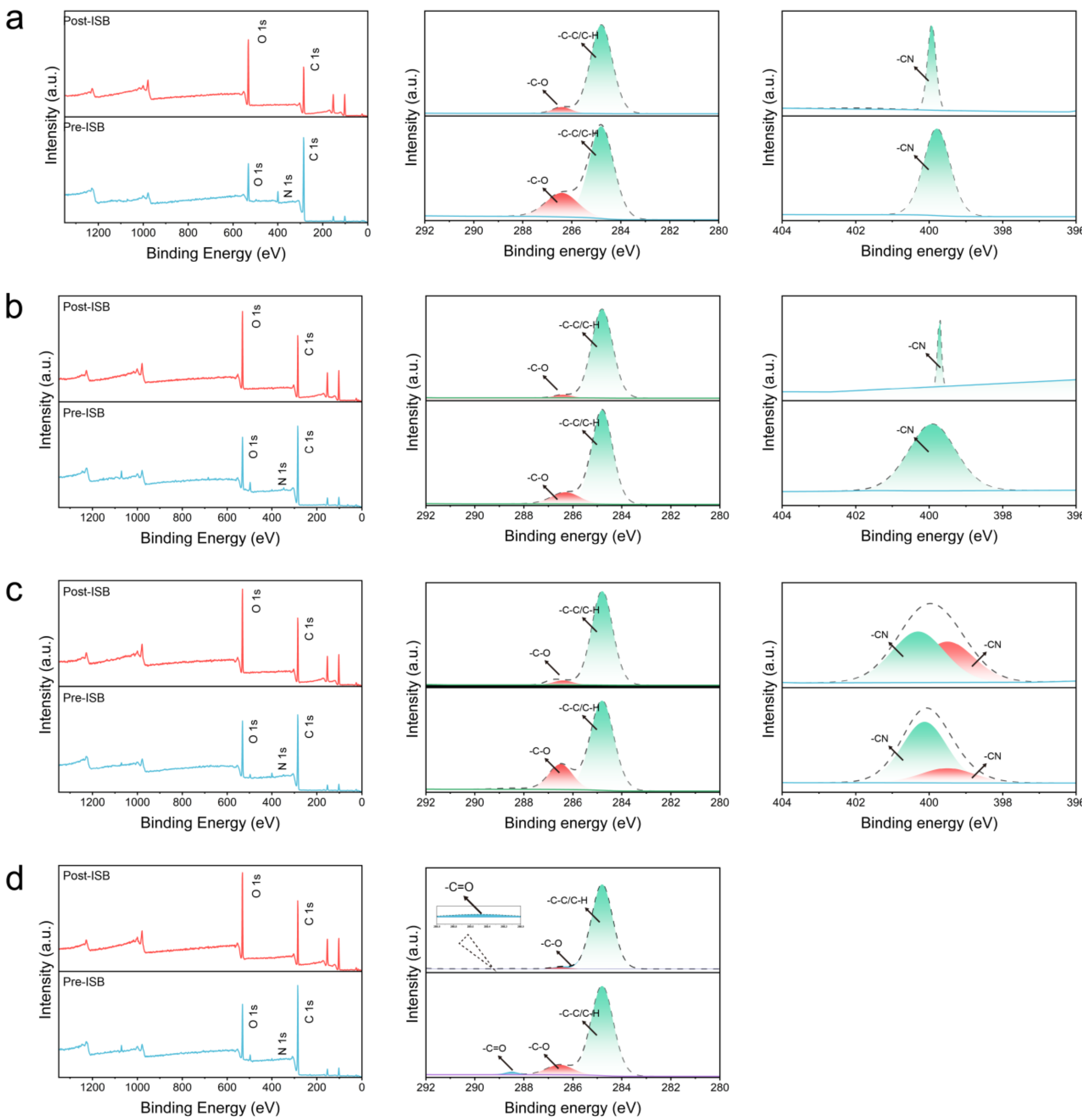

Fig. R27 (Fig. S45). Raw XPS data and corresponding deconvoluted C 1s and N 1s spectra of (a) DPBN@PVA, (b) DFBN@PVA, (c) DCBN@PVA, and (d) DPCA@PVA films before and after ISB treatment.

Reviewer #3 (Remarks to the Author):

In this manuscript, the authors report that the phosphorescence properties of organic molecules are significantly improved by "ISB" treatment, defined as the stretching of PVA films followed by impregnation with aqueous iodine and boric acid solutions. Experimental and theoretical analyses revealed that the ISB treatment enhances the RTP properties of the dyes through the external heavy atom effect and the orientation of PVA chains. Since ISB treatment is a simple method widely used in the production of polarizing plates, the discovery that such treatment leads to improved phosphorescence properties is highly interesting. Therefore, I recommend the acceptance of this manuscript after minor revisions.

Response: Thank you for your valuable feedback and the careful comments. We have fully incorporated your suggestions in the revised manuscript.

1. The authors label PL spectra as "fluorescence spectra" or "phosphorescence spectra." However, in several instances, both components are clearly observed in the data, making these labels technically inaccurate. The authors should adopt more precise terminology, such as "steady-state PL spectra" or "time-gated PL spectra," to accurately reflect the measurement conditions.

Response: We sincerely appreciate your detailed review and thoughtful feedback. We recognize that the previous labeling lacked precision, and have therefore revised them according to your suggestion. The respective delay times for the delayed spectra were also included.

2. Please specify the monitored wavelengths for the PL decay curves presented in the manuscript.

Response: We thank the reviewer for this valuable advice. We have specified the monitored wavelength in Figs. S17, S18, S23, S24, S40, S41 and S46, and the corresponding descriptions of the PL decay curves have been revised.

3. Please provide a detailed description in the Experimental section regarding how the fluorescence and phosphorescence quantum yields (Φ_F and Φ_P) were determined. Additionally, please clarify the calculation methods used to derive the rate constants.

Response: Many thanks for your advice. The quantum yields mentioned in this work are absolute quantum yields, and relative raw data can be seen in Figs. S21 and S22. In addition, we have supplied the calculation methods in "Calculation of photophysical constants" section.

REVIEWER COMMENTS

Reviewer #1 (Remarks to the Author):

The authors have addressed all concerns. The manuscript is now recommended for publication in Nature Communications.

Response: We are very grateful to the recommendation of publication.

Reviewer #3 (Remarks to the Author):

Although the authors have generally addressed the comments, several points require further clarification or action:

Response: We thank you for your useful comments. The concerns are addressed point by point below.

1. The authors refer to the steady-state PL spectra in Figure S18 as "prompt spectra"; however, this term does not accurately signify steady-state conditions. It must be clarified whether the spectra in Figure S18 represent time-gated PL spectra (where long-lived components have been excluded) or steady-state spectra obtained under continuous excitation.

Response: We sincerely thank you for your insightful and constructive comments, which have greatly improved our manuscript. The photoluminescence spectra shown in this work were indeed obtained under steady-state conditions, not from time-gated measurements. Therefore, we have corrected the corresponding descriptions in the Manuscript and SI, and the relevant changes have been highlighted.

2. Experimental Section: In calculating the rate constants, the authors derived the subsequent equations under the assumption that k_{nr}^{P} is sufficiently smaller than k_{r}^{P}. However, as shown in Supplementary Table 1, the calculated k_{nr}^{P} is significantly larger than k_{r}^{P}, directly contradicting this premise. Furthermore, as shown in Figure 23, both the phosphorescence intensity and lifetime exhibit clear temperature dependence. This indicates that k_{nr}^{P} is substantial even at room temperature; therefore, neglecting it in the derivation of the equations is not justifiable. Also, rate constants should be denoted by a lowercase "k" rather than an uppercase "K".

Response: We fully agree with your insightful suggestion. Although many studies in RTP field estimate kinetic parameters under the assumption that $k_{nr}^{P}<<k_{r}^{P}$, BINs @PVA does not satisfy this condition, as evidenced by the calculated results and Figure S23. Therefore, this approximation is not applicable. Generally, the internal conversion (IC) between S_1 and S_0 is negligible if the energy

gap is larger than 2 eV (*J. Chem. Phys.* **1967**, 46, 440-447; *J. Chem. Phys.* **1967**, 47, 2411-2422). Since the energy gap between S_1 and S_0 is calculated using the equation $\Delta E_{S1\rightarrow S0} = 1240/\lambda$, this implies that if the fluorescence emission wavelength is less than 620 nm, the excited singlet state predominantly relaxes via fluorescence emission and ISC. In this work, BINs@PVA films satisfy this assumption according to Figure S18. Under this framework, the equation $\Phi_{ISC} = 1 - \Phi_F$ can be reasonably derived, and the k_r^F, k_r^P, k_{nr}^P, and k_{ISC} can be reasonably derived. Specific data are shown in Table R1 and the detailed derivation can be found at the section of 'Kinetic calculations and photophysical rate constants' in the revised manuscript. In addition, the rate constants are now denoted by k.

Sample			Prompt photoluminescence				Delayed photoluminescence			
		Φ_{PL} (%)	τ_F ns	Φ_F (%)	k_r^F (s^{-1})	k_{ISC} (s^{-1})	τ_P (ms)	Φ_P (%)	k_r^P (s^{-1})	k_{nr}^P (s^{-1})
DPBN@PVA	Pre-ISB	26.5	4.3	39.3	9.14×10^7	1.41×10^8	944.4	4.7	0.08	0.98
	Post-ISB	26.5	5.2	12.3	2.37×10^7	1.69×10^8	1690.3	14.2	0.1	0.50
DFBN@PVA	Pre-ISB	11.6	7.6	10.2	1.34×10^7	1.18×10^8	43.7	1.4	0.37	22.53
	Post-ISB	34.5	8.0	0.7	8.75×10^5	1.24×10^8	1228.4	33.8	0.28	0.54
DCBN@PVA	Pre-ISB	2.1	3.8	1.2	3.16×10^6	2.60×10^8	47	0.9	0.19	21.09
	Post-ISB	7.0	10.4	1.9	1.83×10^6	9.43×10^7	293.6	5.1	0.18	3.23
DPCA@PVA	Pre-ISB	23.1	27.2	22.6	8.31×10^6	2.85×10^7	30	0.5	0.22	33.12
	Post-ISB	22.9	29.7	18.5	6.23×10^6	2.74×10^7	205.6	4.4	0.26	4.60

Table R1 (Supplementary Table 1). Kinetic calculations and photophysical rate constants of pre-ISB and post-ISB treated BINs@PVA films.

In addition to the points above, I have reviewed the authors' responses to Reviewer 2. Overall, I believe the authors have addressed Reviewer 2's comments appropriately. However, a persistent concern remains regarding whether the authors are correctly distinguishing between steady-state and time-resolved data. The following points must be addressed in a revised version:

Response: We thank you for your careful evaluation of our response to Reviewer 2. Your concerns are addressed point by point below.

1. As noted in my previous comments, the "Prompt spectra" shown in Figure S18 do not generally represent steady-state spectra. The manuscript must clearly state in the Experimental Section which specific values were measured and provide the exact methodology used for those measurements. Furthermore, for all time-gated spectra, the specific gate width (time window) must be clearly defined.

Response: Thank you for your valuable suggestions. As noted above, we agree with your suggestion and have revised the manuscript accordingly. In addition, we have updated the Methods Section to include descriptions of the measurements performed using the Edinburgh FLS 1000. To clarify the conditions of the time-gated measurements, we have added the following statement: 'All spectra acquired under microsecond flash lamp excitation were collected with a 2 ms delay and an 8 ms gate' in the Methods Section. Detailed information can be found in page 23 of the revised manuscript.

2. While the revised Experimental Section suggests that Φ_F and Φ_P were determined through direct measurement, it remains unclear how Φ_F was specifically isolated. Figure S21 appears to show Φ_{PL}, which is fundamentally different from Φ_F. The authors must clarify this distinction and the calculation process.

Response: We thank you for your valuable suggestion and apologize for the insufficient methodological description in the original manuscript. In general, the total photoluminescence quantum yield (Φ_{PL}) can be expressed as the sum of phosphorescence (Φ_P), fluorescence (Φ_F), and delayed fluorescence contributions. For BINs@PVA, thermally activated delayed fluorescence (TADF) has been excluded based on temperature-dependent spectroscopic measurements (Figure S23). Therefore, Φ_{PL} can be considered as the sum of Φ_P and Φ_F.

Experimentally, the absolute Φ_{PL} was measured using an integrating sphere with a xenon lamp. The absolute Φ_P was determined using the same instrument equipped with a microsecond flash lamp on the Edinburgh FLS 1000 spectrometer, enabling temporal discrimination of phosphorescence. Accordingly, the Φ_F was obtained by subtracting Φ_P from Φ_{PL}.

This clarification has been added to Methods Section and the note of Figure S21. Detailed information can be found in pages 23-24 of the revised manuscript.

3. Measuring the absolute Φ_P using a time-gating method is technically demanding. Was the calibration for the excitation pulse intensity performed correctly to ensure absolute values? The authors should provide the detailed calculation formulas in the Experimental Section, explaining how photons emitted outside the gate window were integrated and normalized to obtain the absolute Φ_P.

Response: We thank the reviewer for raising this important point. In this work, the absolute Φ_{PL} was measured using an integrating sphere equipped in the FLS1000 spectrometer. In this method, the number of absorbed excitation photons is determined by comparing the excitation light intensity with and without the sample inside the integrating sphere. Therefore, the calibration of excitation pulse intensity is inherently accounted for, and no external calibration is required. The calculations are defined as equation 1. This approach is widely recognized as a reliable method for determining absolute quantum yields.

$$\Phi_{PL} = \frac{L_{sam}}{E_{ref} - E_{sam}} \qquad (1)$$

where E_{ref} represents the excitation light intensity measured in the absence of the sample, E_{sam} represents the excitation light intensity measured in the presence of the sample (i.e., the unabsorbed excitation light), L_{sam} represents the emission intensity of the sample, and the denominator (E_{ref} - E_{sam}) denotes the absorbed excitation photons.

The time-gating technique was employed only to separate the phosphorescence component from the total emission. Specifically, the time-gated spectrum was collected with a 2 ms delay and an 8 ms gate under the microsecond flash-lamp excitation to isolate

phosphorescence, while the absolute quantum yield was still determined based on the integrating sphere method.

We have clarified this point and added the corresponding description in the Experimental Section. Detailed information can be found in page 23 of the revised manuscript.

4. Please clarify whether the Φ values used to calculate the photophysical rate constants (k_{ISC}, k_r, k_{nr}) in Table S1 and throughout the text refer to Φ_{PL} or Φ_F. It is essential to re-verify the mathematical consistency of these calculations based on the correct definitions of each quantum yield.

Response: We thank you for the careful consideration of our manuscript. The responses to questions 2 and 4 might have already addressed the concern. We are sure that the quantum yield definitions have been clarified, and the data have been verified to be consistent.

REVIEWERS' COMMENTS

Reviewer #3 (Remarks to the Author):

The authors have appropriately addressed the reviewers' concerns. However, one correction is required prior to acceptance. While the authors calculate ΦISC = 1 – ΦF on the grounds that the S0–S1 energy gaps are sufficiently large, the cited literature specifically targets simple aromatic hydrocarbons, and this theory is not universally applicable to all types of luminophores. Therefore, the description in the Experimental Section must be revised to clarify that this theory was originally proposed for aromatic hydrocarbons and that the authors are assuming its validity for their specific system in order to derive these values.

Response: Thanks for your valuable suggestions. We have revised the description in the Experimental Section, and relative change is shown below:

"Since the energy gap between S_1 and S_0 is calculated using the equation $\Delta E_{S1\rightarrow S0} = 1240/\lambda$, this implies that if the fluorescence emission wavelength is less than 620 nm, the excited singlet state predominantly relaxes via fluorescence emission and ISC. In this work, *BINs@PVA* films satisfy this assumption according to Supplementary Fig. 18."
